# Formyl-selective deuteration of aldehydes with D_2_O *via* synergistic organic and photoredox catalysis†

**DOI:** 10.1039/c9sc05132e

**Published:** 2019-12-04

**Authors:** Jianyang Dong, Xiaochen Wang, Zhen Wang, Hongjian Song, Yuxiu Liu, Qingmin Wang

**Affiliations:** State Key Laboratory of Elemento-Organic Chemistry, Research Institute of Elemento-Organic Chemistry, College of Chemistry, Nankai University Tianjin 300071 People's Republic of China wangqm@nankai.edu.cn

## Abstract

Formyl-selective deuteration of aldehydes is of high interest for labeling purposes and for optimizing properties of drug candidates. Herein, we report a mild general method for formyl-selective deuterium labeling of aldehydes with D_2_O, an inexpensive deuterium source, *via* a synergistic combination of light-driven, polyoxometalate-facilitated hydrogen atom transfer and thiol catalysis. This highly efficient, scalable reaction showed excellent deuterium incorporation, a broad substrate scope, and excellent functional group tolerance and selectivity and is therefore a practical method for late-stage modification of synthetic intermediates in medicinal chemistry and for generating libraries of deuterated compounds.

## Abstract 2

Formyl-selective deuteration of aldehydes with D_2_O mediated by the synergistic combination of light-driven, polyoxometalate-facilitated HAT and thiol catalysis is reported.

## Graphical abstract

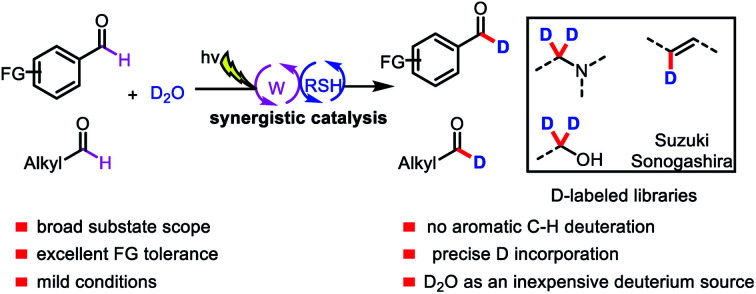

## Introduction

Deuterium labeling has a range of applications, including in the investigation of reaction mechanisms^1^ and the analysis of drug absorption, distribution, metabolism, and excretion,^2^ as well as in nuclear magnetic resonance spectroscopy^3^ and mass spectrometry.^4^ In recent years, interest in the incorporation of deuterium atoms into patented drugs and drug candidates to enhance their metabolism and pharmacokinetic properties has burgeoned.^5^ In 2017, the US Food and Drug Administration approved the first deuterated drug, deutetrabenazine (Austedo),^6^ and the increasing demand for new deuterium-labeled drugs has motivated the development of efficient deuteration methods.^7^

**Scheme 1:**
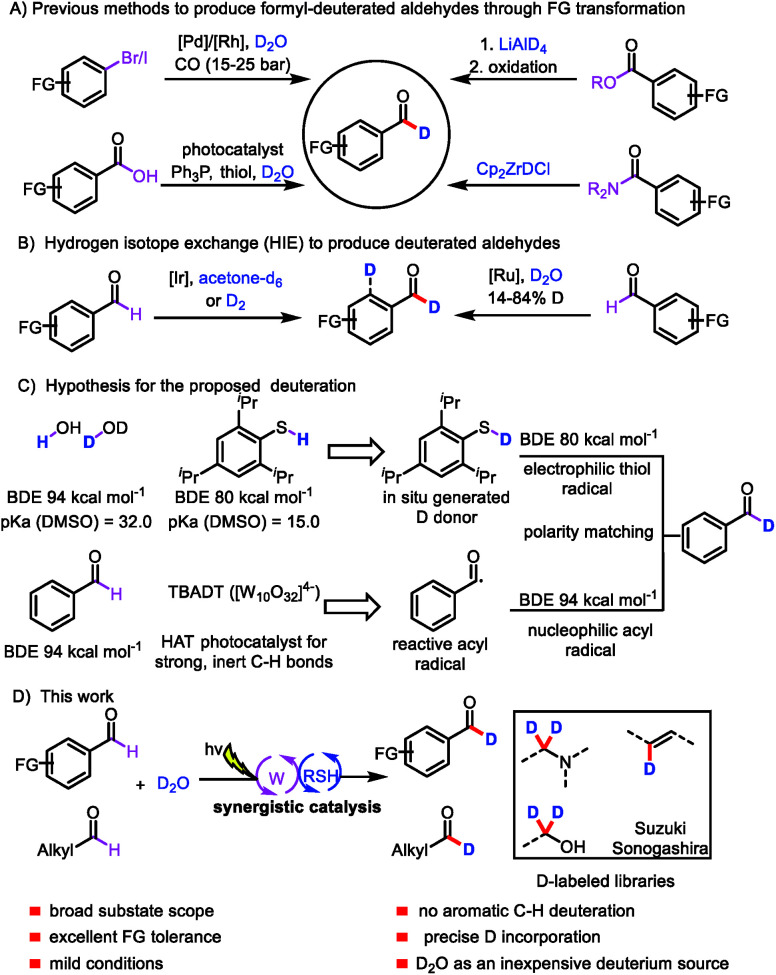
Strategies for synthesizing deuterated aldehydes; (A) previous methods to produce deuterated aldehydes through FG transformation; (B) hydrogen isotope exchange (HIE) to produce deuterated aldehydes; (C) hypothesis for the proposed deuteration; (D) our work.

Aromatic aldehydes are often used as building blocks for pharmaceutical synthesis owing to their versatile reactivity;^8^ indeed, they can be rapidly transformed to other compounds *via* C–C and C–X bond forming reactions. The development of an efficient protocol for constructing formyl-deuterated aromatic aldehydes can be expected to increase the availability of deuterated lead compounds. Aromatic aldehydes selectively labeled at the formyl position are traditionally produced from the corresponding esters by means of reduction with LiAlD_4_ followed by oxidation,^9^ from the corresponding amides by reaction with deuterated Schwartz's reagent (obtained from LiAlD_4_),^10^ from aryl halides *via* Pd/Rh-cocatalyzed reductive carbonylation,^11^ or from carboxylic acids *via* deoxygenative deuteration with synergistic photoredox and organic catalysis (Scheme 1A).^12^ In terms of atom- and step-economy, the ideal protocol for preparing deuterated aromatic aldehydes would be direct hydrogen isotope exchange (HIE) at the formyl C–H bond. In fact, a few protocols for Ir- and Ru-catalyzed HIE at the formyl moiety have been reported, although there is an intrinsic difficulty in controlling the reactivity of the aryl ring moiety and formyl moiety (Scheme 1B).^13^ Moreover, late-stage introduction of deuterium into structurally complex aldehydes remains a challenge. Given the importance of deuterated aldehydes, we were interested in developing a method for efficient, atom- and step-economical HIE with the goal of achieving formyl-selective labeling of aldehydes with D_2_O, which is an inexpensive deuterium source. In addition, we wanted the new HIE method to be useful for late-stage introduction of deuterium into structurally complex aldehydes.

Visible-light-mediated photoredox catalysis has recently emerged as a method for achieving organic transformations *via* radical processes.^14^ Recently, MacMillan's group reported direct HIE at α-amino C(sp^3^)–H bonds *via* a pathway involving abstraction of a deuterium atom from a deuterium-labeled thiol catalyst by an α-amino radical.^15^ We hypothesized that generation of acyl radicals from aldehydes by a hydrogen atom transfer (HAT) photocatalyst in the presence of D_2_O and a thiol catalyst would produce deuterated aldehydes.

We envisioned that polyoxometalates, many of which possess high-energy excited states that can accomplish the desired C–H abstraction,^16^ would be ideal HAT catalysts for the proposed transformation. We were particularly interested in the decatungstate anion ([W_10_O_32_]^4−^), an efficient HAT photocatalyst that has been widely used for oxygenation, dehydrogenation, conjugate addition, and, more recently, fluorination and arylation of strong, unactivated aliphatic C–H bonds, which have bond dissociation energies (BDEs) of up to 100 kcal mol^−1^ (for cyclohexane).^17^ To our knowledge, visible-light polyoxometalate-facilitated HAT has not previously been synergistically merged with thiol catalysis. Because aldehydes have relatively low BDEs (94 kcal mol^−1^),^12^ we envisioned that such a combination of catalytic processes would afford access to a considerable variety of acyl radicals and deuterated aldehydes from abundant aldehyde feedstocks (Scheme 1C). Moreover, owing to the gaps between the BDEs of C(=O)H bonds (94 kcal mol^−1^), aryl C–H bonds (113 kcal mol^−1^), and S–H bonds (80–88 kcal mol^−1^), the decatungstate anion ([W_10_O_32_]^4−^) would be unable to abstract a hydrogen atom from the aryl C–H bond,^17*g*^ allowing us to achieve formyl labeling without the formation of aryl-labeled by-products (Scheme 1C). In contrast, the nucleophilic acyl radical would readily undergo HAT from the thiol catalyst to form a deuterated aldehyde, owing to the gap between the BDEs of the C–H and S–H bonds and a polarity matching effect (Scheme 1C).^18^ We recognized that the choice of a suitable thiol catalyst would be heavily influenced by thermodynamic factors, particularly the BDE of the thiol bond relative to that of the acyl C–H bond, as well as its p*K*_a_ relative to that of water. Herein, we describe a general strategy for formyl-selective deuteration of aldehydes with D_2_O mediated by the synergistic combination of light-driven, polyoxometalate-facilitated HAT and thiol catalysis (Scheme 1D).

## Results and discussion

**Scheme 2:**
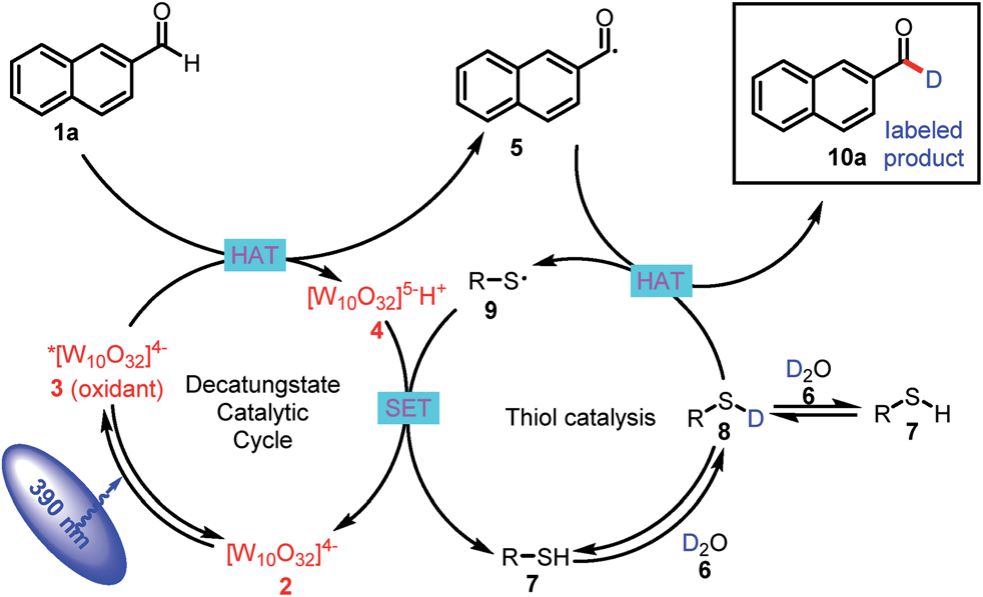
Proposed mechanism.

The proposed mechanism for formyl-selective deuteration of aldehydes is shown in Scheme 2. In this mechanism photoexcitation of tetrabutylammonium decatungstate (TBADT, **2**) followed by intersystem crossing produces triplet excited state **3**, which has a lifetime of 55 ns.^19^ Subsequent selective abstraction of the formyl H atom of 2-naphthaldehyde (**1a**) affords singly reduced decatungstate **4** and reactive nucleophilic formyl radical **5**. At the same time, thiol HAT catalyst **7** undergoes exchange with D_2_O to give deuterated thiol **8**, which serves as the source of deuterium. Because of the gap between the reported BDEs for typical formyl C–H and thiol S–H bonds, we reasoned that HAT between polarity-matched radical **5** and deuterated thiol **8** would furnish deuterated aldehyde **10a** and electrophilic thiol radical **9** [C(=O)–H BDE = 94 kcal mol^−1^; S–H BDE = 80 kcal mol^−1^ for **7**]. The two catalytic cycles converge when reduced decatungstate **4** undergoes a single-electron transfer reaction with thiol radical **9** to regenerate active HAT photocatalyst **2** and thiol HAT catalyst **7**.

To test our mechanistic hypothesis, we began by exploring deuteration reactions of 2-naphthaldehyde (**1a**) under various conditions (Table 1). First, several thiol catalysts were screened in the presence of 4 mol% of commercially available TBADT as the HAT photocatalyst in 1 : 1 (v/v) dichloromethane/D_2_O under irradiation with near-ultraviolet light (36 W 390 nm light-emitting diodes; see the ESI†). To our delight, 2-naphthaldehyde (**10a**) with 94% deuterium incorporation was obtained when 2,4,6-triisopropylbenzenethiol (**7a**, 40 mol%) was used as the thiol catalyst (entry 1). Under the same conditions, other thiols gave considerably lower deuterium incorporation percentages (entries 2–4). The use of *N*-methyl-2-pyrrolidinone, acetonitrile, or chloroform as the cosolvent also led to decreased deuterium incorporation (entries 5–7). Reducing the amount of TBADT or thiol **7a** slightly decreased the deuterium incorporation percentage (entries 8 and 9). Control experiments showed that the reaction failed to proceed in the absence of the HAT photocatalyst, the thiol catalyst, or light (entries 10–12).

**Table 1:** Optimization of conditions for the catalytic formyl-selective deuteration reaction^a^

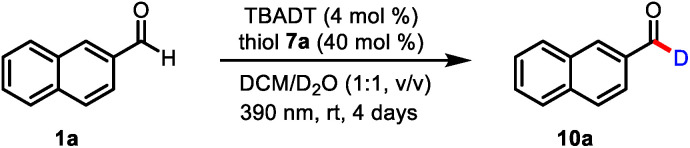		
Entry	Deviation from standard conditions	Deuteration^b^ (%)
1	None	94
2	**7b** instead of **7a**	63
3	**7c** instead of **7a**	71
4	**7d** instead of **7a**	78
5	NMP instead of DCM	13
6	MeCN instead of DCM	38
7	CHCl_3_ instead of DCM	62
8	2 mol% of TBADT	77
9	20 mol% of **7a**	80
10	No TBADT	<5
11	No **7a**	<5
12	No light	<5
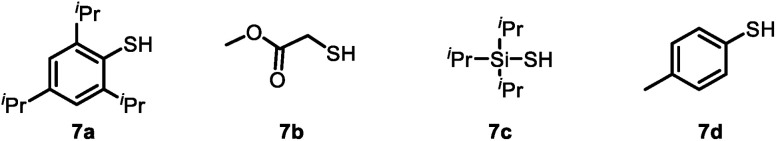		

a Reaction conditions, unless otherwise noted: **1a** (0.3 mmol), TBADT (0.012 mmol), **7a** (0.12 mmol), and 1 : 1 (v/v) DCM/D_2_O (3.0 mL) under an Ar atmosphere.

b Deuterium incorporation was determined by integration of the residual formyl proton in the ^1^H NMR spectrum. DCM = dichloromethane and NMP = *N*-methyl-2-pyrrolidinone.

With the optimized conditions in hand, we elucidated the scope of the transformation with respect to the aldehyde. Deuterated 2-naphthaldehyde **10a** was isolated in 92% yield after purification by column chromatography, indicating that decomposition and side-product formation were minimal. 6-Methoxy-2-naphthaldehyde was also a suitable substrate, giving **10b** in 90% yield with 92% deuterium incorporation. 1-Naphthaldehyde gave a product (**10c**) with lower deuterium incorporation (90%) than **10a**, presumably because the 1-position is more sterically hindered than the 2-position. We also tested benzaldehydes bearing substituents with various electronic and steric properties to obtain deuterated products **10d–10bb**. Specifically, benzaldehydes with electronically neutral functional groups, such as isopropyl, *t*-butyl, and phenyl, worked well under the optimal conditions, giving high yields with excellent deuterium incorporation (**10d–10f**, 93%). Halogenated molecules account for approximately 50% of the market-leading drugs because they are less susceptible to oxidation by cytochrome P450.^20^ Furthermore, they offer a valuable platform for generating molecular complexity through cross-coupling reactions.^21^ Therefore, we were encouraged to find that benzaldehydes bearing fluoro, chloro, bromo, and iodo atoms gave the corresponding products (**10g–10k**) with high deuterium incorporation (90–96%). Trifluoromethyl and trifluoromethoxy groups, which have often been shown to improve the activity of pharmaceutical leads, were compatible with our protocol, showing good deuterium incorporation (**10l–10o**, 95–97%) regardless of their position on the benzene ring. Benzaldehydes with other electron-withdrawing groups (ester and cyano) were smoothly transformed into the corresponding deuterated aldehydes (**10p** and **10q**). Moreover, electron-rich aldehydes were also reactive (**10r–10v**, 88–95% deuterium incorporation). Intriguingly, several relatively sensitive yet versatile functional groups—boronic esters (**10w** and **10x**), an alkyne (**10y**), and an alkene (**10z**)—tolerated the deuteration conditions well, which shows the potential utility of this protocol for synthetic and medicinal chemistry applications. Polysubstituted aldehydes were also suitable substrates (**10aa** and **10bb**). Being aware of the important role of heteroaromatic moieties in the scaffolds of pharmaceutical compounds, we were pleased to find that N- and S-heterocycles were also tolerated under our deuteration conditions (**10cc–10gg**). In addition, the reaction was amenable to scale up; when it was carried out on a 9 mmol scale, **10i** was isolated in 93% yield with no decrease in deuterium incorporation.

In addition to aromatic aldehydes, aliphatic aldehydes are also interesting because they constitute an even greater part of the –CHO family. However, few radical-based tools for convenient modification of aliphatic aldehydes have been developed. This is particularly true for branched aldehydes, which tend to undergo decarbonylation under radical conditions.^22^ However, when we subjected linear and branched aldehydes to our protocol, we found that all of them underwent formyl deuteration to afford the desired products (**10hh–10mm**, 90–98% deuterium incorporation), and no products of CO dissociation were observed. However, a limitation of this reaction was also uncovered. That is, deuteration also takes place at the α-positions of formyl.

This excellent functional group tolerance suggested that the protocol would be useful for the synthesis of structurally complex deuterated aldehydes, and indeed we were pleased to find that deuterated aldehydes derived from menthol, pregnenolone, ibuprofen, and fenbufen derivatives (**10nn–10qq**) could be accessed with uncompromised reactivities. In addition, the reaction of adapalene, an antiacne drug, afforded corresponding deuterated aldehyde **10rr** with high deuterium incorporation. These examples confirmed the potential utility of our protocol for practical late-stage modification of synthetic intermediates in medicinal chemistry applications (Table 2).

**Table 2:** Exploration of substrate scope^a^

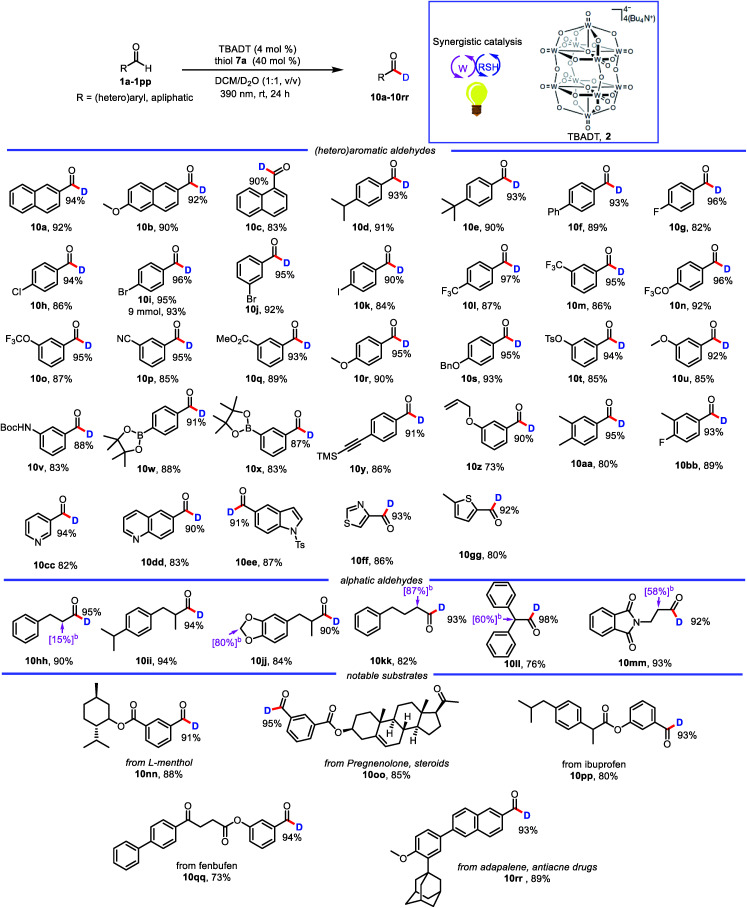

a Reactions were performed on a 0.3 mmol scale, unless otherwise noted.

b Some sites in compounds **10hh**, **10jj**, **10kk**, **10ll** and **10mm** (shown in their structures, with the deuteration percentage in square brackets) are deuterated. Isolated yields are given. See the ESI for experimental details. Deuterium incorporation was determined by integration of the residual formyl proton in the ^1^H NMR spectra.

**Scheme 3:**
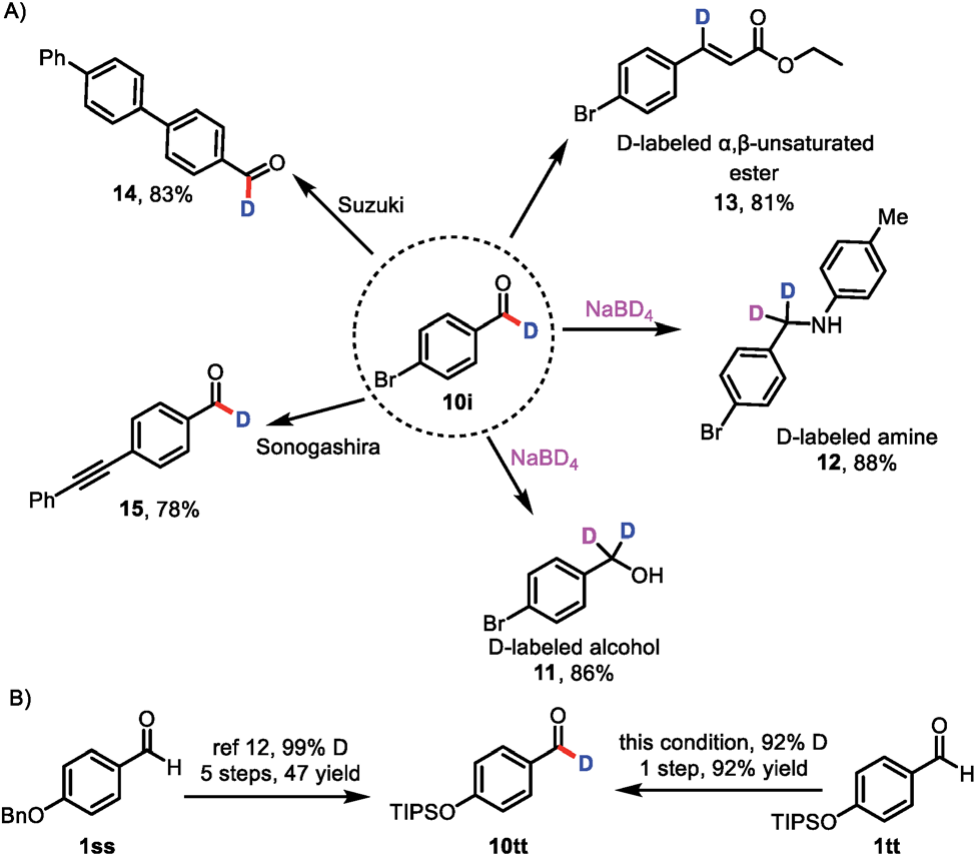
Transformations of deuterated aldehyde **10i** and improved synthetic route to deuterium-labeled aldehyde **10tt**; (A) **10i** is used to access libraries of deuterated compounds; (B) improved synthetic route to deuterium-labeled aldehyde **10tt**.

Because aldehydes are versatile functional groups that undergo a wide variety of organic transformations, the protocol reported herein can be used to access libraries of deuterated compounds (Scheme 3A). For example, deuterium-labeled aldehyde **10i** readily underwent reduction, reductive amination, and Horner–Wadsworth–Emmons olefination to deliver deuterated alcohol **11**, deuterated amine **12**,^7*a*^ and β-deuterated, α,β-unsaturated ester **13**. Importantly, **13** has not been accessed by direct labeling of the corresponding cinnamate ester.^23^ Deuterium could also be incorporated into pharmaceutical molecules and advanced materials, as indicated by the successful Suzuki coupling reaction of **10i** to afford **14** and the Sonogashira coupling reaction of **10i** to provide **15**, which is difficult to obtain by currently available methods.

In a further demonstration of the utility of this catalytic formyl-deuteration method, we used it to synthesize silyl-protected phenol **10tt**, which is an intermediate in the synthesis of enantiopure d_1_-benzyl alcohols, in a single, high-yielding step and with a high deuterium incorporation from aldehyde **1tt** (Scheme 3B).^24^ This method is superior to the existing route to **10tt**, which employs stoichiometric quantities of reagents and requires five steps from aldehyde **1ss**.^24^

**Scheme 4:**
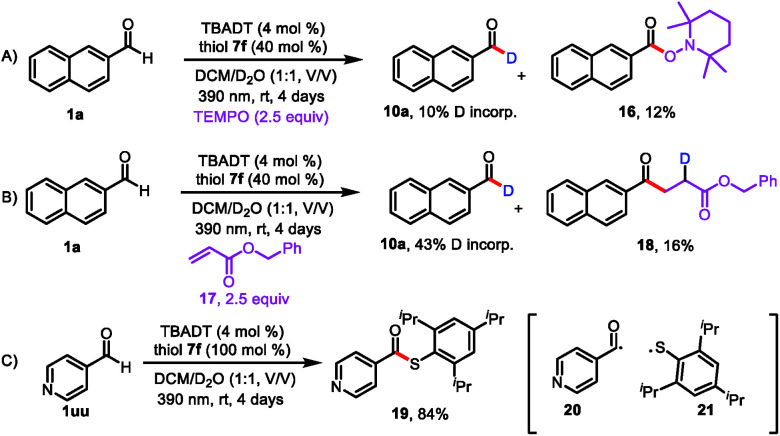
Mechanistic studies in support of the proposed pathway; (A) TEMPO was used as radical scavenger; (B) benzyl acrylate was used as radical scavenger; (C) 4-pyridinecarboxaldehyde was used as the substrate.

Having explored the substrate scope and utility of the reaction, we conducted mechanistic studies to support the proposed pathway shown in Scheme 2. When radical scavenger 2,2,6,6-tetramethyl-1-piperidinyloxy (TEMPO) was present in the reaction mixture, deuterium incorporation was markedly decreased (to 10%), and the radical-trapping product 2,2,6,6-tetramethylpiperidin-1-yl-2-naphthoate (**16**) was isolated in 12% yield (Scheme 4A). When benzyl acrylate (**17**) was used as a radical scavenger, benzyl 4-(naphthalen-2-yl)-4-oxobutanoate-2-*d* (**18**) was isolated (Scheme 4B). These results suggest that acyl radical **5** was generated. When we used 4-pyridinecarboxaldehyde (**1uu**) as the substrate, we isolated by-product **19** (84% yield), which was generated by a radical coupling reaction of acyl radical **20** and thiol radical **21** (Scheme 4C). These experiments clearly point to a radical pathway. Furthermore, the light on/off experiments illustrated a total interruption of the reaction process in the absence of light and recuperation of reactivity on further illumination (Fig. S3†). This indicates that light was essential for this transformation, and any chain propagation process should be short-lived.

## Conclusions

In conclusion, we have developed a mild protocol for selective direct HIE at the formyl C–H bonds of both aromatic and aliphatic aldehydes with D_2_O as an inexpensive deuterium source mediated by synergistic visible-light polyoxometalate-facilitated HAT catalysis and thiol catalysis. The high efficiency, broad substrate scope, excellent functional group tolerance, and mild conditions make this protocol practical for late-stage modification of pharmaceutical intermediates and for obtaining libraries of deuterated compounds.

## Conflicts of interest

There are no conflicts to declare.

## Supplementary Material

SC-011-C9SC05132E-s001
