# Supplementary material for: Formyl-selective deuteration of aldehydes with D2O via synergistic organic and photoredox catalysis
Source: Chem Sci. 2019 Dec 4;11(4):1026–31. doi: 10.1039/c9sc05132e (PMC8145436; doi:10.1039/c9sc05132e)

Electronic Supplementary Material (ESI) for Chemical Science.
This journal is © The Royal Society of Chemistry 2019

Formyl-Selective Deuteration of Aldehydes with D_2O via Synergistic Organic and Photoredox Catalysis

Jianyang Dong, Xiao-Chen Wang, Zhen Wang, Hongjian Song, Yuxiu Liu and Qingmin Wang*

State Key Laboratory of Elemento-Organic Chemistry, Research Institute of Elemento-Organic Chemistry, College of Chemistry, Nankai University, Tianjin 300071, China.

Supporting Information

Table of Contents……………………………..…………………………………S1
General Information…………………………………………………………………….S2
Preparation of photocatalyst tetrabutylammonium decatungstate (TBADT).…………………S3
Preparation of **1nn** and **1oo**……………………………………………………………S3
Preparation of **1pp** and **1qq**…………………………………………………………S3–S4
Reaction optimization………………………………………………………………S4–S6
Investigation of the mechanism…………………………………………………S6–S9
Experimental Procedures and Product Characterization…………………………S9–S26
Procedures for organic transformations…………………………………………S26–S29
Gram-scale Reaction………………………………………………………………S29
References……………………………..………………………..……S30
Copies of ^{1}H NMR and ^{13}C NMR spectra for new compounds……………….…S31–S83

1. General Information

Reagents were purchased from commercial sources and were used as received. ^1H and ^{13}C Nuclear Magnetic Resonance (NMR) spectra were recorded on Bruker Avance 400 Ultrashield NMR spectrometers. Chemical shifts (δ) were given in parts per million (ppm) and were measured downfield from internal tetramethylsilane. High-resolution mass spectrometry (HRMS) data were obtained on an FTICR-MS instrument (Ionspec 7.0 T). The melting points were determined on an X-4 microscope melting point apparatus and are uncorrected. Conversion was monitored by thin layer chromatography (TLC). Flash column chromatography was performed over silica gel (100-200 mesh). 390 nm LED (36 W) purchased from JIADENG (LS) was used for light irradiation. A fan attached to the apparatus was used to maintain the reaction temperature at room temperature.

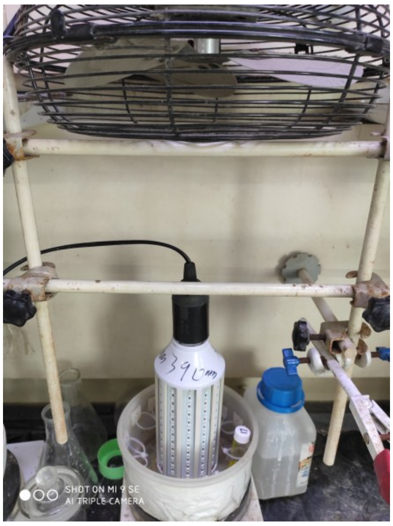

Figure S1 Photograph of the Photocatalytic reactor used for reactions conducted under 390 nm LED irradiation.

2. Preparation of photocatalyst tetrabutylammonium decatungstate (TBADT).

The photocatalyst was synthesized according to literature report.[1] To a 2 L beaker wrapped in aluminum foil for insulation and equipped with a 4" Teflon stir bar were added tetrabutylammonium bromide (4.80 g, 14.9 mmol, 0.49 equiv.) and deionized water (1600 mL). In a separate 4 L beaker wrapped in aluminum foil for insulation and equipped with a 4" Teflon stir bar were added $Na_2WO_4{\bullet}2H_2O$ (10 g, 30.3 mmol, 1.00 equiv.) and deionized water (1600 mL). Both solutions were rapidly stirred and heated to 90 °C. When both solutions reached 90 °C, concentrated HCl was added to each solution until pH stabilized at 2. At this point, the acidified solutions were combined in the 4 L beaker, and the resultant suspension was stirred at 90 °C for an additional 30 minutes. The reaction mixture was cooled to room temperature, then filtered through a pad of silica gel. The solids were washed with water and left to dry under vacuum. When the silica-supported solids were dry, the receiving flask was exchanged, and the pad was washed with 3 x 200 mL acetonitrile. The filtrate was collected and solvent was removed. The crude residue was thoroughly dried under vacuum, dissolved in minimal hot acetonitrile, then placed in the freezer at –20 °C for 12 hours. The solids were collected on a filter, washed with minimal cold acetonitrile, then dried under vacuum. The filtrate was reconcentrated, dissolved in minimal hot acetonitrile, and crystallized again to afford a second crop of TBADT. Isolated as pale yellow crystals (82% yield). UV-Vis and CV characterization is consistent with literature data[1].

3. Preparation of 1nn and 1oo.

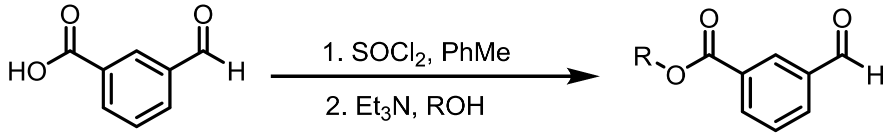

1nn and **1oo** were synthesized according to literature report,[2] a solution of 3-formylbenzoic acid (0.90 g, 6 mmol) and $SOCl_2$ (4.38 mL, 60 mmol) in toluene (60 mL) was refluxed for 1 h. Removal of the solvent under reduced pressure afforded crude 3-formylbenzoyl chloride in a quantitative yield. A solution of this chloride (1.0 g, 6 mmol) in dioxane (30 mL) was added dropwise to a solution of ROH (5 mmol) and triethylamine (0.84 mL, 6 mmol) in dioxane (50 mL). After the mixture was stirred at room temperature for 24 h, the solvent was removed under reduced pressure to give a residue that was purified by chromatography, affording the corresponding aromatic aldehydes. The spectral data is consistent with the literature data.[2]

4. Preparation of 1pp and 1qq.

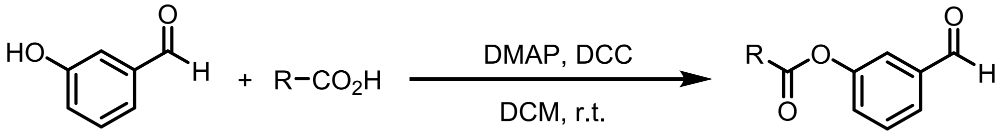

1pp and **1qq** were synthesized according to literature report,[3] m-hydroxybenzaldehyde (0.8 mmol, 97.6 mg) and acid (0.8 mmol, 1 equiv) and dry CH_2Cl_2 (20 mL) were added sequentially to a dry round-bottom flask at room temperature. The reaction was cooled to 0 °C and a catalytic amount of 4-Dimethylaminopyridine (DMAP, 0.08 mmol, 9.8 mg) and Dicyclohexylcarbodiimide (DCC, 1.6 mmol, 329.6 mg) were added sequentially. The reaction was allowed to slowly warm to room temperature and further stirred for 8 hours. Upon completion, the solution was concentrated in vacuo and purified by column chromatography on silica to afford the desired product. The spectral

data is consistent with the literature data.[3]

5. Reaction optimization

Table S1: Screening of different thiols[a]

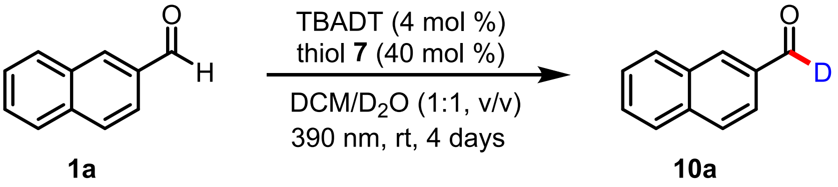

entry	thiol **7**	deuteration (%)[b]
1	**7a**	94
2	**7b**	63
3	**7c**	71
4	**7d**	78
5	**7e**	82
6	**7f**	64
7	**7g**	78
8	**7h**	60

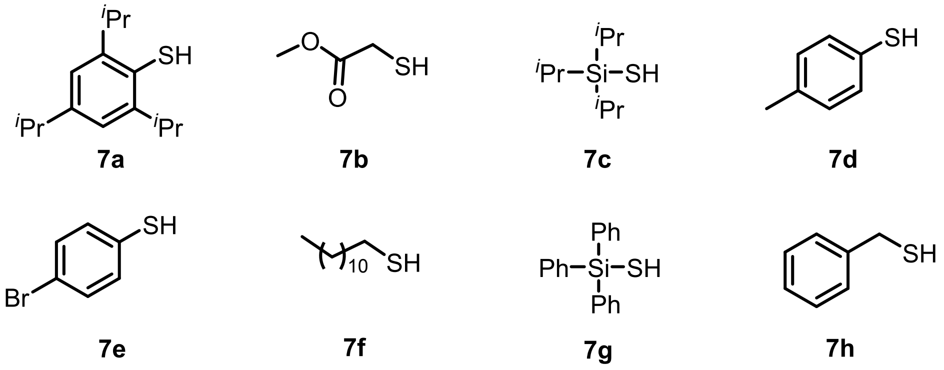

[a]General conditions: **1a** (0.3 mmol), TBADT (0.012 mmol), thiol **7** (0.12 mmol), and DCM/D_2O (1:1, v/v; 3.0 mL) under Ar atmosphere. [b]Deuterium incorporation determined by integration of the residual formyl proton in ^{1}H NMR.

Table S2: Screening of different solvents[a]

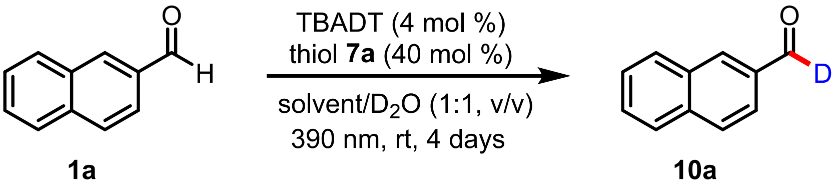

entry	solvent	deuteration (%)[b]
1	DCM	94
2	NMP	13
3	CH_3CN	38
4	$CHCl_3$	62
5	DCE	71

6	EA	21
7	DMSO	14
8	acetone	45

[a]General conditions: **1a** (0.3 mmol), TBADT (0.012 mmol), **7a** (0.12 mmol), and solvent/D_2O (1:1, v/v; 3.0 mL) under Ar atmosphere. [b]Deuterium incorporation determined by integration of the residual formyl proton in ^{1}H NMR.

Table S3: Screening of the amount of TBADT[a]

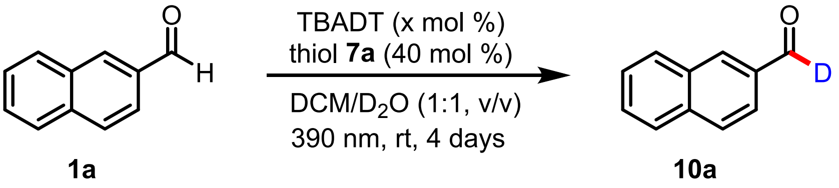

entry	x mol % TBADT	deuteration (%)[b]
1	0	10
2	1	65
3	2	82
4	4	94
5	6	94
6	10	95

[a]General conditions: **1a** (0.3 mmol), TBADT (0.003x mmol), **7a** (0.12 mmol), and DCM/D_2O (1:1, v/v; 3.0 mL) under Ar atmosphere. [b]Deuterium incorporation determined by integration of the residual formyl proton in ^{1}H NMR.

Table S4: Screening of the amount of thiol 7a[a]

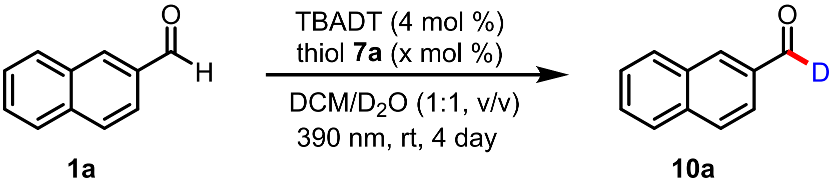

entry	x mol % **7a**	deuteration (%)[b]
1	0	8
2	10	65
3	20	83
4	40	94
5	60	88
6	100	81

[a]General conditions: **1a** (0.3 mmol), TBADT (0.012 mmol), **7a** (0.003x mmol), and DCM/D_2O (1:1, v/v; 3.0 mL) under Ar atmosphere. [b]Deuterium incorporation determined by integration of the residual formyl proton in ^{1}H NMR.

Table S5: Screening of the amount of D_2O[a]

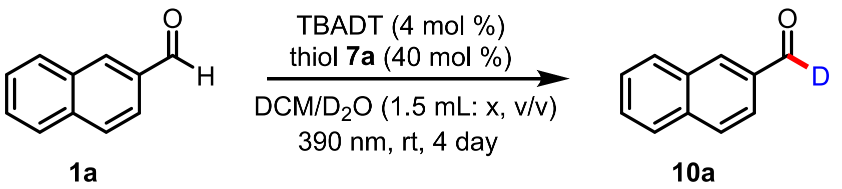

entry	x mL **D_2O**	deuteration (%)[b]
1	1.5	94
2	1.0	87
3	0.5	81

[a]General conditions: **1a** (0.3 mmol), TBADT (0.012 mmol), **7a** (0.12 mmol), and DCM/D_2O (1.5 mL: x mL, v/v) under Ar atmosphere. [b]Deuterium incorporation determined by integration of the residual formyl proton in ^{1}H NMR.

Table S6 Control experiments

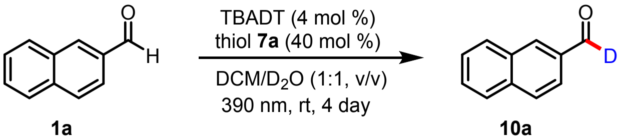

entry	control conditions	deuteration (%)[b]
1	w/o TBADT	<5
2	w/o thiol	<5
3	w/o light	<5
4	470 nm	<5
5	standard conditions, w/all	94

[a]General conditions: **1a** (0.3 mmol), TBADT (0.012 mmol), **7a** (0.12 mmol), and DCM/D_2O (1:1, v/v; 3.0 mL) under Ar atmosphere. [b]Deuterium incorporation determined by integration of the residual formyl proton in ^{1}H NMR.

6. Investigation of the mechamism.

6.1 TEMPO was used as radical scavenger.

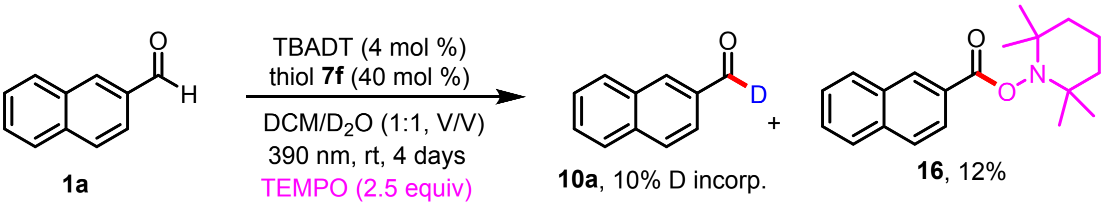

To a 10 mL glass vial was added TBADT (40.8 mg, 0.012 mmol, 4 mol %), aldehyde (0.3 mmol, 1.0 equiv), thiol **7a** (28 mg, 0.12 mmol, 40 mol %), TEMPO (0.75 mmol, 2.5 equiv), and DCM/D_2O (1:1, v/v; 3.0 mL). The reaction mixture was degassed by bubbling with Ar for 15 s with an outlet needle and the vial was sealed with PTFE cap. The mixture was then stirred rapidly and irradiated with a 36 W 390 nm LED (approximately 2 cm away from the light source) at room temperature for 4 days. The reaction mixture was diluted with 10 mL of aqueous 1 M $NaHCO_3$ solution, and extracted with DCM (3 × 20 mL). The combined organic extracts were washed with

brine (40 mL), dried over Na_2SO_4, and concentrated in vacuo. Purification of the crude product by flash chromatography on silica gel afforded **10a** in 10% D incorporation and **16** in 12% yield.

6.2 Benzyl acrylate was used as radical scavenger.

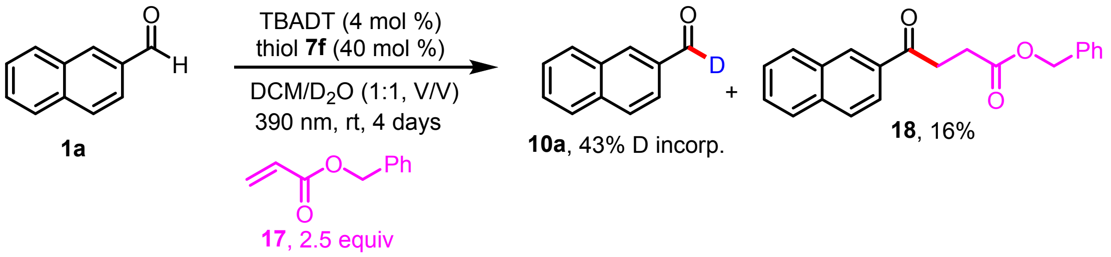

To a 10 mL glass vial was added TBADT (40.8 mg, 0.012 mmol, 4 mol %), aldehyde (0.3 mmol, 1.0 equiv), thiol **7a** (28 mg, 0.12 mmol, 40 mol %), benzyl acrylate **17** (0.75 mmol, 2.5 equiv), and DCM/D_2O (1:1, v/v; 3.0 mL). The reaction mixture was degassed by bubbling with Ar for 15 s with an outlet needle and the vial was sealed with PTFE cap. The mixture was then stirred rapidly and irradiated with a 36 W 390 nm LED (approximately 2 cm away from the light source) at room temperature for 4 days. The reaction mixture was diluted with 10 mL of aqueous 1 M $NaHCO_3$ solution, and extracted with DCM (3 × 20 mL). The combined organic extracts were washed with brine (40 mL), dried over Na_2SO_4, and concentrated in vacuo. Purification of the crude product by flash chromatography on silica gel afforded **10a** in 43% D incorporation, **18** in 16% yield.

6.3 4-pyridinecarboxaldehyde (1qq) was used as the substrate.

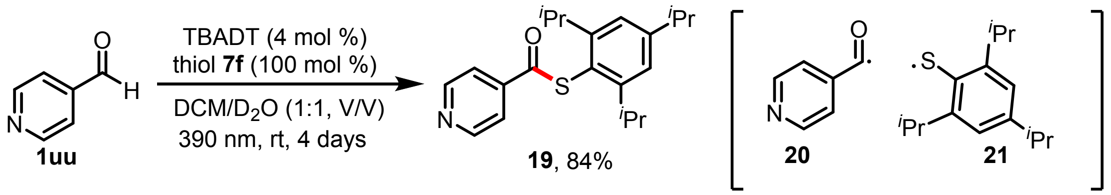

To a 10 mL glass vial was added TBADT (40.8 mg, 0.012 mmol, 4 mol %), 4-pyridinecarboxaldehyde (0.3 mmol, 1.0 equiv), thiol **7a** (70.8 mg, 0.3 mmol, 100 mol %) and DCM/D_2O (1:1, v/v; 3.0 mL). The reaction mixture was degassed by bubbling with Ar for 15 s with an outlet needle and the vial was sealed with PTFE cap. The mixture was then stirred rapidly and irradiated with a 36 W 390 nm LED (approximately 2 cm away from the light source) at room temperature for 4 days. The reaction mixture was diluted with 10 mL of aqueous 1 M $NaHCO_3$ solution, and extracted with DCM (3 × 20 mL). The combined organic extracts were washed with brine (40 mL), dried over Na_2SO_4, and concentrated in vacuo. Purification of the crude product by flash chromatography on silica gel afforded the by-product **19** in 84% yield, which comes from the radical coupling of acyl radical **20** and thiol radical **21**.

6.4 H/D exchange of thiol HAT catalyst 7a with D_2O.

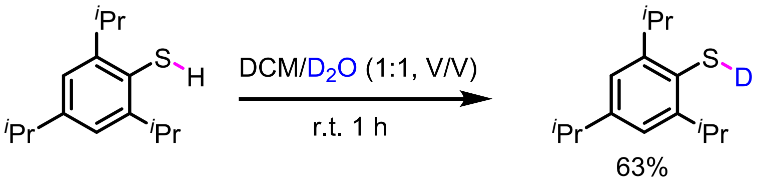

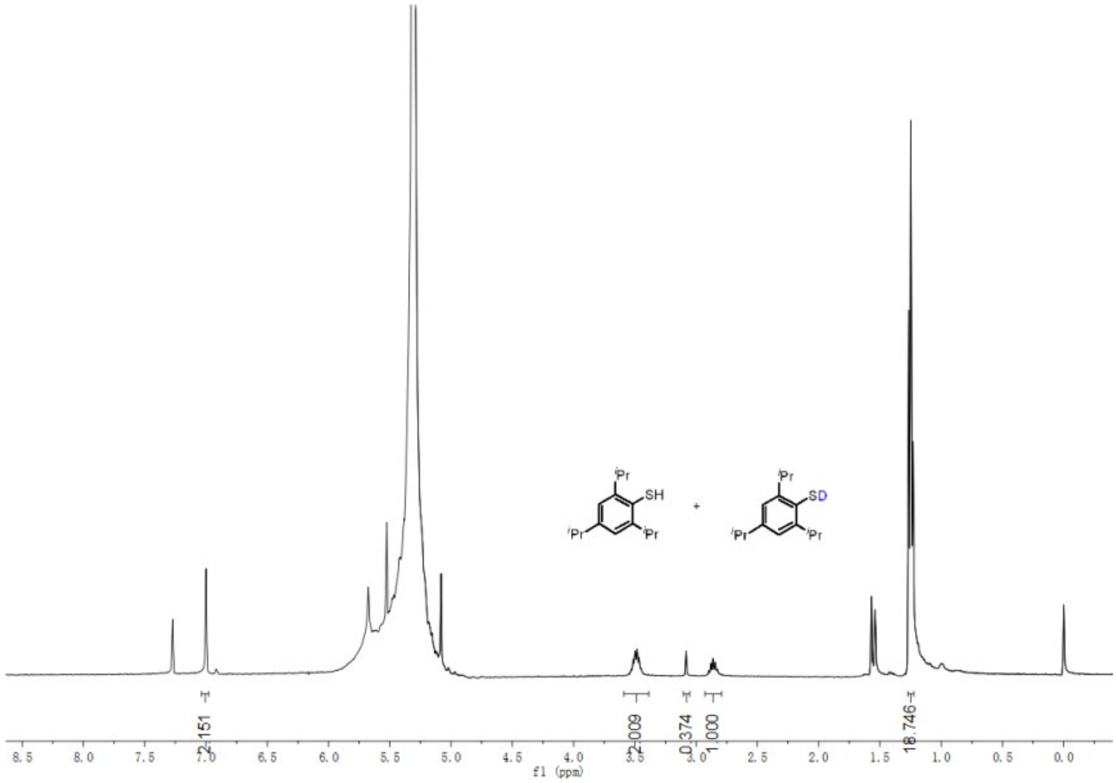

Figure S2 ^{1}H NMR spectrum of thiol HAT catalyst 7a.

The S–H peak from thiol **7a** decreased to 0.37 H in $CDCl_3$, which indicated the hydrogen deuterium exchange of the thiol catalyst **7a** with D_2O.

6.5 Light on/off experiments.

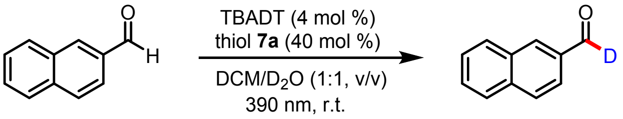

1a **10a**

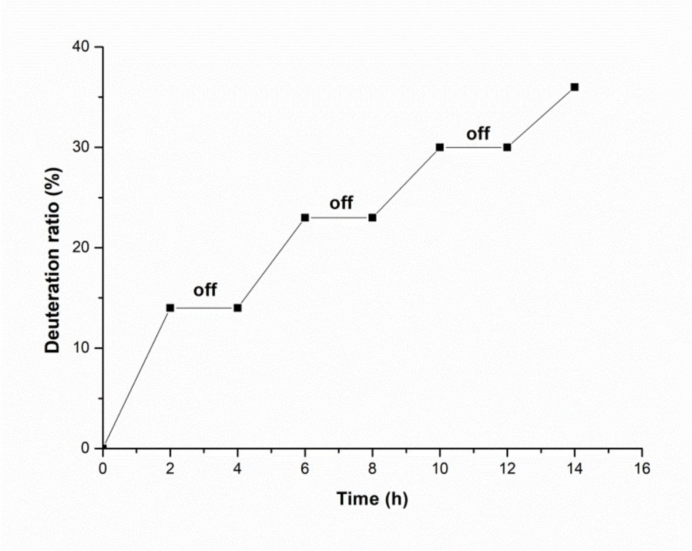

Figure S3 Light on-off experiments for deuteration of 1a.

6.6 Cyclic Voltammogram of Catalyst TBADT.

The potential was calibrated versus an aqueous SCE by the addition of ferrocene as an internal standard taking $E_{(Fc/Fc+)}^0$ = 0.424 V vs SCE.[4] $E_{1/2}^{ox}([W_{10}O_{32}]^{6-}/[W_{10}O_{32}]^{5-})$ = –1.34 V vs SCE.

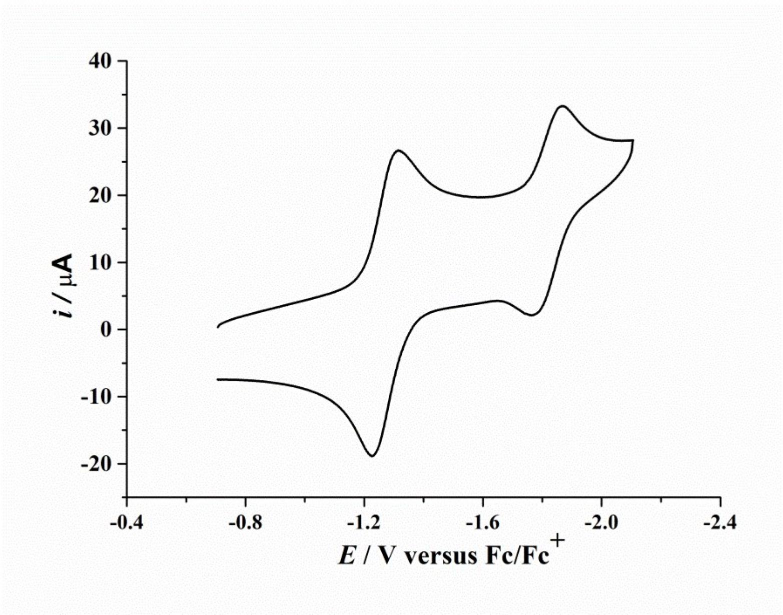

Figure S4. Cyclic voltammograms of TBADT (1.0 mM) in 0.1 M *n*-Bu_4NPF_6/MeCN at a scan rate of 0.1 Vs^{-1}.

7. Experimental Procedures and Product Characterization

7.1 General Procedure for the formyl-selective deuteration of aldehydes.

To a 10 mL glass vial was added TBADT (40.8 mg, 0.012 mmol, 4 mol %), aldehyde (0.3 mmol, 1.0 equiv), thiol **7a** (28 mg, 0.12 mmol, 40 mol %) and DCM/D_2O (1:1, v/v; 3.0 mL). The reaction mixture was degassed by bubbling with Ar for 15 s with an outlet needle and the vial was sealed with PTFE cap. The mixture was then stirred rapidly and irradiated with a 36 W 390 nm LED

(approximately 2 cm away from the light source) at room temperature for 4 days. The reaction mixture was diluted with 10 mL of aqueous 1 M $NaHCO_3$ solution, and extracted with DCM (3 × 20 mL). The combined organic extracts were washed with brine (40 mL), dried over Na_2SO_4, and concentrated in vacuo. Purification of the crude product by flash chromatography on silica gel using the indicated solvent system afforded the desired product.

7.2. Product Characterization

2-naphthaldehyde-*formyl-d₁* (10a).

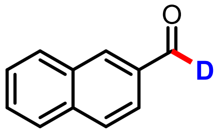

According to the *general procedure*. The spectral Data is consistent with the literature data.[2]
White solid (43.3 mg, 92%). Mp: 86 − 87 °C.
R_f 0.40 (Petroleum ether/EtOAc, 40/1).
D incorporation by 1H NMR: 94%.
^{1}H NMR (400 MHz, $CDCl_3$) δ 10.14 (s, 0.06H), 8.31 (s, 1H), 8.03 – 7.85 (m, 4H), 7.72 – 7.50 (m, 2H). **^{13}C NMR** (100 MHz, $CDCl_3$) δ 192.1 (t, J = 26.5 Hz), 136.5, 134.7, 134.1 (t, J = 3.5 Hz), 132.7, 129.6, 129.2, 129.1, 128.2, 127.2, 122.8.
HRMS (ESI) calcd for $C_{11}H_8DO$ $[M + H]^+$ 158.0711, found 158.0712.

6-methoxy-2-naphthaldehyde-*formyl-d₁* (10b).

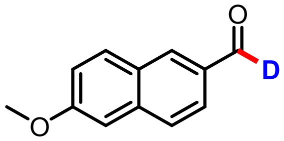

According to the *general procedure*.
White solid (50.5 mg, 90%). Mp: 43 − 44 °C.
R_f 0.40 (Petroleum ether/EtOAc, 40/1).
D incorporation by 1H NMR: 92%.
^{1}H NMR (400 MHz, $CDCl_3$) δ 10.07 (s, 0.08H), 8.21 (s, 1H), 7.95 – 7.82 (m, 2H), 7.77 (d, J = 8.4 Hz, 1H), 7.21 (dd, J = 8.8, 2.4 Hz, 1H), 7.15 (d, J = 2.0 Hz, 1H), 3.93 (s, 3H). **^{13}C NMR** (100 MHz, $CDCl_3$) δ 191.8 (t, J = 26.1 Hz), 160.3, 138.3, 134.3, 132.3 (t, J = 3.3 Hz), 131.2, 128.0, 127.8, 123.7, 120.0, 106.2, 55.5.
HRMS (ESI) calcd for $C_{12}H_{10}DO_2$ $[M + H]^+$ 188.0816, found 188. 188.0817.

1-naphthaldehyde-*formyl-d₁* (10c).

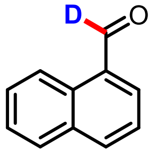

According to the *general procedure*. The spectral Data is consistent with the literature data.[2]
Yellow oil (39.1mg, 83%).
R_f 0.40 (Petroleum ether/EtOAc, 40/1).

D incorporation by 1H NMR: 90%.

^{1}H NMR (400 MHz, $CDCl_3$) δ 10.36 (s, 0.1H), 9.24 (d, J = 8.4 Hz, 1H), 8.06 (d, J = 8.4 Hz, 1H), 7.94 (dd, J = 7.2, 1.0 Hz, 1H), 7.89 (d, J = 8.4 Hz, 1H), 7.74 – 7.63 (m, 1H), 7.63 – 7.49 (m, 2H).

^{13}C NMR (100 MHz, $CDCl_3$) δ 193.4 (t, J = 26.2 Hz), 136.7, 135.4, 133.8, 131.3 (t, J = 3.5 Hz), 130.6, 129.1, 128.6, 127.0, 124.9.

HRMS (ESI) calcd for $C_{11}H_8DO$ $[M + H]^+$ 158.0711, found 158.0712.

4-isopropylbenzaldehyde-*formyl-d₁* (10d).

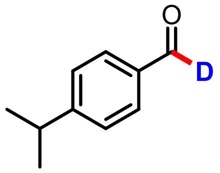

According to the *general procedure*.

Yellow oil (40.7 mg, 91%).

R_f 0.42 (Petroleum ether/EtOAc, 40/1).

D incorporation by 1H NMR: 93%.

^{1}H NMR (400 MHz, $CDCl_3$) δ 9.97 (s, 0.07H), 7.82 (d, J = 8.0 Hz, 2H), 7.39 (d, J = 8.0 Hz, 2H), 2.99 (dt, J = 13.6, 6.8 Hz, 1H), 1.28 (d, J = 6.8 Hz, 6H). **^{13}C NMR** (100 MHz, $CDCl_3$) δ 191.9 (t, J = 26.5 Hz), 156.4, 134.5 (t, J = 3.5 Hz), 130.1, 127.2, 34.6, 23.7.

HRMS (ESI) calcd for $C_{10}H_{12}DO$ $[M + H]^+$ 150.1024, found 150.1024.

4-(*tert*-butyl)benzaldehyde-*formyl-d₁* (10e).

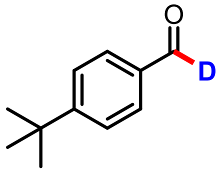

According to the *general procedure*. The spectral Data is consistent with the literature data.[2]

Colorless oil (44.0 mg, 90%).

R_f 0.50 (Petroleum ether/EtOAc, 40/1).

D incorporation by 1H NMR: 93%.

^{1}H NMR (400 MHz, $CDCl_3$) δ 9.98 (s, 0.07H), 7.82 (d, J = 8.4 Hz, 2H), 7.55 (d, J = 8.4 Hz, 2H), 1.36 (s, 9H). **^{13}C NMR** (100 MHz, $CDCl_3$) δ 191.8 (t, J = 27.5 Hz), 158.6, 134.1 (t, J = 3.5 Hz), 129.8, 126.1, 35.5, 31.2.

HRMS (ESI) calcd for $C_{11}H_{14}DO$ $[M + H]^+$ 164.1180, found 164.1181.

[1,1'-biphenyl]-4-carbaldehyde-*formyl-d₁* (10f).

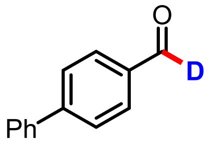

According to the *general procedure*. The spectral Data is consistent with the literature data.[2]

White solid (48.9 mg, 89%). Mp: 84 − 85 °C.

R_f 0.50 (Petroleum ether/EtOAc, 40/1).
D incorporation by 1H NMR: 93%.
^{1}H NMR (400 MHz, $CDCl_3$) δ 10.03 (s, 0.07H), 8.02 – 7.88 (m, 2H), 7.73 (d, J = 7.2 Hz, 2H), 7.62 (d, J = 7.6 Hz, 2H), 7.46 (t, J = 7.6 Hz, 2H), 7.40 (dd, J = 7.6, 6.4 Hz, 1H). **^{13}C NMR** (100 MHz, $CDCl_3$) δ 191.7 (t, J = 26.5 Hz), 147.2, 139.7, 135.1 (t, J = 3.5 Hz), 130.3, 129.1, 128.5, 127.7, 127.4.
HRMS (ESI) calcd for $C_{13}H_{10}DO$ $[M + H]^+$ 184.0867, found 184.0867.

4-fluorobenzaldehyde-*formyl-d$_1$* (10g).

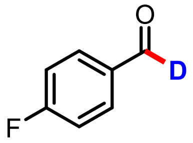

According to the *general procedure*. The spectral Data is consistent with the literature data.[2]
Colorless oil (30.8 mg, 82%).
R_f 0.40 (Petroleum ether/EtOAc, 40/1).
D incorporation by 1H NMR: 96%.
^{1}H NMR (400 MHz, $CDCl_3$) δ 9.98 (s, 0.04H), 7.98 – 7.87 (m, 2H), 7.26 – 7.16 (m, 2H). **^{13}C NMR** (100 MHz, $CDCl_3$) δ 190.3 (t, J = 26.5 Hz), 166.7 (d, J = 256.7 Hz), 133.08 – 132.94 (m), 132.3 (d, J = 9.7 Hz), 116.5 (d, J = 22.5 Hz).
HRMS (ESI) calcd for C_7H_5DFO $[M + H]^+$ 126.0460, found 126.0461.

4-chlorobenzaldehyde-*formyl-d$_1$* (10h).

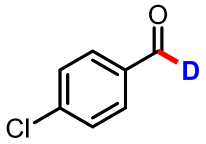

According to the *general procedure*. The spectral Data is consistent with the literature data.[2]
White solid (36.4 mg, 86%). Mp: 44 – 45 °C.
R_f 0.40 (Petroleum ether/EtOAc, 40/1).
D incorporation by 1H NMR: 94%.
^{1}H NMR (400 MHz, $CDCl_3$) δ 9.99 (s, 0.06H), 7.89 – 7.79 (m, 2H), 7.52 (dd, J = 8.4, 2.0 Hz, 2H). **^{13}C NMR** (100 MHz, $CDCl_3$) δ 190.7 (t, J = 26.5 Hz) 141.1, 134.8 (t, J = 3.5 Hz), 131.0, 129.6.
HRMS (ESI) calcd for C_7H_5DClO $[M + H]^+$ 142.0164, found 142.0164.

4-bromobenzaldehyde-*formyl-d$_1$* (10i).

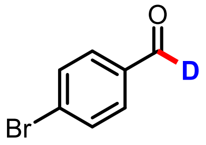

According to the *general procedure*. The spectral Data is consistent with the literature data.[2]
White solid (52.7 mg, 95%). Mp: 76 – 77 °C.
R_f 0.40 (Petroleum ether/EtOAc, 40/1).
D incorporation by 1H NMR: 96%.

^{1}H NMR (400 MHz, $CDCl_3$) δ 9.98 (s, 0.04H), 7.75 (d, J = 8.4 Hz, 2H), 7.68 (d, J = 8.4 Hz, 2H).
^{13}C NMR (100 MHz, $CDCl_3$) δ 190.8 (t, J = 26.5 Hz), 135.1 (t, J = 3.5 Hz), 132.5, 131.1, 129.9.
HRMS (ESI) calcd for C_7H_5DBrO $[M + H]^+$ 185.9659, found 185.9660

3-bromobenzaldehyde-*formyl-d$_1$* (10j).

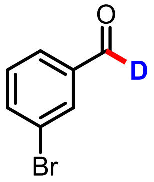

According to the *general procedure*. The spectral Data is consistent with the literature data.[5]
White solid (51.1 mg, 92%). Mp: 80 − 81 °C.
R_f 0.40 (Petroleum ether/EtOAc, 40/1).
D incorporation by 1H NMR: 95%.
^{1}H NMR (400 MHz, $CDCl_3$) δ 9.97 (s, 0.05H), 8.03 (s, 1H), 7.82 (d, J = 7.6 Hz, 1H), 7.77 (d, J = 8.0 Hz, 1H), 7.43 (t, J = 7.6 Hz, 1H). **^{13}C NMR** (100 MHz, $CDCl_3$) δ 190.6 (t, J = 26.5 Hz), 138.0 (t, J = 3.5 Hz), 137.5, 132.5, 130.8, 128.5, 123.5.
HRMS (ESI) calcd for C_7H_5DBrO $[M + H]^+$ 185.9659, found 185.9662

4-iodobenzaldehyde-*formyl-d$_1$* (10k).

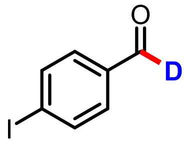

According to the *general procedure*. The spectral Data is consistent with the literature data.[2]
White solid (58.7 mg, 84%). Mp: 98 − 99 °C.
R_f 0.40 (Petroleum ether/EtOAc, 40/1).
D incorporation by 1H NMR: 90%.
^{1}H NMR (400 MHz, $CDCl_3$) δ 9.96 (s, 0.1H), 7.92 (d, J = 8.4 Hz, 2H), 7.60 (d, J = 8.4 Hz, 2H).
^{13}C NMR (100 MHz, $CDCl_3$) δ 191.3 (t, J = 26.5 Hz), 138.5, 135.6 (t, J = 3.5 Hz), 130.9, 103.0.
HRMS (ESI) calcd for C_7H_5DIO $[M + H]^+$ 233.9521, found 233.9518.

4-(trifluoromethyl)benzaldehyde-*formyl-d$_1$* (10l).

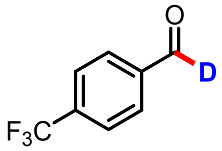

According to the *general procedure*. The spectral Data is consistent with the literature data.[5]
Colorless oil (45.9 mg, 87%).
R_f 0.40 (Petroleum ether/EtOAc, 40/1).
D incorporation by 1H NMR: 97%.
^{1}H NMR (400 MHz, $CDCl_3$) δ 10.11 (s, 0.03H), 8.02 (d, J = 8.0 Hz, 2H), 7.82 (d, J = 8.0 Hz, 2H).
^{13}C NMR (100 MHz, $CDCl_3$) δ 190.9 (t, J = 26.5 Hz), 138.7, 135.7 (q, J = 32.7 Hz), 130.0, 126.2 (q, J = 3.6 Hz), 123.6 (q, J = 271 Hz).

HRMS (ESI) calcd for $C_8H_5DF_3O$ [M + H]$^+$ 176.0428, found 176.0430.

3-(trifluoromethyl)benzaldehyde-*formyl-d₁* (10m).

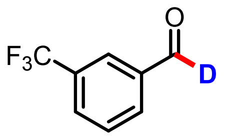

According to the *general procedure*. The spectral Data is consistent with the literature data.[5]
Colorless oil (45.2 mg, 86%).
R_f 0.40 (Petroleum ether/EtOAc, 40/1).
D incorporation by 1H NMR: 95%.
^{1}H NMR (400 MHz, $CDCl_3$) δ 10.09 (s, 0.05H), 8.16 (s, 1H), 8.09 (d, J = 7.6 Hz, 1H), 7.90 (d, J = 7.6 Hz, 1H), 7.71 (t, J = 7.6 Hz, 1H). **^{13}C NMR** (100 MHz, $CDCl_3$) δ 190.6 (t, J = 26.5 Hz), 136.8 (t, J = 3.5 Hz), 132.8, 131.9 (q, J = 33.2 Hz), 130.9 (q, J = 3.6 Hz), 129.9, 126.6 (q, J = 3.8 Hz), 123.6 (q, J = 272.6 Hz).
HRMS (ESI) calcd for $C_8H_5DF_3O$ [M + H]$^+$ 176.0428, found 176.0426.

4-(trifluoromethoxy)benzaldehyde-*formyl-d₁* (10n).

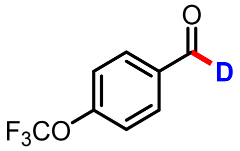

According to the *general procedure*.
Yellow oil (52.7 mg, 92%).
R_f 0.40 (Petroleum ether/EtOAc, 40/1).
D incorporation by 1H NMR: 96%.
^{1}H NMR (400 MHz, $CDCl_3$) δ 10.02 (s, 0.04H), 7.96 (d, J = 8.8 Hz, 2H), 7.37 (d, J = 8.0 Hz, 2H). **^{13}C NMR** (100 MHz, $CDCl_3$) δ 190.4 (t, J = 27.5 Hz), 153.7 (q, J = 3.3Hz), 134.5(t, J = 3.5 Hz), 131.7, 120.9, 120.4 (q, J = 258Hz).
HRMS (ESI) calcd for $C_8H_5DF_3O_2$ [M + H]$^+$ 192.0377, found 192.0379.

3-(trifluoromethoxy)benzaldehyde-*formyl-d₁* (10o).

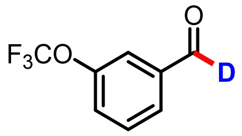

According to the *general procedure*.
Colorless oil (49.9 mg, 87%).
R_f 0.40 (Petroleum ether/EtOAc, 40/1).
D incorporation by 1H NMR: 95%.
^{1}H NMR (400 MHz, $CDCl_3$) δ 10.03 (s, 0.05H), 7.84 (dd, J = 7.6, 1.2 Hz, 1H), 7.74 (s, 1H), 7.60 (t, J = 8.0 Hz, 1H), 7.49 (dd, J = 8.0, 1.0 Hz, 1H). **^{13}C NMR** (100 MHz, $CDCl_3$) δ 190.4 (t, J = 27.5 Hz), 150.0, 138.1 (t, J = 3.5 Hz), 130.8, 128.4, 126.9, 121.1, 120.5 (q, J = 256 Hz).
HRMS (ESI) calcd for $C_8H_5DF_3O_2$ [M + H]$^+$ 192.0377, found 192.0376.

3-formylbenzonitrile-*formyl-d₁* (10p).

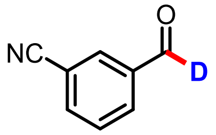

According to the *general procedure*.
Yellow solid (33.7 mg, 85%). Mp: 70 − 71 °C.
R_f 0.40 (Petroleum ether/EtOAc, 40/1).
D incorporation by 1H NMR: 95%.
^{1}H NMR (400 MHz, $CDCl_3$) δ 10.07 (s, 0.05H), 8.19 (s, 1H), 8.15 (d, J = 7.6 Hz, 1H), 7.94 (d, J = 7.6 Hz, 1H), 7.72 (t, J = 7.6 Hz, 1H). **^{13}C NMR** (100 MHz, $CDCl_3$) δ 189.8 (t, J = 29 Hz), 137.3, 136.8 (t, J = 4.0 Hz), 133.4, 133.2, 130.2, 117.7, 113.7.
HRMS (ESI) calcd for C_8H_5DNO $[M + H]^+$ 133.0507, found 133.0507.

methyl 3-formylbenzoate-*formyl-d₁* (10q).

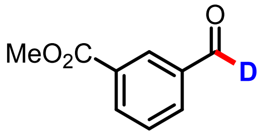

According to the *general procedure*. The spectral Data is consistent with the literature data.[2]
White solid (44.1 mg, 89%). Mp: 49 − 50 °C.
R_f 0.40 (Petroleum ether/EtOAc, 20/1).
D incorporation by 1H NMR: 93%.
^{1}H NMR (400 MHz, $CDCl_3$) δ 10.08 (s, 0.07H), 8.54 (s, 1H), 8.30 (d, J = 7.6 Hz, 1H), 8.10 (d, J = 7.6 Hz, 1H), 7.64 (td, J = 7.6, 2.4 Hz, 1H), 3.97 (s, 3H). **^{13}C NMR** (100 MHz, $CDCl_3$) δ 191.1 (t, J = 27 Hz), 166.0, 136.5 (t, J = 3.5 Hz), 135.2, 133.1, 131.3, 131.3, 129.3, 52.6.
HRMS (ESI) calcd for $C_9H_8DO_3$ $[M + H]^+$ 166.0609, found 166.0608.

4-methoxybenzaldehyde-*formyl-d₁* (10r).

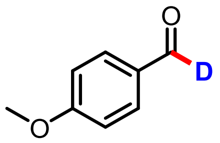

According to the *general procedure*.
Yellow oil (37.0 mg, 90%).
R_f 0.40 (Petroleum ether/EtOAc, 40/1).
D incorporation by 1H NMR: 95%.
^{1}H NMR (400 MHz, $CDCl_3$) δ 9.88 (s, 0.05H), 7.84 (d, J = 8.8 Hz, 2H), 7.00 (d, J = 8.8 Hz, 2H), 3.89 (s, 3H). **^{13}C NMR** (100 MHz, $CDCl_3$) δ 190.6 (t, J = 26 Hz), 164.7, 132.0, 129.9 (t, J = 3.5 Hz), 114.4, 55.6.
HRMS (ESI) calcd for $C_8H_8DO_2$ $[M + H]^+$ 138.0660, found 138.0659.

4-(benzyloxy)benzaldehyde-*formyl-d₁* (10s).

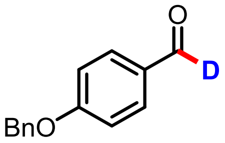

According to the *general procedure*. The spectral Data is consistent with the literature data.[5]
White solid (59.4mg, 93%). Mp: 96 − 97 °C.
R_f 0.40 (Petroleum ether/EtOAc, 20/1).
D incorporation by 1H NMR: 95%.
^{1}H NMR (400 MHz, $CDCl_3$) δ 9.86 (s, 0.05H), 7.82 (d, J = 8.8 Hz, 2H), 7.38 (dt, J = 13.6, 7.6 Hz, 5H), 7.06 (d, J = 8.8 Hz, 2H), 5.12 (s, 2H). **^{13}C NMR** (100 MHz, $CDCl_3$) δ 190.5 (t, J = 26 Hz), 163.8, 136.0, 132.0, 130.1 (t, J = 3.0 Hz), 128.8, 128.4, 127.6, 115.2, 70.3.
HRMS (ESI) calcd for $C_{14}H_{12}DO_2$ $[M + H]^+$ 214.0973, found 214.0971.

3-formylphenyl 4-methylbenzenesulfonate-*formyl-d₁* (10t).

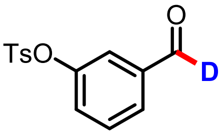

According to the *general procedure*.
White solid (70.6 mg, 85%). Mp: 63 − 64 °C.
R_f 0.40 (Petroleum ether/EtOAc, 5/1).
D incorporation by 1H NMR: 94%.
^{1}H NMR (400 MHz, $CDCl_3$) δ 9.93 (s, 0.06H), 7.79 (d, J = 7.2 Hz, 1H), 7.76 – 7.67 (m, 2H), 7.57 – 7.45 (m, 2H), 7.31 (dd, J = 21.2, 7.6 Hz, 3H), 2.45 (s, 3H). **^{13}C NMR** (100 MHz, $CDCl_3$) δ 190.4 (t, J = 26.5 Hz), 150.2, 145.9, 137.8 (t, J = 3.0 Hz), 131.9, 130.5, 130.0, 128.5, 128.4, 128.3, 123.0, 21.7.
HRMS (ESI) calcd for $C_{14}H_{12}DO_4S$ $[M + H]^+$ 278.0592, found 278.0590.

3-methoxybenzaldehyde-*formyl-d_1* (10u).

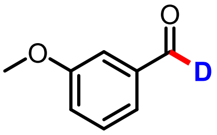

According to the *general procedure*.
Colorless oil (34.9 mg, 85%).
R_f 0.40 (Petroleum ether/EtOAc, 40/1).
D incorporation by 1H NMR: 92%.
^{1}H NMR (400 MHz, $CDCl_3$) δ 10.00 (s, 0.08H), 7.57 – 7.36 (m, 3H), 7.25 – 7.14 (m, 1H), 3.88 (d, J = 10.4 Hz, 3H). **^{13}C NMR** (100 MHz, $CDCl_3$) δ 191.9 (t, J = 26.5 Hz), 160.3, 137.8(t, J = 3.5 Hz), 130.1, 123.6, 121.6, 112.1, 55.6.
HRMS (ESI) calcd for $C_8H_8DO_2$ $[M + H]^+$ 138.0660, found 138.0659.

***tert*-butyl (3-formylphenyl)carbamate-*formyl-d_1* (10v).**

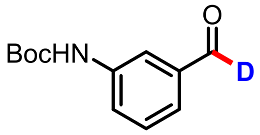

According to the *general procedure*. The spectral Data is consistent with the literature data.[2]
White solid (55.3 mg, 83%). Mp: 88 − 89 °C.
R_f 0.40 (Petroleum ether/EtOAc, 10/1).
D incorporation by 1H NMR: 88%.
^{1}H NMR (400 MHz, $CDCl_3$) δ 9.97 (s, 0.12H), 7.96 (s, 1H), 7.66 (d, J = 7.6 Hz, 1H), 7.59 – 7.51 (m, 1H), 7.44 (t, J = 7.6 Hz, 1H), 7.03 (s, 1H), 1.53 (s, 9H). **^{13}C NMR** (100 MHz, $CDCl_3$) δ 192.1 (t, J = 26.5 Hz), 152.8, 139.5, 137.1 (t, J = 3.5 Hz), 129.7, 124.3, 124.1, 119.4, 81.1, 28.3.
HRMS (ESI) calcd for $C_{12}H_{15}DNO_3$ [M + H]$^+$ 223.1187, found 223.1186.

4-(4,4,5,5-tetramethyl-1,3,2-dioxaborolan-2-yl)benzaldehyde-*formyl-d$_1$* (10w).

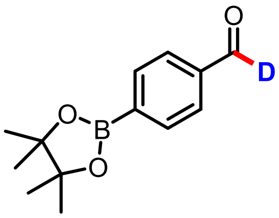

According to the *general procedure*. The spectral Data is consistent with the literature data.[2]
Colorless oil (61.5 mg, 88%).
R_f 0.40 (Petroleum ether/EtOAc, 20/1).
D incorporation by 1H NMR: 91%.
^{1}H NMR (400 MHz, $CDCl_3$) δ 10.05 (s, 0.09H), 7.97 (d, J = 8.0 Hz, 2H), 7.87 (d, J = 8.0 Hz, 2H), 1.36 (s, 12H). **^{13}C NMR** (100 MHz, $CDCl_3$) δ 192.3 (t, J = 26.5 Hz), 138.1, 138.0 (t, J = 3.5 Hz), 135.2, 128.7, 84.3, 24.9.
HRMS (ESI) calcd for $C_{13}H_{17}DBO_3$ [M + H]$^+$ 234.1406, found 234.1402.

3-(4,4,5,5-tetramethyl-1,3,2-dioxaborolan-2-yl)benzaldehyde-*formyl-d$_1$* (10x).

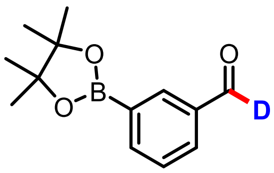

According to the *general procedure*.
Colorless oil (58.0 mg, 83%).
R_f 0.40 (Petroleum ether/EtOAc, 20/1).
D incorporation by 1H NMR: 87%.
^{1}H NMR (400 MHz, $CDCl_3$) δ 10.05 (s, 0.13H), 8.31 (s, 1H), 8.06 (d, J = 7.2 Hz, 1H), 8.03 – 7.94 (m, 1H), 7.53 (t, J = 7.6 Hz, 1H), 1.37 (s, 12H). **^{13}C NMR** (100 MHz, $CDCl_3$) δ 192.1 (t, J = 25.5 Hz), 140.8, 137.3, 135.8, 135.7 (t, J = 3.5 Hz), 131.4, 128.5, 84.4, 24.9.
HRMS (ESI) calcd for $C_{13}H_{17}DBO_3$ [M + H]$^+$ 234.1406, found 234.1403.

4-((trimethylsilyl)ethynyl)benzaldehyde-*formyl-d₁* (10y).

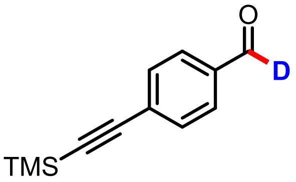

According to the *general procedure*.

Colorless oil (52.4 mg, 86%).

R_f 0.40 (Petroleum ether/EtOAc, 40/1).

D incorporation by 1H NMR: 91%.

^{1}H NMR (400 MHz, $CDCl_3$) δ 9.96 (s, 0.09H), 7.78 (d, J = 8.4 Hz, 2H), 7.57 (d, J = 8.4 Hz, 2H), 0.24 (s, 9H). **^{13}C NMR** (100 MHz, $CDCl_3$) δ 191.2 (t, J = 26.5 Hz), 135.6 (t, J = 3.5 Hz), 132.6, 129.5, 129.4, 103.9, 99.1, -0.1.

HRMS (ESI) calcd for $C_{12}H_{14}DOSi$ $[M + H]^+$ 204.0949, found 204.0949.

3-(allyloxy)benzaldehyde-*formyl-d₁* (10z).

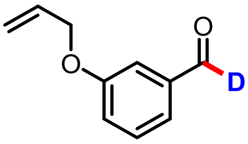

According to the *general procedure*. The spectral Data is consistent with the literature data.[2]

Colorless oil (35.7 mg, 73%).

R_f 0.40 (Petroleum ether/EtOAc, 40/1).

D incorporation by 1H NMR: 90%.

^{1}H NMR (400 MHz, $CDCl_3$) δ 9.97 (s, 0.1H), 7.51 – 7.37 (m, 3H), 7.20 (dt, J = 6.8, 2.4 Hz, 1H), 6.06 (ddd, J = 22.4, 10.4, 5.2 Hz, 1H), 5.44 (dd, J = 17.2, 1.2 Hz, 1H), 5.32 (dd, J = 10.4, 1.2 Hz, 1H), 4.60 (d, J = 5.2 Hz, 2H). **^{13}C NMR** (100 MHz, $CDCl_3$) δ 191.9 (t, J = 26.5 Hz), 159.2, 137.8 (t, J = 3.5 Hz), 132.7, 130.2, 123.7, 122.3, 118.2, 113.2, 69.1.

HRMS (ESI) calcd for $C_{10}H_{10}DO_2$ $[M + H]^+$ 164.0816, found 164.0816.

3,4-dimethylbenzaldehyde-*formyl-d₁* (10aa).

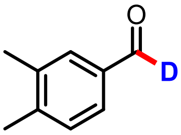

According to the *general procedure*.

Colorless oil (32.4 mg, 80%).

R_f 0.40 (Petroleum ether/EtOAc, 40/1).

D incorporation by 1H NMR: 95%.

^{1}H NMR (400 MHz, $CDCl_3$) δ 9.93 (s, 0.05H), 7.78 – 7.56 (m, 2H), 7.28 (d, J = 7.6 Hz, 1H), 2.63 – 2.27 (m, 6H). **^{13}C NMR** (100 MHz, $CDCl_3$) δ 192.1 (t, J = 26.5 Hz), 144.4, 137.6, 134.6 (t, J = 3.5 Hz), 130.6, 130.3, 127.8, 20.4, 19.7.

HRMS (ESI) calcd for $C_9H_{10}DO$ $[M + H]^+$ 136.0867, found 136.0867.

4-fluoro-3-methylbenzaldehyde-*formyl-d₁* (10bb).

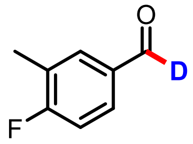

According to the *general procedure*.
Colorless oil (37.1 mg, 89%).
R_f 0.40 (Petroleum ether/EtOAc, 40/1).
D incorporation by 1H NMR: 93%.
^{1}H NMR (400 MHz, $CDCl_3$) δ 9.93 (s, 0.07H), 7.73 (ddd, J = 8.4, 7.2, 4.8 Hz, 2H), 7.16 (t, J = 8.8 Hz, 1H), 2.35 (d, J = 2.0 Hz, 3H). **^{13}C NMR** (100 MHz, $CDCl_3$) δ 190.7 (t, J = 26.5 Hz), 165.3 (d, J = 255.6 Hz), 133.3 (d, J = 7.0 Hz), 132.8 (t, J = 3.5 Hz), 129.9 (d, J = 9.7 Hz), 126.3 (d, J = 18.1 Hz), 116.0 (d, J = 23.4 Hz), 14.6 (d, J = 3.5 Hz).
HRMS (ESI) calcd for C_8H_7DFO $[M + H]^+$ 140.0616, found 140.0616.

nicotinaldehyde-*formyl-d₁* (10cc).

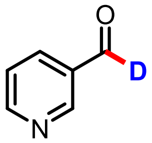

According to the *general procedure*.
Colorless oil (26.6 mg, 82%).
R_f 0.40 (Petroleum ether/EtOAc, 4/1).
D incorporation by 1H NMR: 94%.
^{1}H NMR (400 MHz, $CDCl_3$) δ 10.14 (s, 0.06H), 9.10 (s, 1H), 8.98 – 8.71 (m, 1H), 8.31 – 8.12 (m, 1H), 7.51 (dd, J = 7.6, 4.8 Hz, 1H). **^{13}C NMR** (100 MHz, $CDCl_3$) δ 154.9, 152.3, 135.9, 131.5 (t, J = 3.5 Hz), 124.2.
HRMS (ESI) calcd for C_6H_5DNO $[M + H]^+$ 109.0507, found 109.0509.

quinoline-6-carbaldehyde-*formyl-d₁* (10dd).

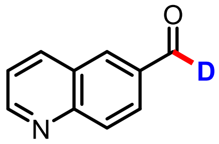

According to the *general procedure*. The spectral Data is consistent with the literature data.[2]
White solid (39.3 mg, 83%). Mp: 68 − 69 °C.
R_f 0.40 (Petroleum ether/EtOAc, 4/1).
D incorporation by 1H NMR: 90%.
^{1}H NMR (400 MHz, $CDCl_3$) δ 10.20 (s, 0.1H), 9.05 (dd, J = 4.4, 1.6 Hz, 1H), 8.39 – 8.30 (m, 2H), 8.21 (s, 2H), 7.53 (dd, J = 8.4, 4.4 Hz, 1H). **^{13}C NMR** (100 MHz, $CDCl_3$) δ 191.3 (t, J = 27 Hz), 153.2, 150.9, 137.5, 134.3 (t, J = 3.5 Hz), 133.7, 130.9, 127.8, 126.8, 122.3.
HRMS (ESI) calcd for $C_{10}H_7DNO$ $[M + H]^+$ 159.0663, found 159.0663.

1-tosyl-1*H*-indole-5-carbaldehyde-*formyl-d₁* (10ee).

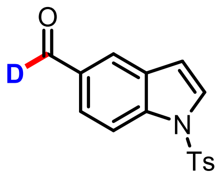

According to the *general procedure*.
White solid (78.3 mg, 87%). Mp: 120 − 121 °C.
R_f 0.40 (Petroleum ether/EtOAc, 5/1).
D incorporation by 1H NMR: 91%.
^{1}H NMR (400 MHz, $CDCl_3$) δ 10.02 (s, 0.09H), 8.11 (d, J = 8.8 Hz, 1H), 8.06 (s, 1H), 7.85 (dd, J = 8.8, 1.2 Hz, 1H), 7.79 (d, J = 8.4 Hz, 2H), 7.68 (d, J = 3.6 Hz, 1H), 7.24 (d, J = 8.4 Hz, 2H), 6.77 (d, J = 3.6 Hz, 1H), 2.33 (s, 3H). **^{13}C NMR** (100 MHz, $CDCl_3$) δ 191.6 (t, J = 27 Hz), 145.7, 138.1, 134.9, 132.2 (t, J = 3.5 Hz), 130.9, 130.2, 128.1, 126.9, 125.2, 124.9, 114.0, 109.4, 21.6.
HRMS (ESI) calcd for $C_{16}H_{13}DNO_3S$ $[M + H]^+$ 301.0752, found 301.0750.

thiazole-4-carbaldehyde-*formyl-d₁* (10ff).

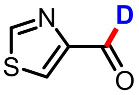

According to the *general procedure*.
White solid (29.4 mg, 86%). Mp: 49 − 50 °C.
R_f 0.40 (Petroleum ether/EtOAc, 4/1).
D incorporation by 1H NMR: 93%.
^{1}H NMR (400 MHz, $CDCl_3$) δ 10.14 (s, 0.07H), 8.94 (d, J = 2.0 Hz, 1H), 8.29 (d, J = 2.0 Hz, 1H). **^{13}C NMR** (100 MHz, $CDCl_3$) δ 184.5 (t, J = 28 Hz), 155.6 (t, J = 3.0 Hz), 154.1, 126.9.
HRMS (ESI) calcd for C_4H_3DNOS $[M + H]^+$ 115.0071, found 115.0073.

5-methylthiophene-2-carbaldehyde-*formyl-d₁* (10gg).

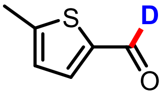

According to the *general procedure*.
Yellow oil (30.5 mg, 80%).
R_f 0.40 (Petroleum ether/EtOAc, 40/1).
D incorporation by 1H NMR: 92%.
^{1}H NMR (400 MHz, $CDCl_3$) δ 9.80 (s, 0.08H), 7.60 (d, J = 3.6 Hz, 1H), 6.89 (d, J = 3.6 Hz, 1H), 2.57 (s, 3H). **^{13}C NMR** (100 MHz, $CDCl_3$) δ 182.4 (t, J = 25.5 Hz), 151.7, 142.0 (t, J = 3.0 Hz), 137.4, 127.2, 16.3.
HRMS (ESI) calcd for C_6H_6DOS $[M + H]^+$ 128.0275, found 128.0275.

3-phenylpropanal-*formyl-d₁* (10hh).

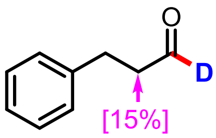

According to the *general procedure*. The spectral Data is consistent with the literature data.[5]
Colorless oil (36.7 mg, 90%).
R_f 0.40 (Petroleum ether/EtOAc, 40/1).
D incorporation by 1H NMR: 95%.
^{1}H NMR (400 MHz, $CDCl_3$) δ 9.81 (s, 0.05H), 7.29 (t, J = 7.6 Hz, 2H), 7.20 (t, J = 7.6 Hz, 3H), 3.03 – 2.89 (m, 2H), 2.77 (t, J = 7.6 Hz, 1.71H). **^{13}C NMR** (100 MHz, $CDCl_3$) δ 201.4 (t, J = 26 Hz), 140.4, 128.7, 128.4, 126.4, 45.2 (t, J = 3.5 Hz), 28.2.
HRMS (ESI) calcd for $C_9H_{10}DO$ $[M + H]^+$ 136.0867, found 136.0867.

3-(4-isopropylphenyl)-2-methylpropanal-*formyl-d₁* (10ii).

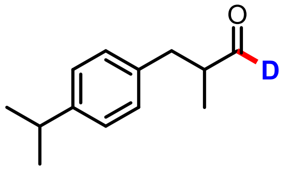

According to the *general procedure*.
Colorless oil (53.9 mg, 94%).
R_f 0.40 (Petroleum ether/EtOAc, 40/1).
D incorporation by 1H NMR: 94%.
^{1}H NMR (400 MHz, $CDCl_3$) δ 9.71 (s, 0.06H), 7.15 (d, J = 8.0 Hz, 2H), 7.09 (d, J = 8.0 Hz, 2H), 3.05 (dd, J = 13.2, 6.0 Hz, 1H), 2.88 (dt, J = 13.6, 6.8 Hz, 1H), 2.74 – 2.51 (m, 2H), 1.23 (d, J = 6.8 Hz, 6H), 1.08 (d, J = 6.8 Hz, 3H). **^{13}C NMR** (100 MHz, $CDCl_3$) δ 204.4 (t, J = 25.5 Hz), 147.1, 136.2, 129.0, 126.6, 48.0 (t, J = 3.5 Hz), 36.3, 33.8, 24.1, 13.3.
HRMS (ESI) calcd for $C_{13}H_{18}DO$ $[M + H]^+$ 192.1493, found 192.1494.

3-(benzo[*d*][1,3]dioxol-5-yl)-2-methylpropanal-*formyl-d₁* (10jj).

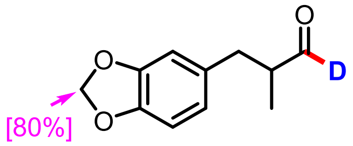

According to the *general procedure*.
Colorless oil (48.9 mg, 84%).
R_f 0.40 (Petroleum ether/EtOAc, 20/1).
D incorporation by 1H NMR: 90%.
^{1}H NMR (400 MHz, $CDCl_3$) δ 9.70 (d, J = 1.2 Hz, 0.1H), 6.73 (d, J = 8.0 Hz, 1H), 6.65 (d, J = 1.2 Hz, 1H), 6.61 (dd, J = 8.0, 1.2 Hz, 1H), 5.95 – 5.87 (m, 0.39H), 3.08 – 2.87 (m, 1H), 2.69 – 2.42 (m, 2H), 1.08 (d, J = 6.8 Hz, 3H). **^{13}C NMR** (100 MHz, $CDCl_3$) δ 204.2 (t, J = 25.9 Hz), 147.8, 146.2, 132.6, 122.0, 109.4, 108.3, 100.7 (t, J = 26.6 Hz), 48.1 (t, J = 3.4 Hz), 36.5, 13.2.
HRMS (ESI) calcd for $C_{11}H_{11}D_2O_3$ $[M + H]^+$ 195.0985, found 195.0988.

4-phenylbutanal-*formyl-d₁* (10kk).

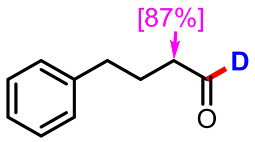

According to the *general procedure*.
Colorless oil (37.1 mg, 82%).
R_f 0.60 (Petroleum ether/EtOAc, 20/1).
D incorporation by 1H NMR: 93%.
^{1}H NMR (400 MHz, $CDCl_3$) δ 9.75 (d, J = 5.6 Hz, 0.07H), 7.34 – 7.25 (m, 2H), 7.25 – 7.14 (m, 3H), 2.86 – 2.55 (m, 2H), 2.42 (s, 0.26H), 1.95 (d, J = 5.2 Hz, 2H). **^{13}C NMR** (100 MHz, $CDCl_3$) δ 202.5 (t, J = 24.8 Hz), 141.2, 128.5, 126.1, 42.4 (t, J = 26.5 Hz), 35.0, 23.5.
HRMS (ESI) calcd for $C_{10}H_{10}D_3O$ [M + H]$^+$ 152.1149, found 152.1150.

2,2-diphenylacetaldehyde-*formyl-d$_1$* (10ll).

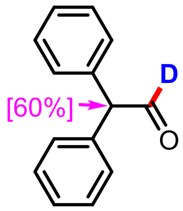

According to the *general procedure*.
Colorless oil (45.1 mg, 76%).
R_f 0.65 (Petroleum ether/EtOAc, 20/1).
D incorporation by 1H NMR: 98%.
^{1}H NMR (400 MHz, $CDCl_3$) δ 9.88 (s, 0.02H), 7.81 (d, J = 7.6 Hz, 2H), 7.59 (t, J = 7.6 Hz, 1H), 7.49 (t, J = 7.6 Hz, 2H), 7.17 (d, J = 7.6 Hz, 2H), 7.10 (t, J = 7.2 Hz, 2H), 7.01 (t, J = 7.2 Hz, 1H), 4.77 (s, 0.4H). **^{13}C NMR** (100 MHz, $CDCl_3$) δ 196.9 (t, J = 23.8 Hz), 143.6, 137.8, 132.6, 130.2, 128.7, 128.4, 128.3, 126.0, 56.5.
HRMS (ESI) calcd for $C_{14}H_{11}D_2O$ [M + H]$^+$ 199.1086, found 199.1088.

3-(1,3-dioxoisoindolin-2-yl)propanal-*formyl-d$_1$* (10mm).

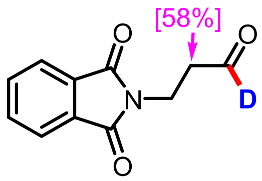

According to the *general procedure*.
White solid (57.2 mg, 93%). Mp: 120 − 121 °C.
R_f 0.40 (Petroleum ether/EtOAc, 3/1).
D incorporation by 1H NMR: 92%.
^{1}H NMR (400 MHz, $CDCl_3$) δ 9.83 (s, 0.08H), 7.95 – 7.80 (m, 2H), 7.80 – 7.67 (m, 2H), 4.09 – 3.96 (m, 2H), 2.93 – 2.77 (m, 0.83H). **^{13}C NMR** (101 MHz, $CDCl_3$) δ 199.6 (t, J = 24.2 Hz), 168.1, 134.2, 132.0, 123.5, 42.00 (m), 31.7 (t, J = 5.5 Hz).
HRMS (ESI) calcd for $C_{11}H_8D_2NO_3$ [M + H]$^+$ 206.0781, found 206.0776.

(2*S*,5*R*)-2-isopropyl-5-methylcyclohexyl 3-formylbenzoate-*formyl-d₁* (10nn).

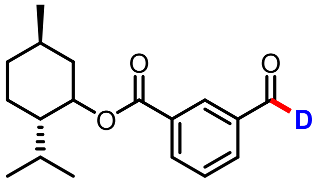

According to the *general procedure*. The spectral Data is consistent with the literature data.[2]
Yellow oil (76.3 mg, 88%).
R_f 0.40 (Petroleum ether/EtOAc, 40/1).
D incorporation by 1H NMR: 91%.
^{1}H NMR (400 MHz, $CDCl_3$) δ 10.10 (s, 0.09H), 8.53 (s, 1H), 8.32 (d, J = 7.6 Hz, 1H), 8.09 (d, J = 7.6 Hz, 1H), 7.64 (t, J = 7.6 Hz, 1H), 5.00 (td, J = 10.8, 4.4 Hz, 1H), 2.13 (d, J = 12.0 Hz, 1H), 1.95 (dtd, J = 13.6, 6.8, 2.4 Hz, 1H), 1.80 – 1.70 (m, 2H), 1.65 – 1.51 (m, 2H), 1.14 (dd, J = 23.2, 11.6 Hz, 2H), 1.03 – 0.88 (m, 7H), 0.81 (d, J = 6.8 Hz, 3H). **^{13}C NMR** (100 MHz, $CDCl_3$) δ 191.3 (t, J = 28 Hz), 165.1, 136.6 (t, J = 3.5 Hz), 135.3, 133.0, 132.0, 131.3, 129.3, 75.6, 47.3, 41.0, 34.3, 31.6, 26.6, 23.6, 22.1, 20.9, 16.5.
HRMS (ESI) calcd for $C_{18}H_{24}DO_3$ $[M + H]^+$ 290.1861, found 290.1862.

(3*S*,8*S*,9*S*,10*R*,13*S*,14*S*,17*S*)-17-acetyl-10,13-dimethyl-2,3,4,7,8,9,10,11,12,13,14,15,16,17-tetradecahydro-1*H*-cyclopenta[*a*]phenanthren-3-yl 3-formylbenzoate-*formyl-d₁* (10oo).

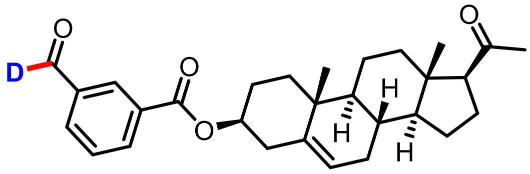

According to the *general procedure*. The spectral Data is consistent with the literature data.[2]
White solid (114.5 mg, 85%). Mp: 158 − 159 °C.
R_f 0.40 (Petroleum ether/EtOAc, 20/1).
D incorporation by 1H NMR: 95%.
^{1}H NMR (400 MHz, $CDCl_3$) δ 10.09 (s, 0.05H), 8.53 (s, 1H), 8.31 (d, J = 7.6 Hz, 1H), 8.09 (d, J = 7.6 Hz, 1H), 7.64 (t, J = 7.6 Hz, 1H), 5.43 (d, J = 4.0 Hz, 1H), 4.98 – 4.83 (m, 1H), 2.61 – 2.44 (m, 3H), 2.24 – 2.11 (m, 4H), 2.10 – 1.90 (m, 4H), 1.81 (dd, J = 18.8, 7.6 Hz, 1H), 1.75 – 1.60 (m, 3H), 1.51 (dd, J = 16.0, 6.8 Hz, 3H), 1.33 – 1.13 (m, 4H), 1.13 – 1.00 (m, 4H), 0.65 (s, 3H). **^{13}C NMR** (100 MHz, $CDCl_3$) δ 209.6, 191.2 (t, J = 28 Hz), 164.9, 139.6, 136.5, 135.2, 133.1, 131.9, 131.1, 129.3, 122.7, 75.1, 63.7, 56.9, 56.84, 49.9, 44.0, 38.8, 38.2, 37.1, 36.7, 31.8, 31.6, 27.9, 24.5, 24.4, 22.9, 21.1, 19.4, 13.3.
HRMS (ESI) calcd for $C_{29}H_{36}DO_4$ $[M + H]^+$ 450.2749, found 450.2748.

3-formylphenyl 2-(4-isobutylphenyl)propanoate-*formyl-d₁* (10pp).

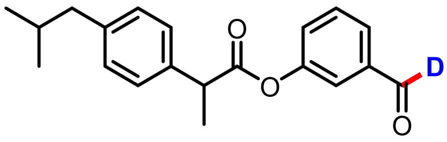

According to the *general procedure*.
Colorless oil (74.6 mg, 80%).
R_f 0.40 (Petroleum ether/EtOAc, 20/1).
D incorporation by 1H NMR: 93%.
^{1}H NMR (400 MHz, $CDCl_3$) δ 9.95 (s, 0.07H), 7.71 (d, J = 7.6 Hz, 1H), 7.51 (dt, J = 15.6, 4.8 Hz, 2H), 7.35 – 7.23 (m, 3H), 7.16 (t, J = 6.4 Hz, 2H), 3.96 (q, J = 7.2 Hz, 1H), 2.47 (d, J = 7.2 Hz, 2H), 1.87 (tt, J = 13.2, 6.8 Hz, 1H), 1.61 (d, J = 7.2 Hz, 3H), 0.91 (d, J = 6.8 Hz, 6H). **^{13}C NMR** (100 MHz, $CDCl_3$) δ 191.0 (t, J = 27.5 Hz), 173.0, 151.5, 141.1, 137.6 (t, J = 3.5 Hz), 136.9, 130.1, 129.7, 127.8, 127.4, 127.3, 122.2, 45.3, 45.1, 30.3, 22.5, 18.6.
HRMS (ESI) calcd for $C_{20}H_{22}DO_3$ $[M + H]^+$ 312.1704, found 312.1706.

3-formylphenyl 4-([1,1'-biphenyl]-4-yl)-4-oxobutanoate-*formyl-d₁* (10qq).

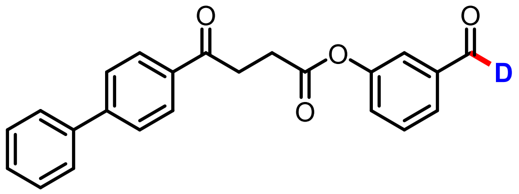

According to the *general procedure*.
White solid (78.6 mg, 73%). Mp: 89 – 90 °C.
R_f 0.40 (Petroleum ether/EtOAc, 4/1).
D incorporation by 1H NMR: 93%.
^{1}H NMR (400 MHz, $CDCl_3$) δ 10.00 (s, 0.07H), 8.09 (d, J = 8.0 Hz, 2H), 7.76 (d, J = 7.6 Hz, 1H), 7.71 (d, J = 8.0 Hz, 2H), 7.68 – 7.59 (m, 3H), 7.55 (t, J = 7.6 Hz, 1H), 7.47 (t, J = 7.6 Hz, 2H), 7.41 (t, J = 7.6 Hz, 2H), 3.48 (t, J = 6.4 Hz, 2H), 3.06 (t, J = 6.4 Hz, 2H). **^{13}C NMR** (100 MHz, $CDCl_3$) δ 197.4, 191.0 (t, J = 27.5 Hz), 171.5, 151.4, 146.2, 139.9, 137.8 (t, J = 3.5 Hz), 135.1, 130.2, 129.1, 128.8, 128.4, 127.9, 127.5, 127.4, 127.3, 122.6, 33.6, 28.6.
HRMS (ESI) calcd for $C_{23}H_{18}DO_4$ $[M + H]^+$ 360.1341, found 360.1342.

6-(3-((3r,5r,7r)-adamantan-1-yl)-4-methoxyphenyl)-2-naphthaldehyde-*formyl-d₁* (10rr).

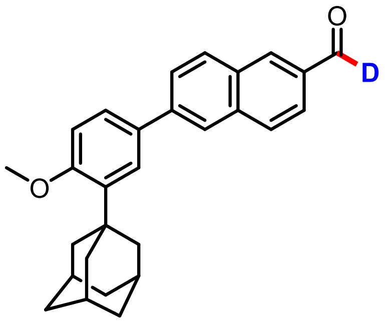

According to the *general procedure*. The spectral Data is consistent with the literature data.[2]
White solid (106.0 mg, 89%). Mp: 236 – 237 °C.
R_f 0.40 (Petroleum ether/EtOAc, 20/1).
D incorporation by 1H NMR: 93%.
^{1}H NMR (400 MHz, $CDCl_3$) δ 10.16 (s, 0.07H), 8.35 (s, 1H), 8.04 (dd, J = 4.8, 3.2 Hz, 2H), 7.97 (s, 2H), 7.88 – 7.81 (m, 1H), 7.61 (d, J = 2.4 Hz, 1H), 7.56 (dd, J = 8.4, 2.4 Hz, 1H), 7.01 (d, J = 8.4 Hz, 1H), 3.91 (s, 3H), 2.18 (s, 6H), 2.11 (s, 3H), 1.81 (s, 6H). **^{13}C NMR** (100 MHz, $CDCl_3$) δ

191.9(t, J = 27 Hz), 159.1, 142.3, 139.1, 136.9, 134.3, 133.7(t, J = 3.1 Hz), 132.3, 131.4, 131.3, 129.9, 129.8, 129.2, 126.9, 126.0, 125.8, 125.0, 123.2, 112.2, 55.2, 40.6, 37.2, 37.1, 29.1.
HRMS (ESI) calcd for $C_{28}H_{28}DO_2$ $[M + H]^+$ 398.2225, found 398.2224.

4-((triisopropylsilyl)oxy)benzaldehyde-*formyl-d₁* (10tt).

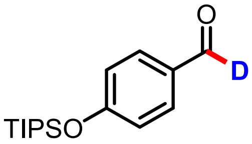

According to the *general procedure*. The spectral Data is consistent with the literature data.[5]
Yellow oil (77.0 mg, 92%).
R_f 0.40 (Petroleum ether/EtOAc, 40/1).
D incorporation by 1H NMR: 92%.
^{1}H NMR (400 MHz, $CDCl_3$) δ 9.88 (s, 0.08H), 7.91 – 7.70 (m, 2H), 7.10 – 6.92 (m, 2H), 1.29 (dd, J = 13.6, 6.8 Hz, 3H), 1.12 (t, J = 7.2 Hz, 18H). **^{13}C NMR** (100 MHz, $CDCl_3$) δ 190.6 (t, J = 27.5 Hz), 162.0, 132.0, 130.2 (t, J = 3.5 Hz), 120.4, 17.9, 12.8.
HRMS (ESI) calcd for $C_{16}H_{26}DO_2Si$ $[M + H]^+$ 280.1838, found 280.1838.

2,2,6,6-tetramethylpiperidin-1-yl 2-naphthoate (16).

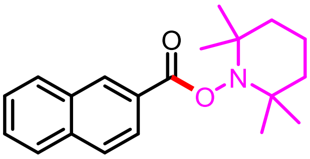

According to the *general procedure*.
White solid (11.2 mg, 12%). Mp: 100 − 101 °C.
R_f 0.36 (Petroleum ether/EtOAc, 20/1).
^{1}H NMR (400 MHz, $CDCl_3$) δ 8.64 (s, 1H), 8.10 (dd, J = 8.4, 1.6 Hz, 1H), 7.98 (d, J = 8.0 Hz, 1H), 7.90 (dd, J = 8.4, 5.2 Hz, 2H), 7.68 – 7.51 (m, 2H), 1.81 (dd, J = 23.2, 9.6 Hz, 2H), 1.73 (dd, J = 14.4, 5.2 Hz, 1H), 1.66 – 1.57 (m, 2H), 1.53 – 1.44 (m, 1H), 1.33 (s, 6H), 1.16 (s, 6H). **^{13}C NMR** (100 MHz, $CDCl_3$) δ 166.7, 135.6, 132.6, 131.0, 129.4, 128.3, 127.9, 127.0, 126.8, 125.4, 60.6, 39.2, 32.1, 21.0, 17.1.
HRMS (ESI) calcd for $C_{20}H_{26}NO_2$ $[M + H]^+$ 312.1958, found 312.1956.

benzyl 4-(naphthalen-2-yl)-4-oxobutanoate-2-d (18).

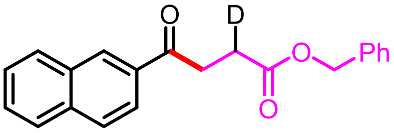

According to the *general procedure*.
White solid (15.3 mg, 16%). Mp: 81 − 82 °C.
R_f 0.36 (Petroleum ether/EtOAc, 40/1).
^{1}H NMR (400 MHz, $CDCl_3$) δ 8.51 (s, 1H), 8.04 (dd, J = 8.4, 1.6 Hz, 1H), 7.96 (d, J = 8.0 Hz, 1H), 7.93 – 7.84 (m, 2H), 7.66 – 7.52 (m, 2H), 7.39 – 7.31 (m, 5H), 5.17 (s, 2H), 3.47 (d, J = 6.8 Hz, 2H), 2.87 (t, J = 6.8 Hz, 1H). **^{13}C NMR** (100 MHz, $CDCl_3$) δ 198.1, 173.0, 136.0, 135.8, 134.0,

132.6, 129.9, 129.7, 128.7, 128.6, 128.5, 128.4, 128.3, 127.9, 126.9, 123.9, 66.7, 33.5, 28.3 (t, *J* = 20 Hz).

HRMS (ESI) calcd for $C_{21}H_{18}DO_3$ $[M + H]^+$ 320.1391, found 320.1387.

***S*-(2,4,6-triisopropylphenyl) pyridine-4-carbothioate (19).**

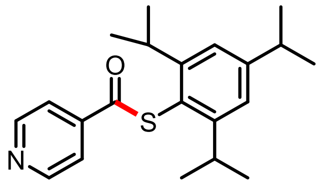

According to the *general procedure*.

White solid (85.9 mg, 84%). Mp: 104 − 105 °C.

R_f 0.40 (Petroleum ether/EtOAc, 20/1).

^{1}H NMR (400 MHz, $CDCl_3$) δ 8.83 (d, *J* = 5.6 Hz, 2H), 7.88 (d, *J* = 5.6 Hz, 2H), 7.14 (s, 2H), 3.39 (dt, *J* = 13.6, 6.8 Hz, 2H), 2.94 (dt, *J* = 13.6, 6.8 Hz, 1H), 1.29 (d, *J* = 6.8 Hz, 6H), 1.20 (d, *J* = 6.8 Hz, 12H). **^{13}C NMR** (100 MHz, $CDCl_3$) δ 190.1, 152.9, 151.8, 151.0, 143.2, 122.48, 120.8, 120.1, 34.5, 32.3, 24.6, 24.0.

HRMS (ESI) calcd for $C_{21}H_{28}NOS$ $[M + H]^+$ 342.1886, found 342.1884.

8. Procedures for organic transformations

Synthesis of (4-bromophenyl)methan-*d*$_2$-ol (11).

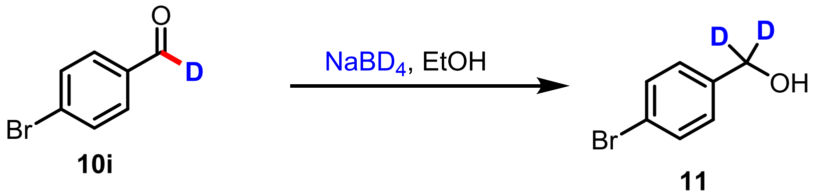

10i 11

A flame-dried flask was cooled under a stream of nitrogen and then charged with a solution of selected labeled aldehyde **10i** (0.2 mmol, 36.8 mg) and ethanol (1 ml). The solution was then cooled to 0 °C, and $NaBD_4$ (0.22 mmol, 9.2 mg, 1.1 equiv) was added slowly. The resulting solution was allowed to warm to rt and stirred for 6 h. The reaction mixture was then diluted with water and extracted twice with DCM. The combined organic layers were washed with brine, dried over anhydrous sodium sulfate, filtered, and concentrated in vacuo. The remaining residue purified directly via silica column chromatography, eluting with petroleum ether: ethyl acetate (1:1) to afford the corresponding product **11**.

(4-bromophenyl)methan-*d*$_2$-ol (11).

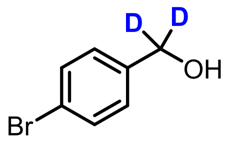

White solid (32.3 mg, 86%). Mp: 71 − 72 °C.

R_f 0.40 (Petroleum ether/EtOAc, 40/1).

^{1}H NMR (400 MHz, $CDCl_3$) δ 7.47 (d, *J* = 8.4 Hz, 2H), 7.21 (d, *J* = 8.4 Hz, 2H). **^{13}C NMR** (100 MHz, $CDCl_3$) δ 139.74, 131.72, 128.74, 121.57, 64.09 (dt, *J* = 33.8, 15.5 Hz).

HRMS (ESI) calcd for $C_7H_6D_2BrO$ $[M + H]^+$ 188.9879, found 188.9880.

Synthesis of *N*-((4-bromophenyl)methyl-d_2)-4-methylaniline (12).

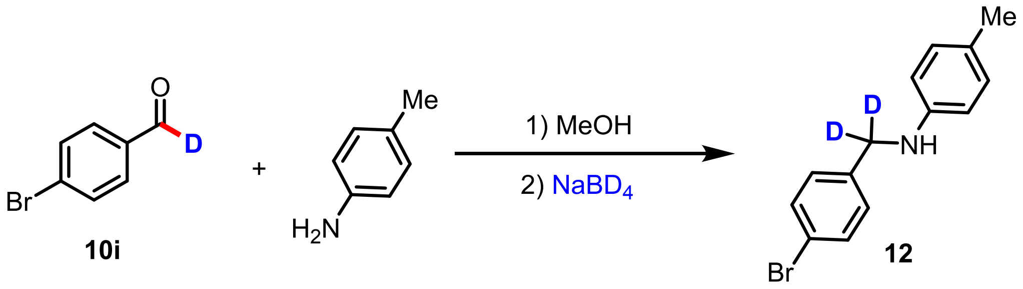

Following the modified procedures,[6] a flame-dried flask was cooled under a stream of nitrogen and then charged with a solution of aniline (0.2 mmol, 21.4 mg, 1.0 equiv), the selected labeled aldehyde **10i** (0.2 mmol, 36.8 mg), triethylamine (0.28 mmol, 28.3 mg, 1.4 equiv) and methanol (2 ml). This mixture was allowed to stir at rt for 4 h. The solution was then cooled to 0 °C, and $NaBD_4$ (0.22 mmol, 9.2 mg, 1.1 equiv) was added slowly. The resulting solution was allowed to warm to rt and stirred for 8 h. The reaction mixture was then diluted with water and extracted twice with hexanes. The combined organic layers were washed with brine, dried over anhydrous sodium sulfate, filtered, and concentrated in vacuo. The remaining residue purified directly via silica column chromatography, eluting with petroleum ether: ethyl acetate (20:1) to afford the corresponding product **12**.

***N*-((4-bromophenyl)methyl-d_2)-4-methylaniline (12).**

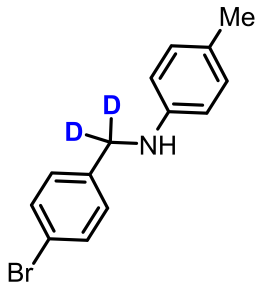

Yellow oil (48.8 mg, 88%).

R_f 0.40 (Petroleum ether/EtOAc, 40/1).

^{1}H NMR (400 MHz, $CDCl_3$) δ 7.41 – 7.31 (m, 2H), 7.20 – 7.11 (m, 2H), 6.90 (d, J = 8.0 Hz, 2H), 6.44 (d, J = 8.4 Hz, 2H), 3.83 (s, 1H), 2.15 (s, 3H). **^{13}C NMR** (100 MHz, $CDCl_3$) δ 145.7, 138.8, 131.8, 129.9, 129.2, 127.1, 121.0, 113.1, 20.5.

HRMS (ESI) calcd for $C_{14}H_{13}D_2BrN$ $[M + H]^+$ 278.0508, found 278.0507.

Synthesis of ethyl (*E*)-3-(4-bromophenyl)acrylate-3-*d* (13).

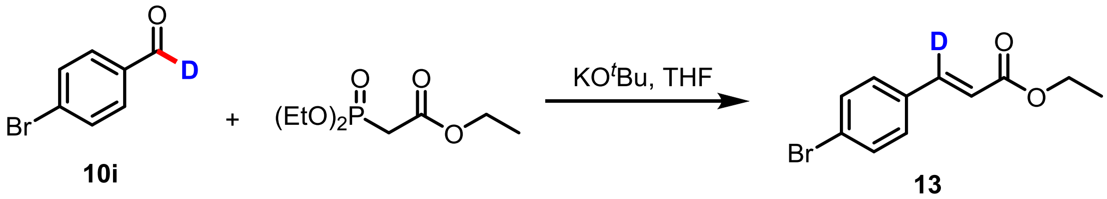

Following the modified procedures,[7] the selected labeled aldehyde **10i** (0.2 mmol, 36.8 mg) was dissolved in dry THF (2 mL) with stirring under an argon atmosphere. Sequentially, the HWE reagent, triethyl phosphonoacetate (0.22 mmol, 49.3 mg) and potassium tert-butoxide (0.22 mmol,

24.6 mg,) were then added to the flask, which was then left stirring at room temperature for 16 h. The THF solvent was removed in vacuo and the remaining residue purified directly via silica column chromatography, eluting with petroleum ether: ethyl acetate (20:1) to afford the corresponding product **13**.

ethyl (*E*)-3-(4-bromophenyl)acrylate-3-*d* (13).

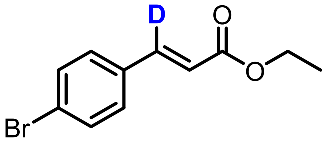

Yellow oil (62.0mg, 81%).

R_f 0.40 (Petroleum ether/EtOAc, 40/1).

^{1}H NMR (400 MHz, $CDCl_3$) δ 7.43 (d, J = 8.4 Hz, 2H), 7.30 (d, J = 8.4 Hz, 2H), 6.33 (s, 1H), 4.18 (q, J = 7.2 Hz, 2H), 1.26 (t, J = 7.2 Hz, 3H). **^{13}C NMR** (100 MHz, $CDCl_3$) δ 166.8, 143.0 (t, J = 24 Hz), 133.4, 132.2, 129.5, 124.6, 118.9, 60.7, 14.4.

HRMS (ESI) calcd for $C_{11}H_{11}DBrO_2$ $[M + H]^+$ 256.0078, found 256.0076.

Synthesis of [1,1':4',1''-terphenyl]-4-carbaldehyde-*formyl-d₁* (14).

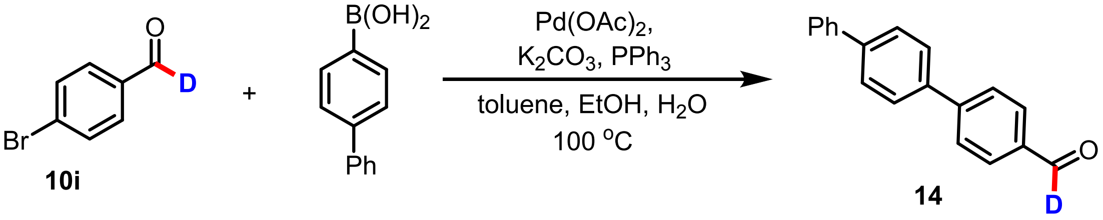

Figure S5

Synthesized pure compound **10i** (0.3 mmol, 1.0 equiv), boronic acid (0.33 mmol, 1.1 equiv), K_2CO_3 (0.6 mmol, 2.0 equiv), $Pd(OAc)_2$ (0.015 mmol, 5.0 mol %), PPh_3 (0.045 mmol, 0.15 equiv), toluene (3.3 mL, 0.113M), equal mixture of ethanol/water (0.34 mL, 0.565 M) were taken into a re-sealable pressure tube (13 x 100 mm) and was allowed it to stir at 100 °C for 24h. After finishing the reaction, the solvent mixture was evaporated and again diluted with dicholomethane (20 mL). This diluted mixture was then passed through a celite bed followed by the washing of this bed with additional amount of dicholomethane (20 mL). This combined organic layer was washed with water (1 x 20 mL) using a separating funnel. The collected organic layer was dried over $MgSO_4$ and solvent was evaporated under reduced pressure. This crude product was then subjected to purification using flash column chromatography petroleum ether: ethyl acetate (20:1) to get pure product **14**.

[1,1':4',1''-terphenyl]-4-carbaldehyde-*formyl-d₁* (14).

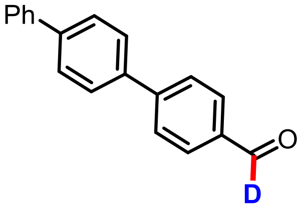

White solid (64.5 mg, 83%). Mp: 181 − 182 °C.

R_f 0.40 (Petroleum ether/EtOAc, 40/1).

D incorporation by 1H NMR: 92%.

^{1}H NMR (400 MHz, $CDCl_3$) δ 9.95 (s, 0.08H), 7.86 (d, J = 8.0 Hz, 2H), 7.69 (d, J = 8.0 Hz, 2H), 7.61 (s, 4H), 7.55 (d, J = 7.2 Hz, 2H), 7.37 (t, J = 7.2 Hz, 2H), 7.29 (t, J = 7.2 Hz, 1H). **^{13}C NMR** (100 MHz, $CDCl_3$) δ 191.71 (t, J = 26 Hz), 146.73, 141.44, 140.37, 138.57, 135.23 (t, J = 3.5 Hz), 130.43, 129.01, 127.84, 127.81, 127.78, 127.61, 127.17.

HRMS (ESI) calcd for $C_{19}H_{14}DO$ $[M + H]^+$ 260.1180, found 260.1180.

Synthesis of 4-(phenylethynyl)benzaldehyde-*formyl-d₁* (15).

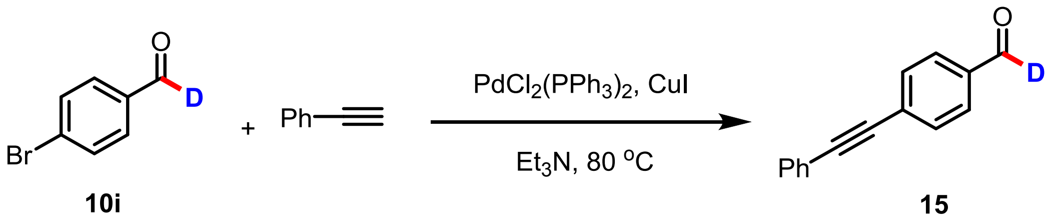

An oven dried Schlenk tube was charged with the selected labeled aldehyde **10i** (0.2 mmol, 36.8 mg), bis(triphenylphosphine)palladium(II) chloride (7.0 mg, 0.01 mmol), copper iodide (3.8 mg, 0.02 mmol) and Et_3N (1 mL). The tube was evacuated and backfilled with Ar (this process was repeated three times) at -40 °C and then phenylacetylene (33 µL, 0.3 mmol) was added by syringe. The reaction mixture was stirred at 80 °C for 12 h until the consumption of **10i**, indicated by TLC. The reaction mixture was filtered through celite pad and concentrated in vacuo to give the crude product, which was purified by common column chromatography petroleum ether: ethyl acetate (20:1) to give desired compound **15.**

4-(phenylethynyl)benzaldehyde-*formyl-d₁* (15).

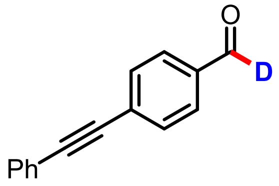

White solid (32.3 mg, 78%). Mp: 86 – 87 °C.

R_f 0.40 (Petroleum ether/EtOAc, 40/1).

D incorporation by 1H NMR: 95%.

^{1}H NMR (400 MHz, $CDCl_3$) δ 10.00 (s, 0.05H), 7.85 (d, J = 8.4 Hz, 2H), 7.66 (d, J = 8.4 Hz, 2H), 7.61 – 7.51 (m, 2H), 7.41 – 7.32 (m, 3H). **^{13}C NMR** (100 MHz, $CDCl_3$) δ 191.2 (t, J = 28.5 Hz), 135.4 (t, J = 3.0 Hz), 132.2, 131.9, 129.7, 129.1, 128.6, 122.6, 93.5, 88.6.

HRMS (ESI) calcd for $C_{15}H_{10}DO$ $[M + H]^+$ 208.0867, found 208.0867.

9. Gram-scale Reaction

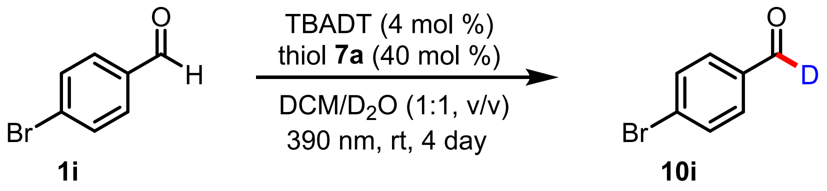

To an oven dried Schlenk tube was added was added TBADT (1.2 g, 0.36 mmol, 4 mol %), 4-bromobenzaldehyde **1i** (9 mmol, 1.0 equiv), thiol **7a** (0.84 g, 3.6 mmol, 40 mol %) and DCM/D_2O

(1:1, v/v; 90 mL). The tube was evacuated and backfilled with Ar (this process was repeated three times). The mixture was then stirred rapidly and irradiated with a 36 W 390 nm LED (approximately 2 cm away from the light source) at room temperature for 4 days. The reaction mixture was diluted with 100 mL of aqueous 1 M $NaHCO_3$ solution, and extracted with DCM (3 × 50 mL). The combined organic extracts were washed with brine (150 mL), dried over Na_2SO_4, and concentrated in vacuo. Purification of the crude product by flash chromatography on silica gel using the indicated solvent system afforded the desired product **10i** in 93% yield and 96% D incorporation.

References

(1) I. B. Perry, T. F. Brewer, P. J. Sarver, D. M. Schultz, D. A. DiRocco, D. W. C. MacMillan, *Nature* 2018, **560**, 70.

(2) M. Zhang, X.-A. Yuan, C. -J. Zhu and J. Xie, *Angew. Chem. Int. Ed.* 2019, **58**, 312.

(3) F. Li, Y. Zhou, H. Yang, D. Liu, B. Sun, F.-L. Zhang, *Org. Lett.* 2018, **20**, 146.

(4) a) J. D. Debad, J. C. Morris, P. Magnus, A. J. Bard, *J. Org. Chem.* 1997**, 62***,* 530; b) S. Rashidnadimi, T. H. Hung, K. T. Wong, A. J. Bard, *J. Am. Chem. Soc*. 2008, **130**, 634.

(5) W. J. Kerr, M. Reid, T. Tuttle, *Angew. Chem. Int. Ed.* 2017, **56**, 7808.

(6) R. K. Everett, J. P. Wolfe, *J. Org. Chem.* 2015, **80**, 9041.

(7) W. J. Kerr, M. Reid, T. Tuttle, *Angew. Chem. Int. Ed.* 2017, **56**, 7808.

NMR Spectra

^{1}H NMR spectrum of compound **10a**

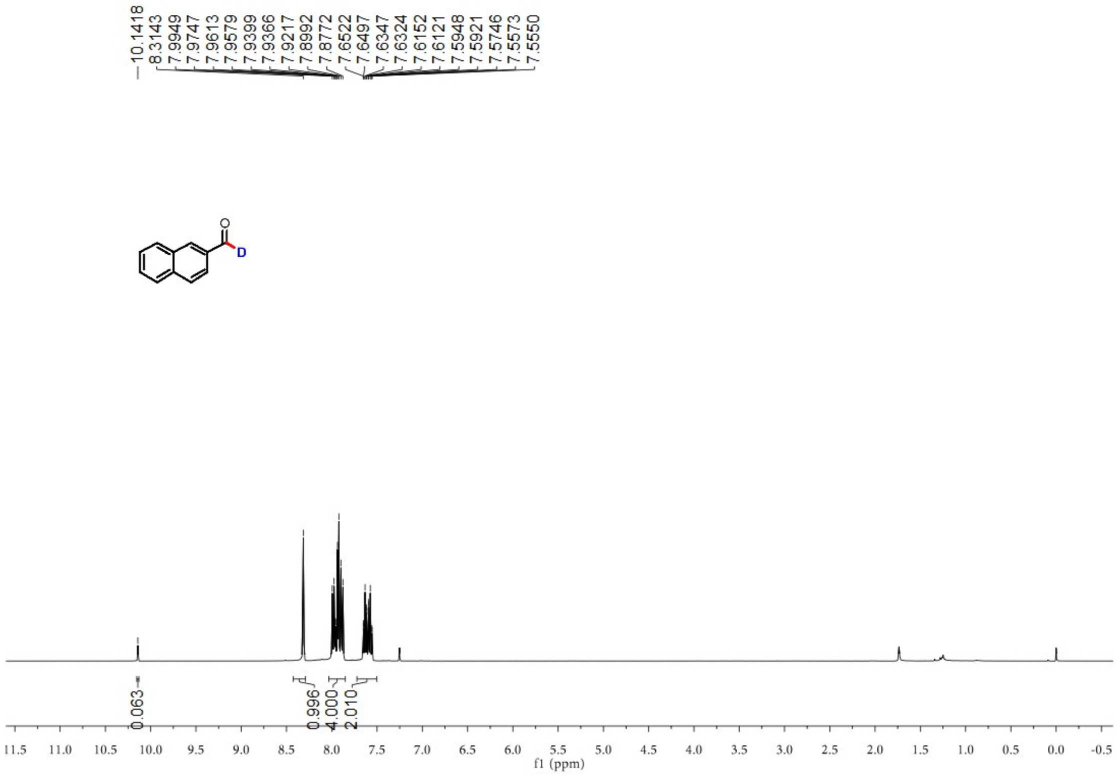

^{13}C NMR spectrum of compound **10a**

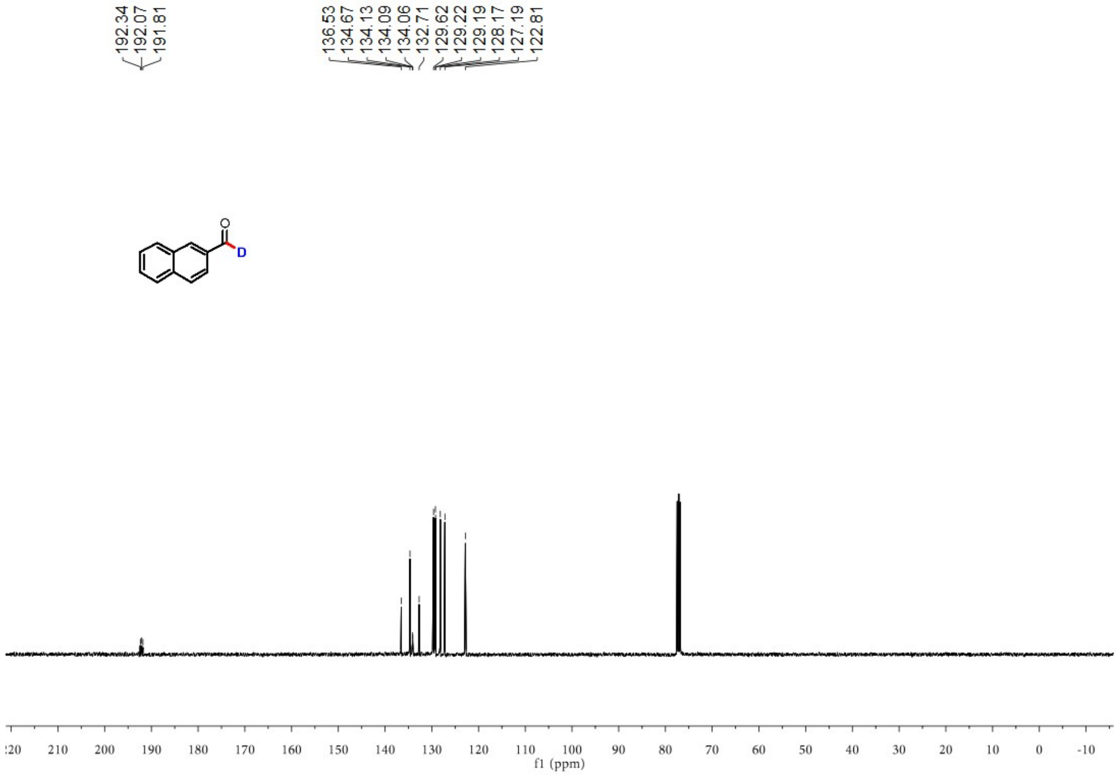

^{1}H NMR spectrum of compound **10b**

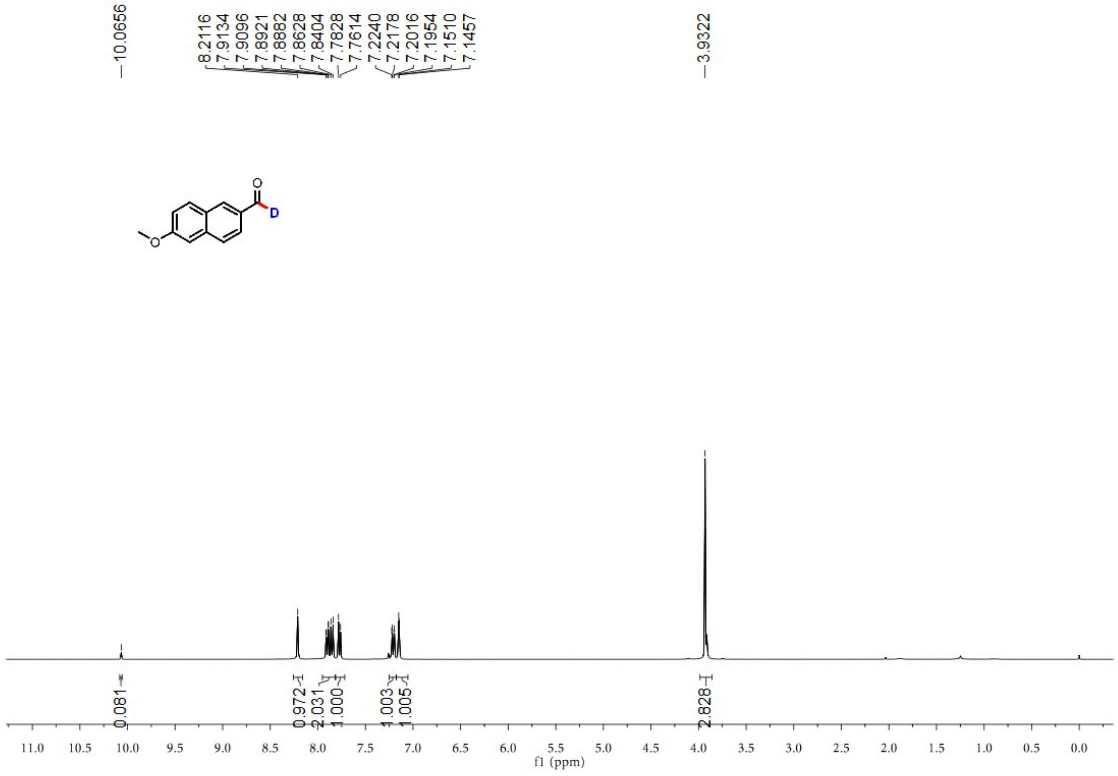

^{13}C NMR spectrum of compound **10b**

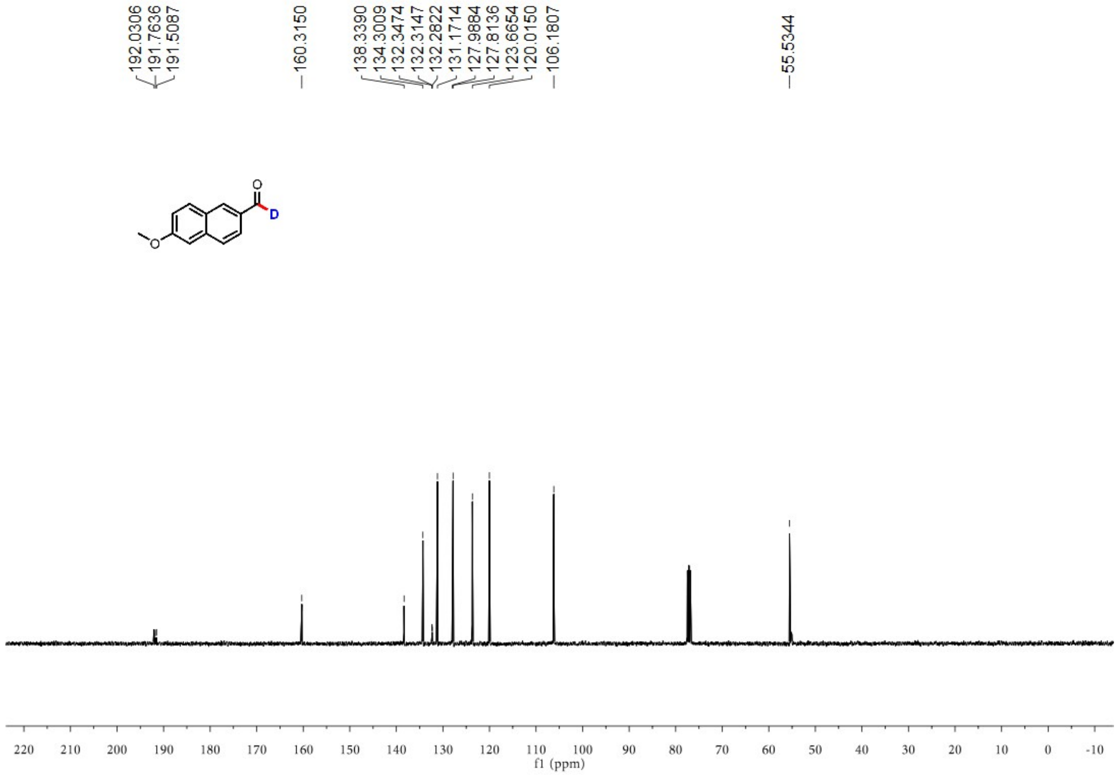

^{1}H NMR spectrum of compound **10c**

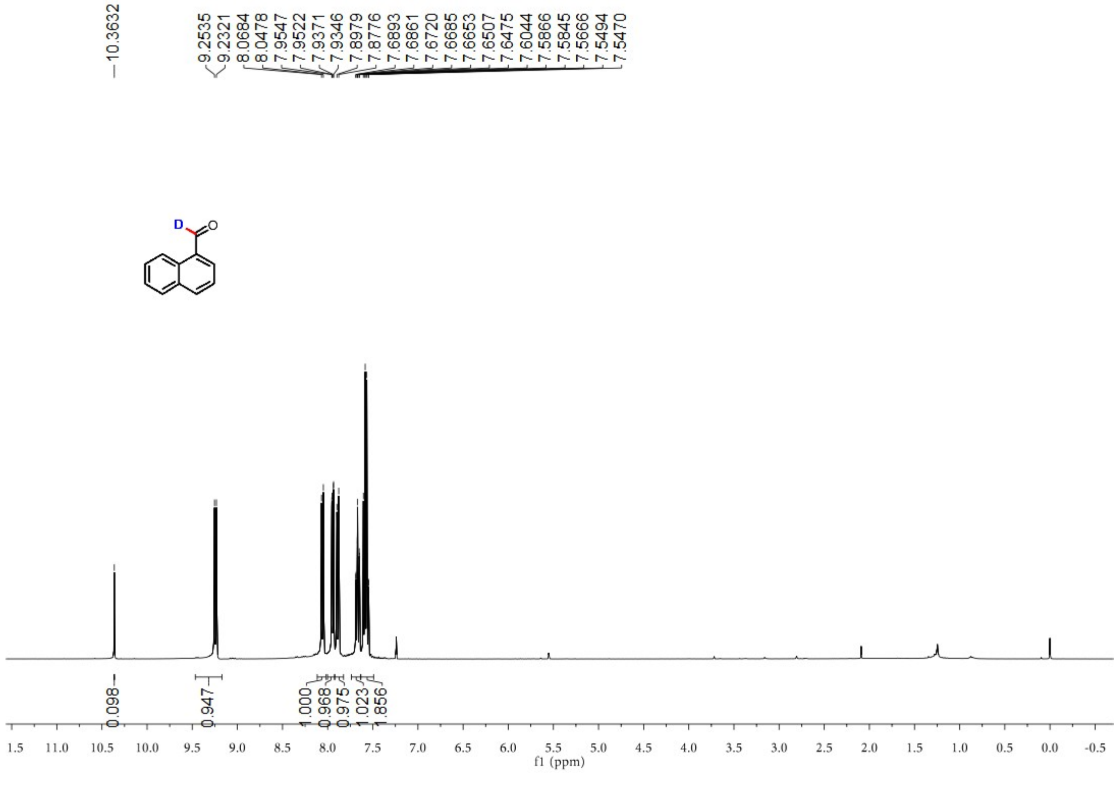

^{13}C NMR spectrum of compound **10c**

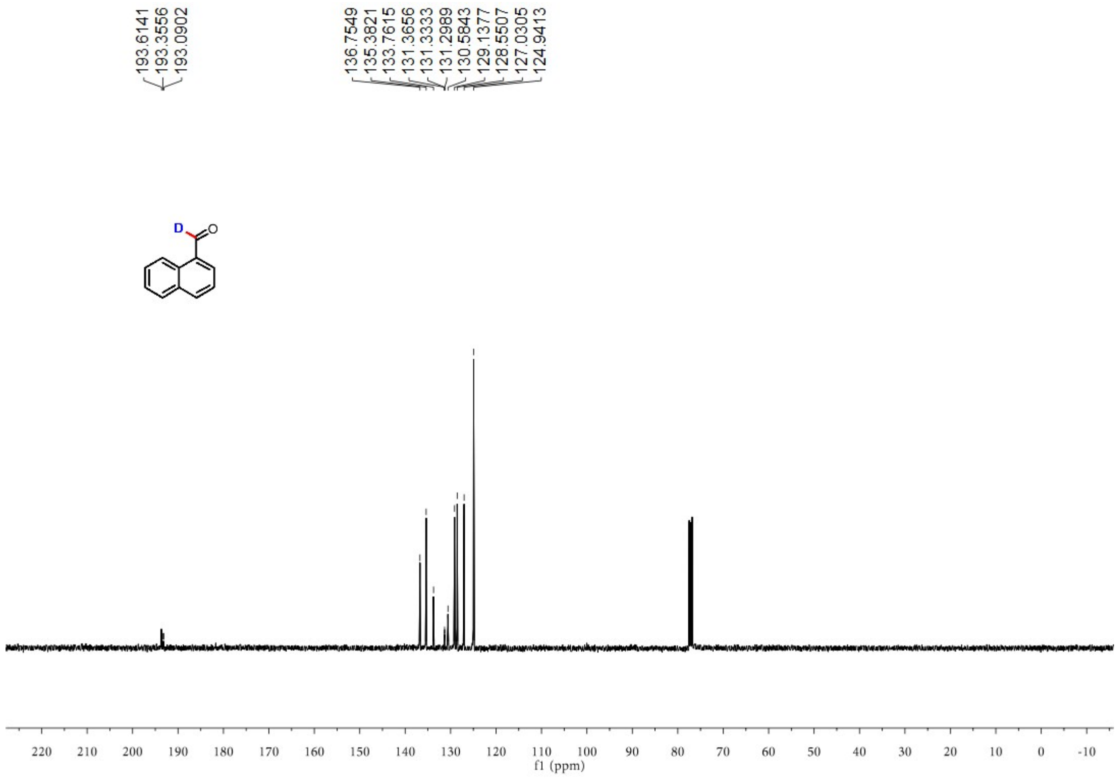

^{1}H NMR spectrum of compound **10d**

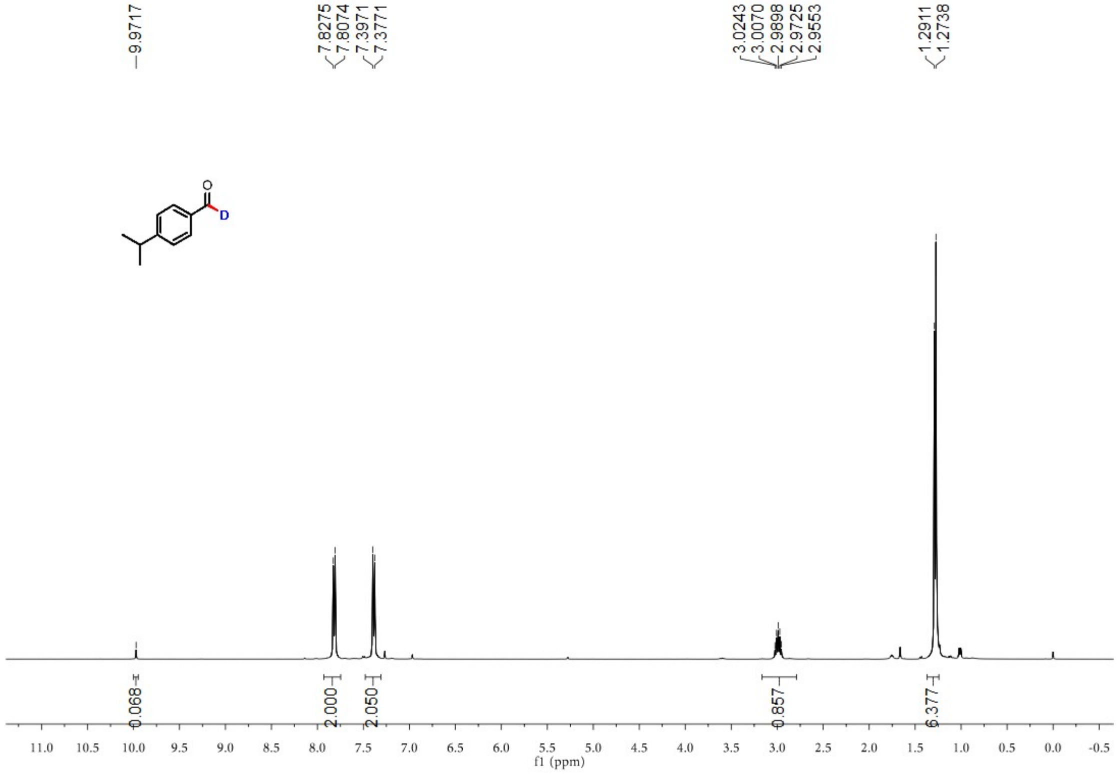

^{13}C NMR spectrum of compound **10d**

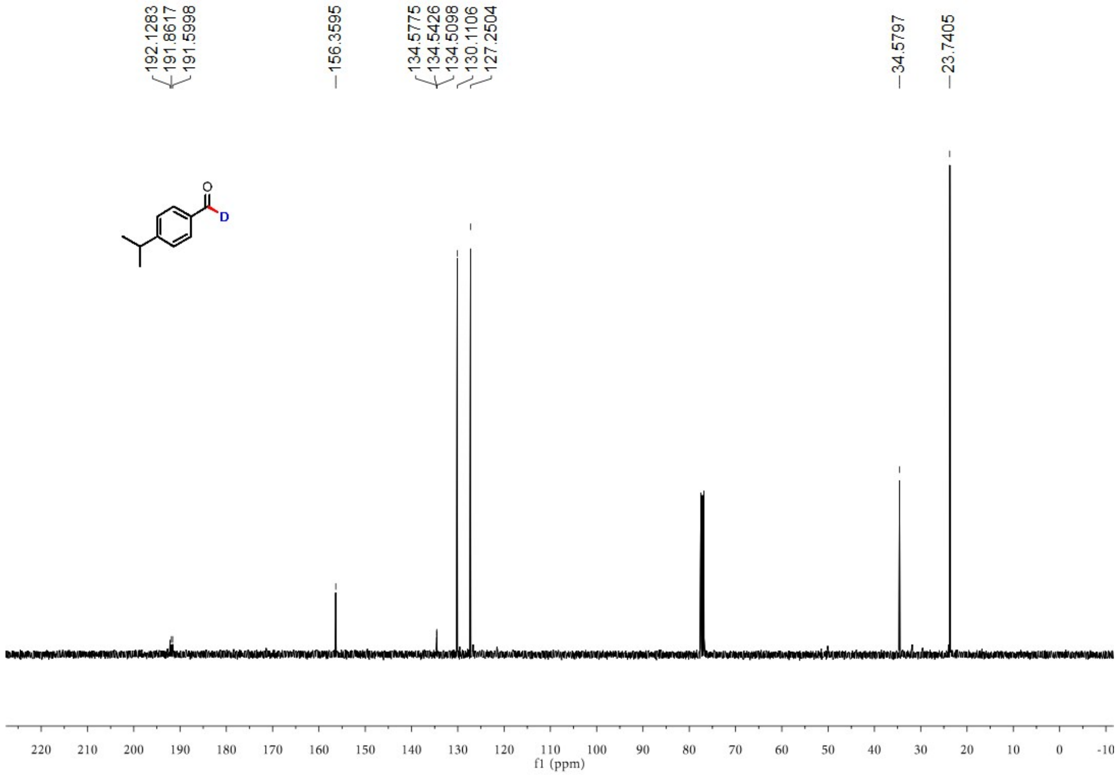

^{1}H NMR spectrum of compound **10e**

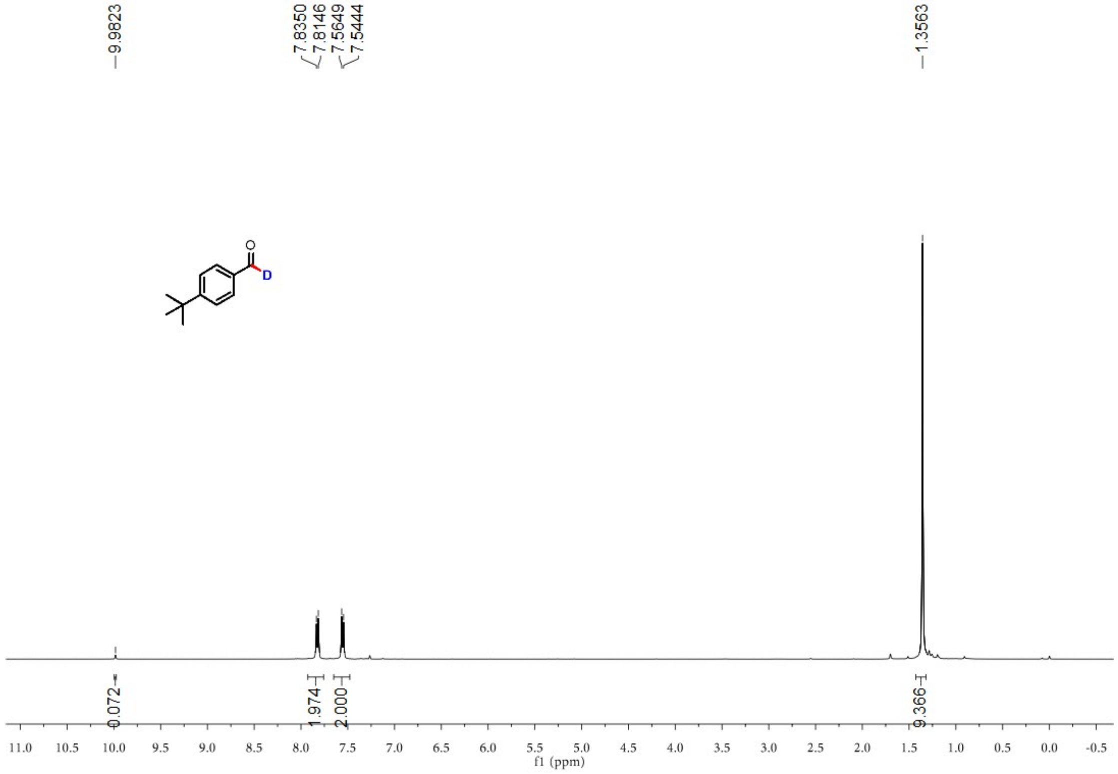

^{13}C NMR spectrum of compound **10e**

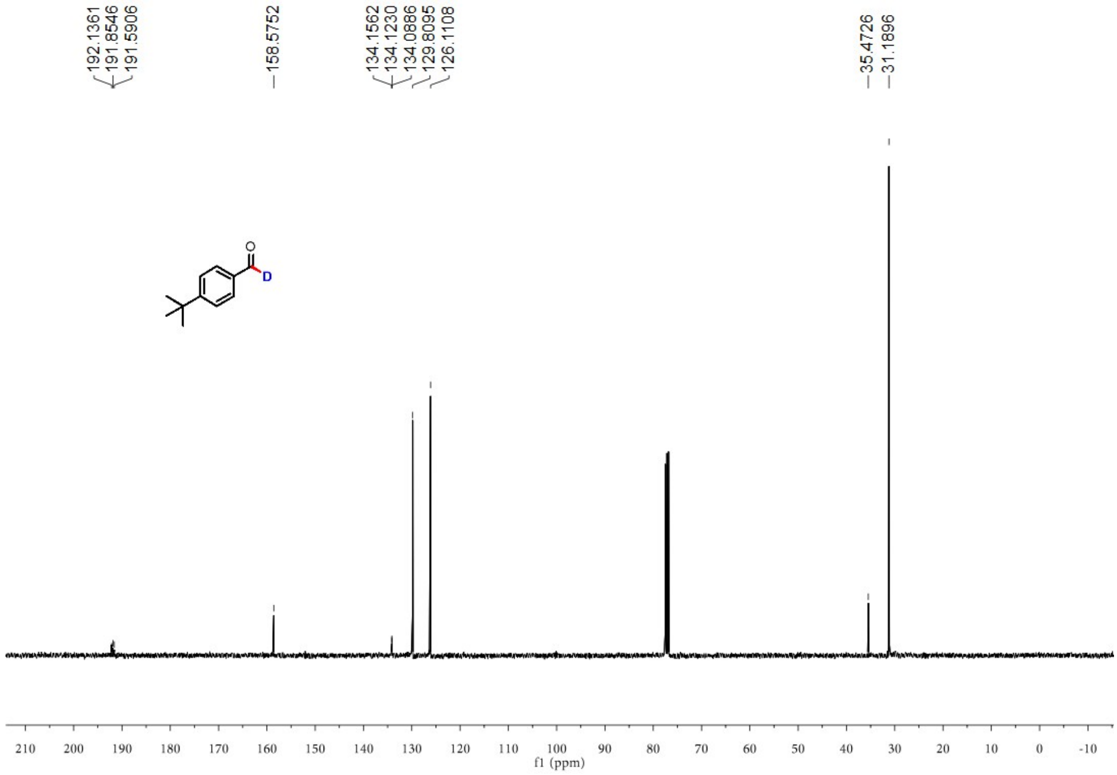

^{1}H NMR spectrum of compound **10f**

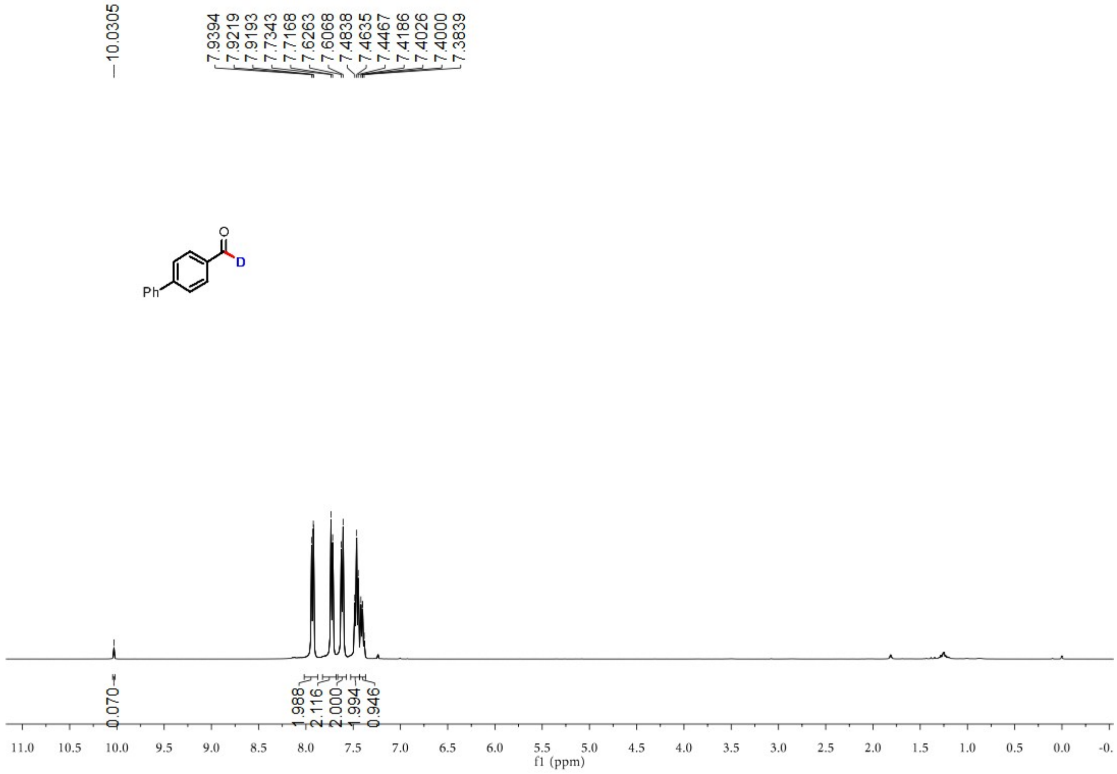

^{13}C NMR spectrum of compound **10f**

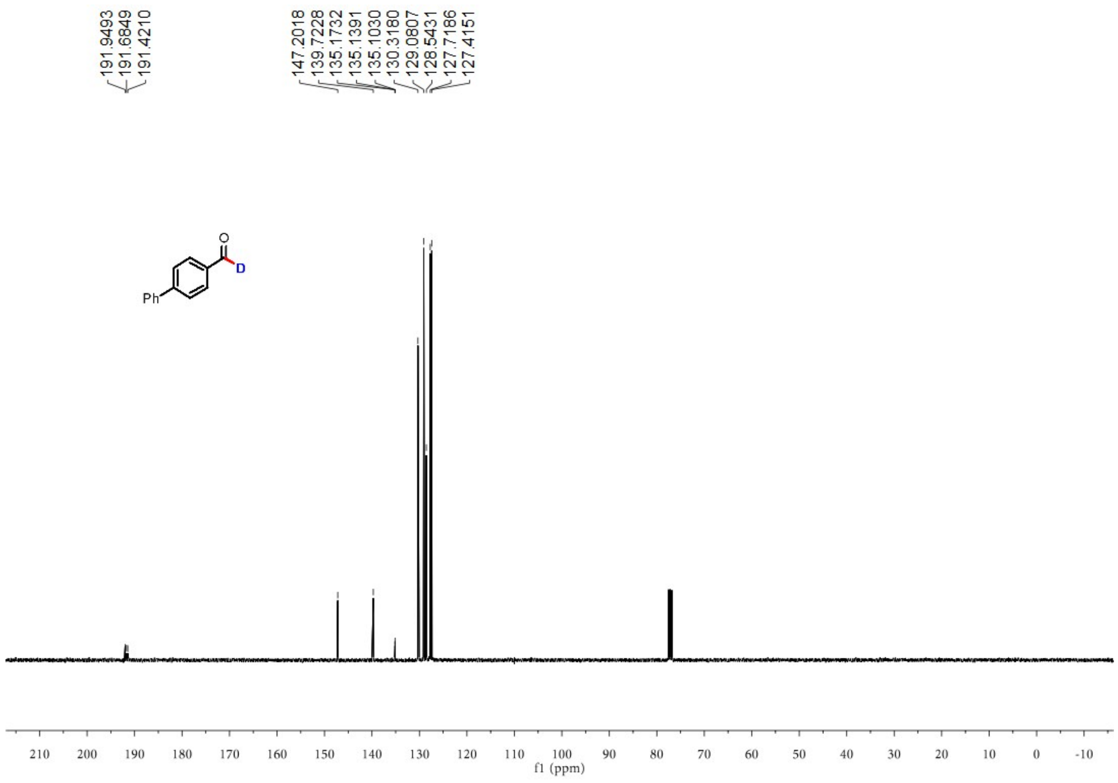

¹H NMR spectrum of compound **10g**

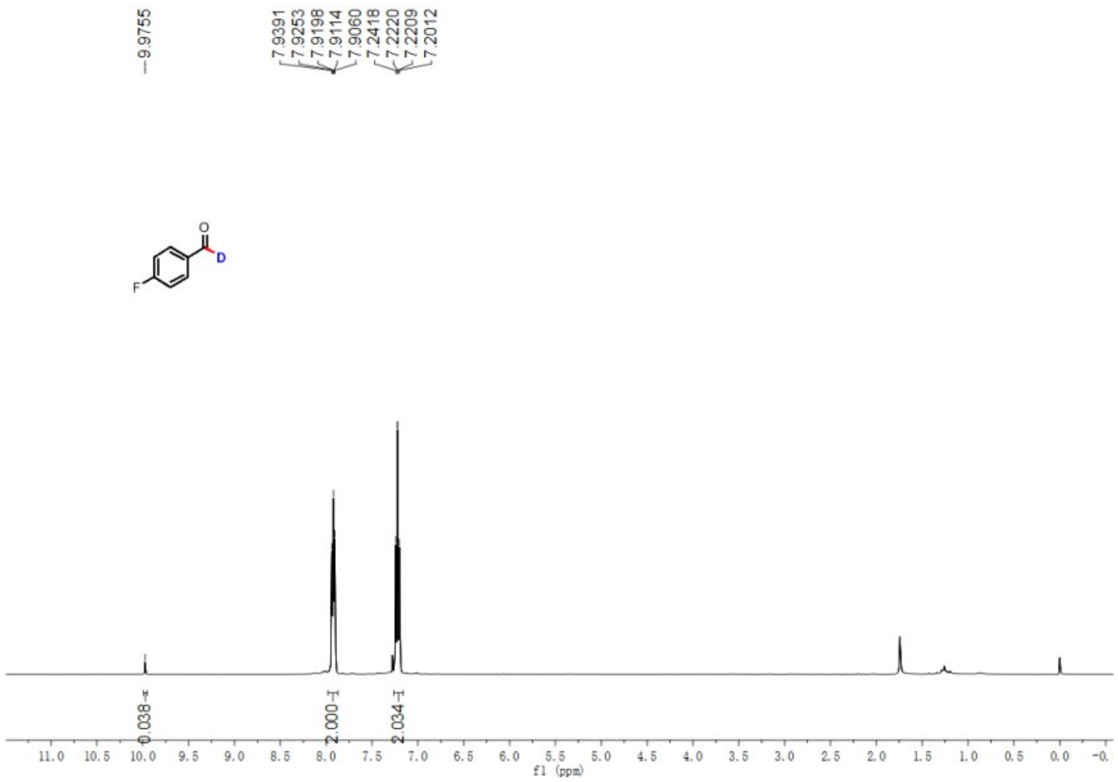

^{13}C NMR spectrum of compound **10g**

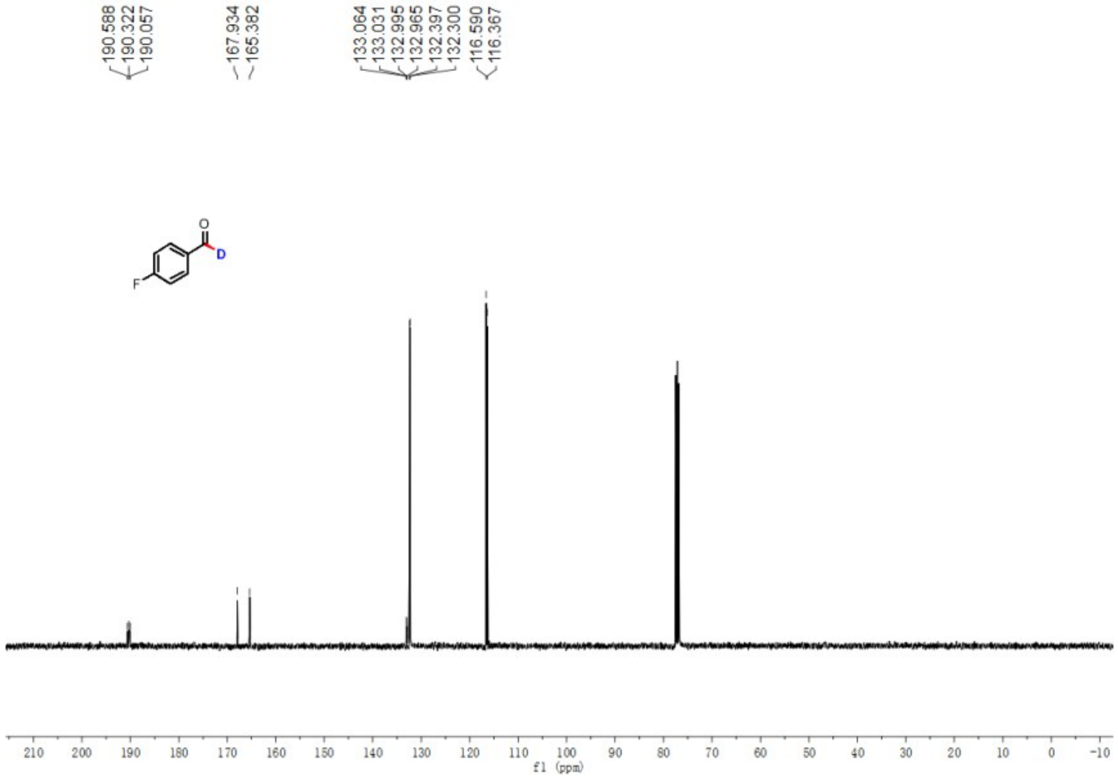

^{1}H NMR spectrum of compound **10h**

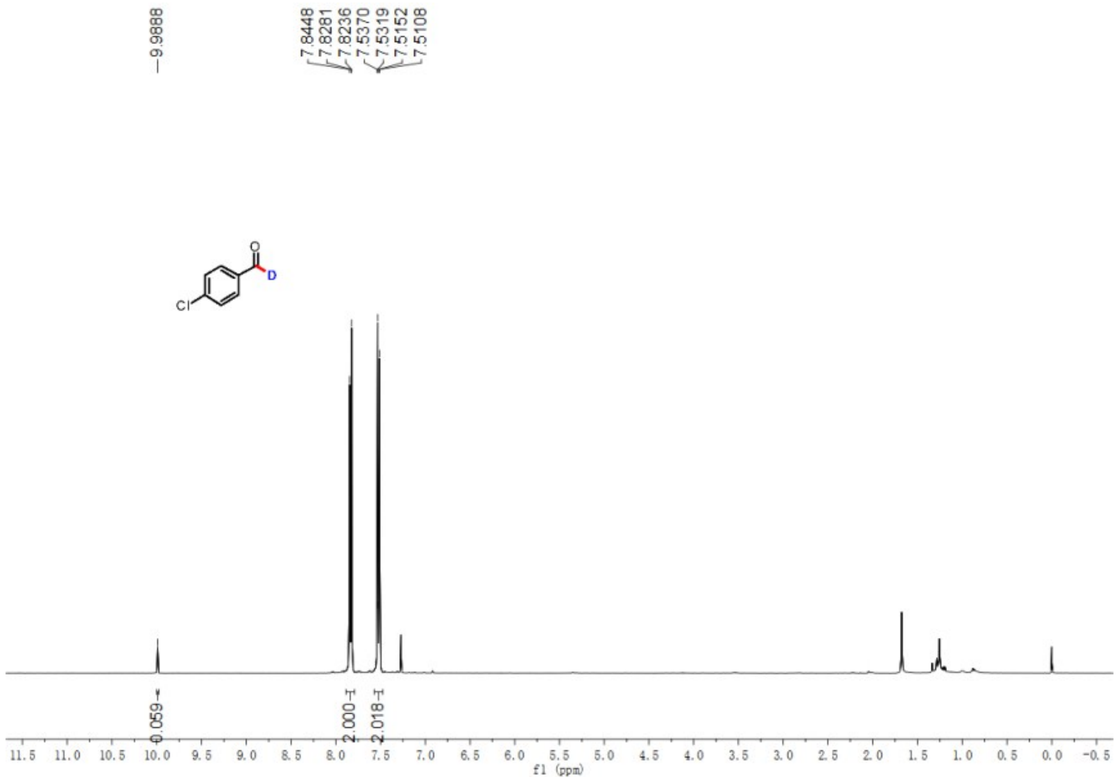

^{13}C NMR spectrum of compound **10h**

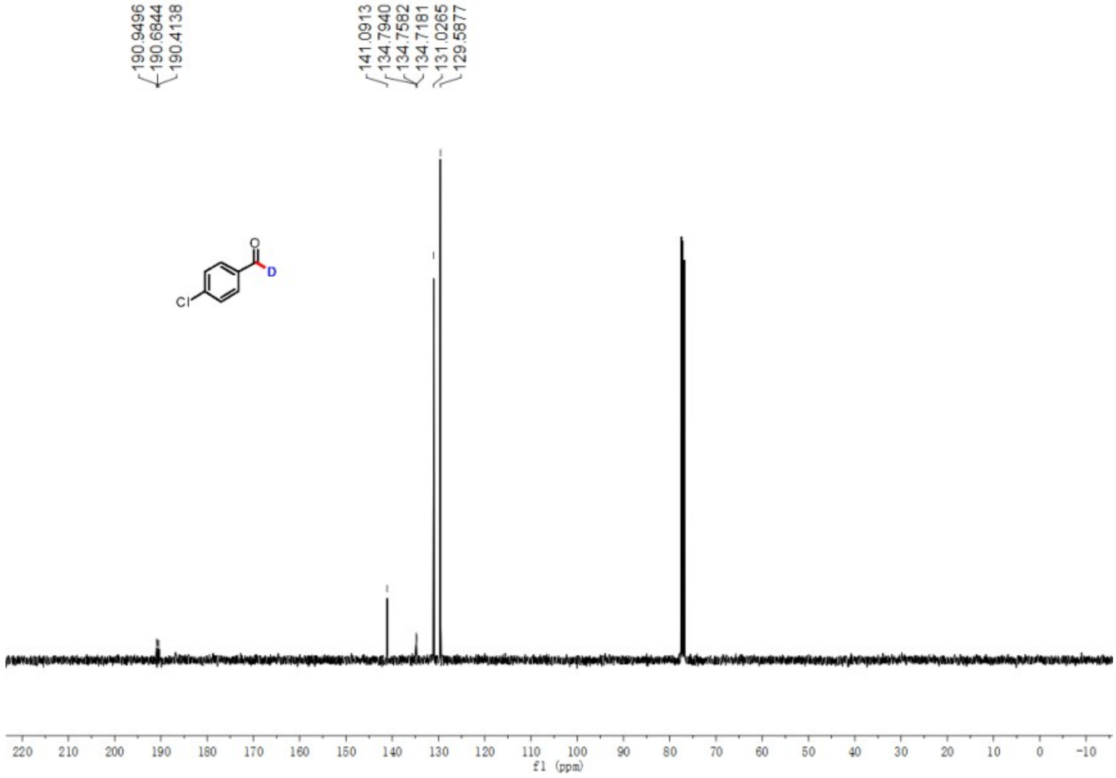

^{1}H NMR spectrum of compound **10i**

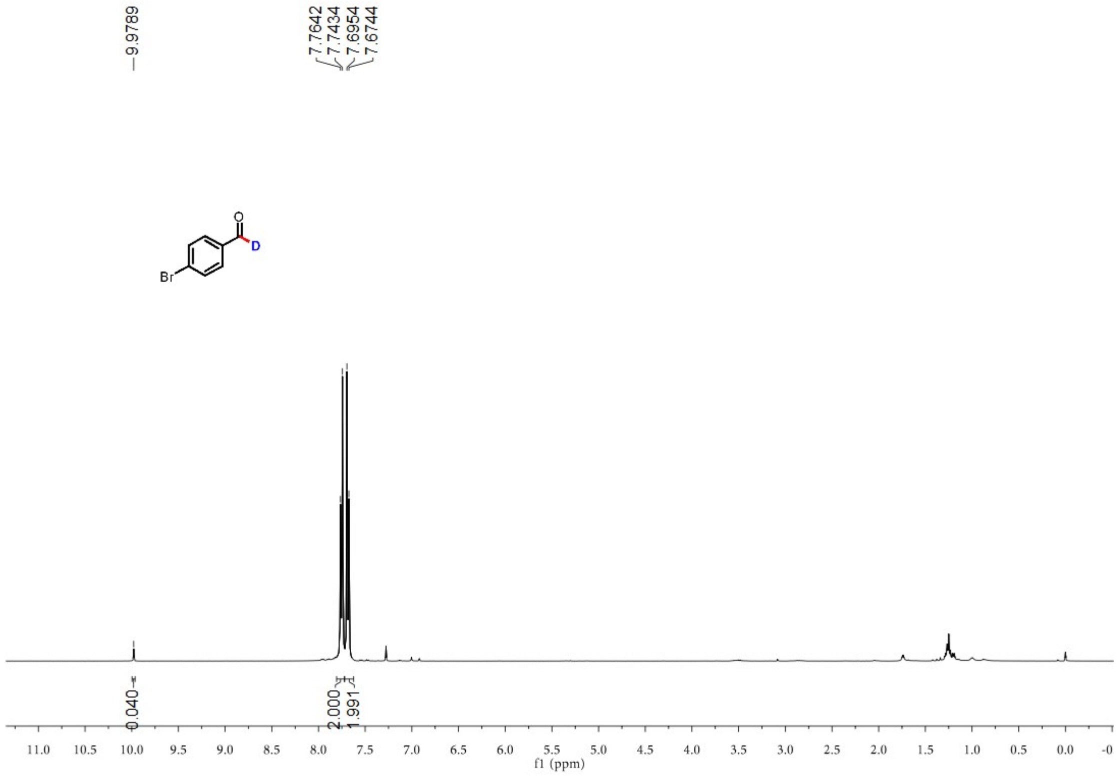

^{13}C NMR spectrum of compound **10i**

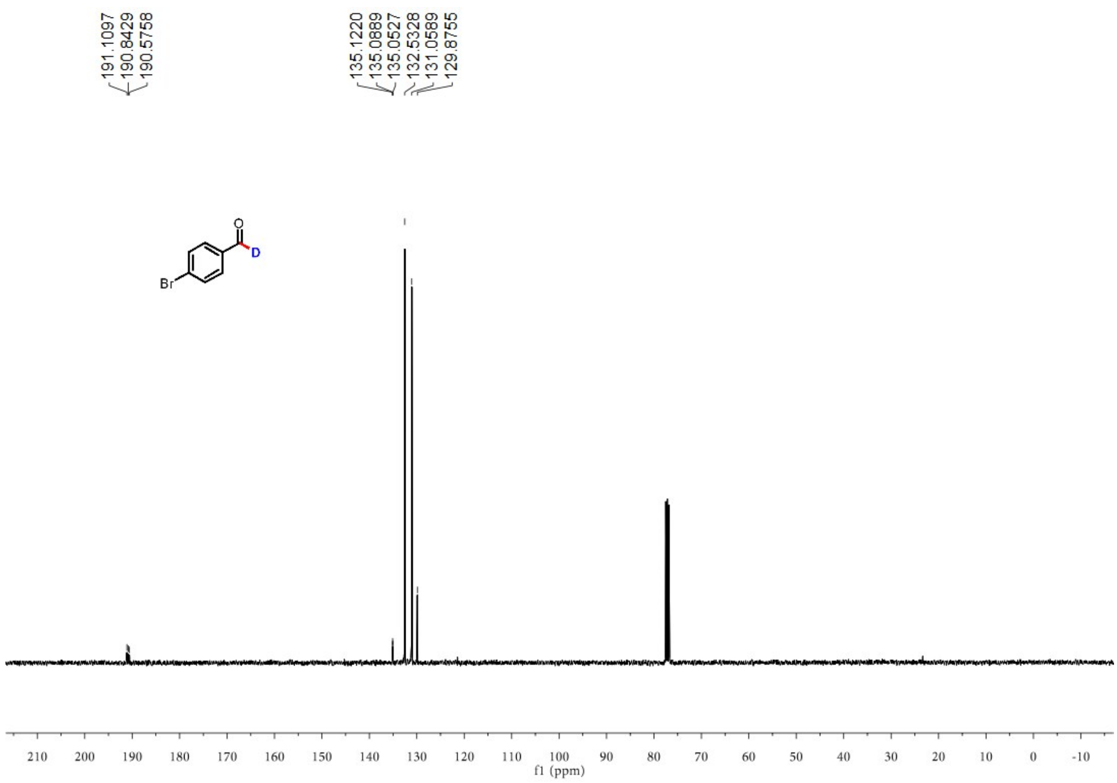

^{1}H NMR spectrum of compound **10j**

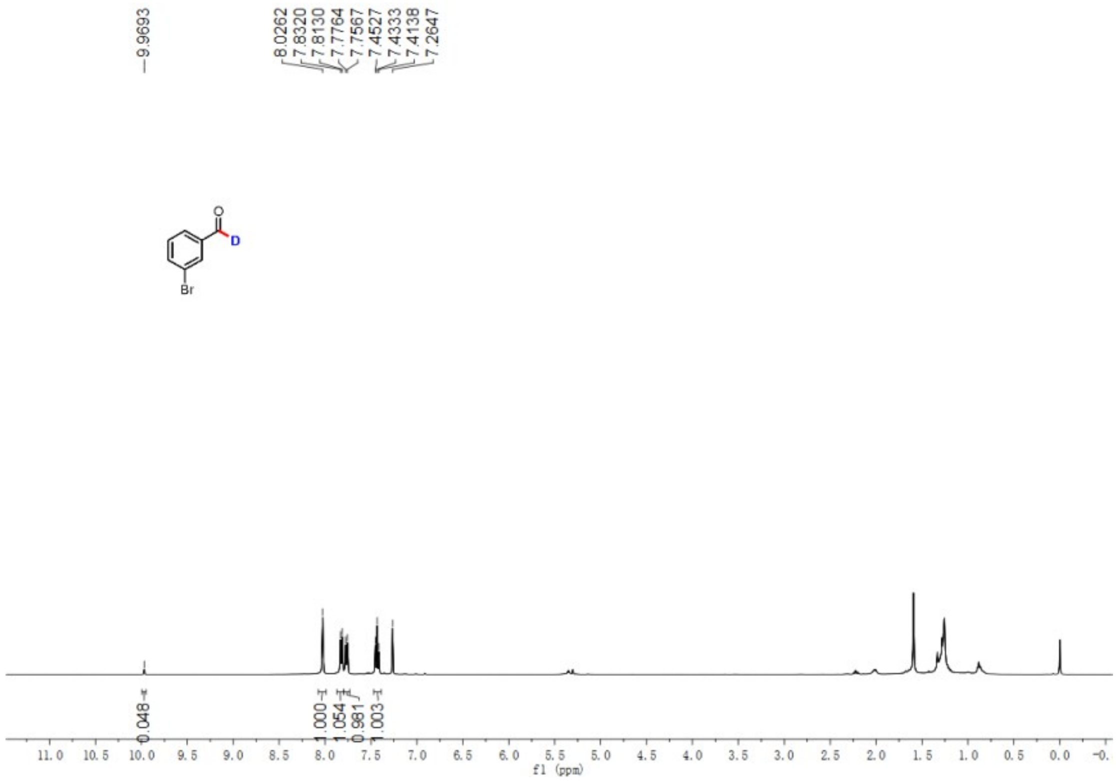

^{13}C NMR spectrum of compound **10j**

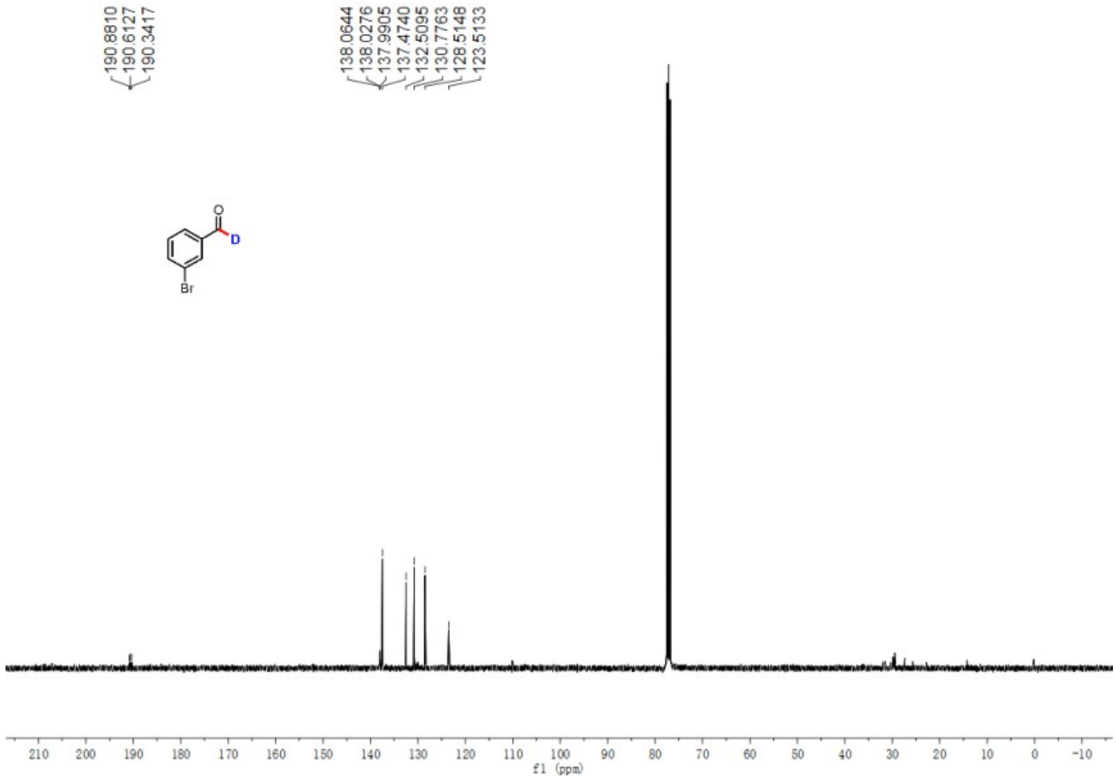

^{1}H NMR spectrum of compound **10k**

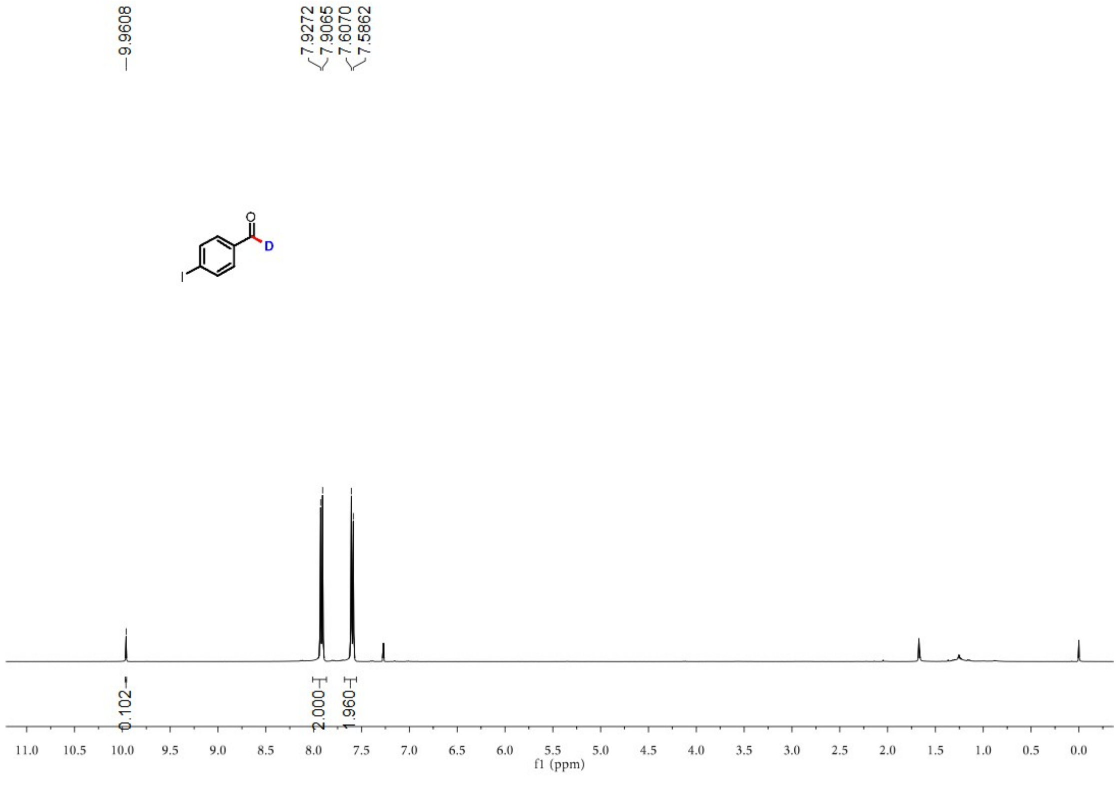

^{13}C NMR spectrum of compound **10k**

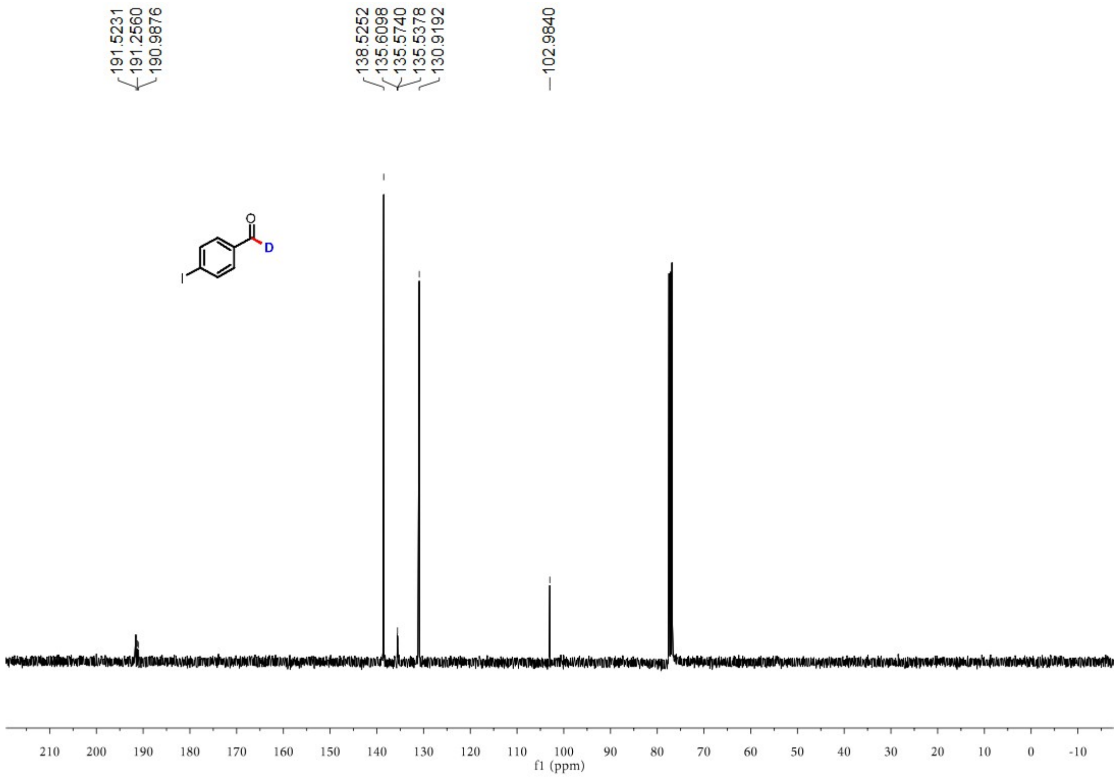

^1H NMR spectrum of compound **10l**

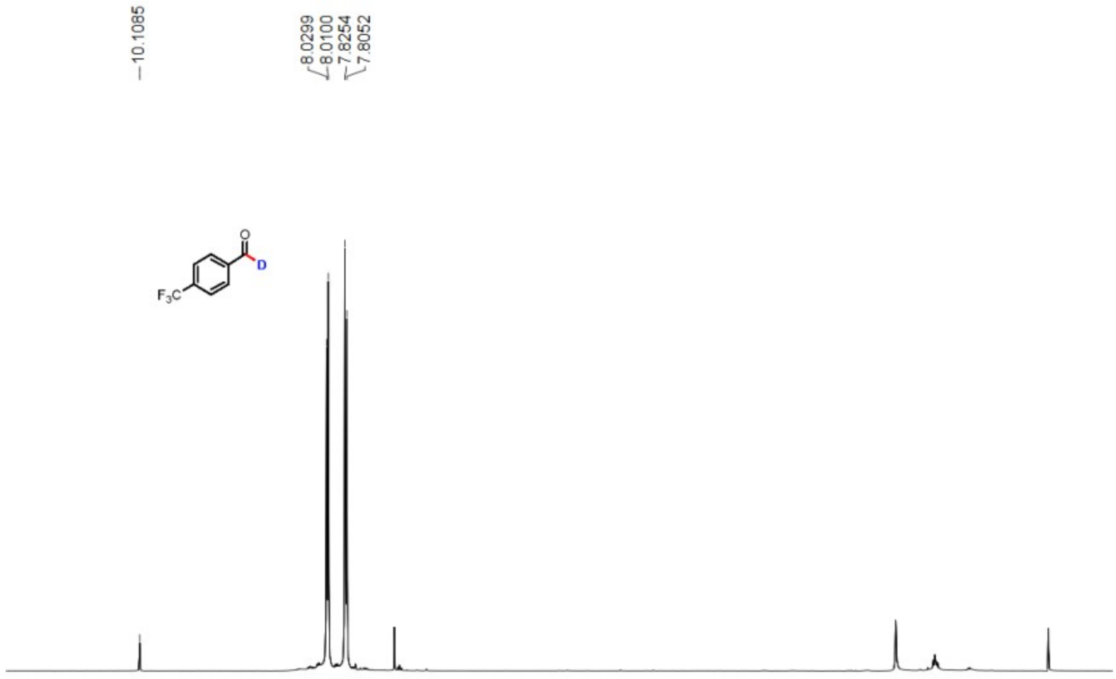

^{13}C NMR spectrum of compound **10l**

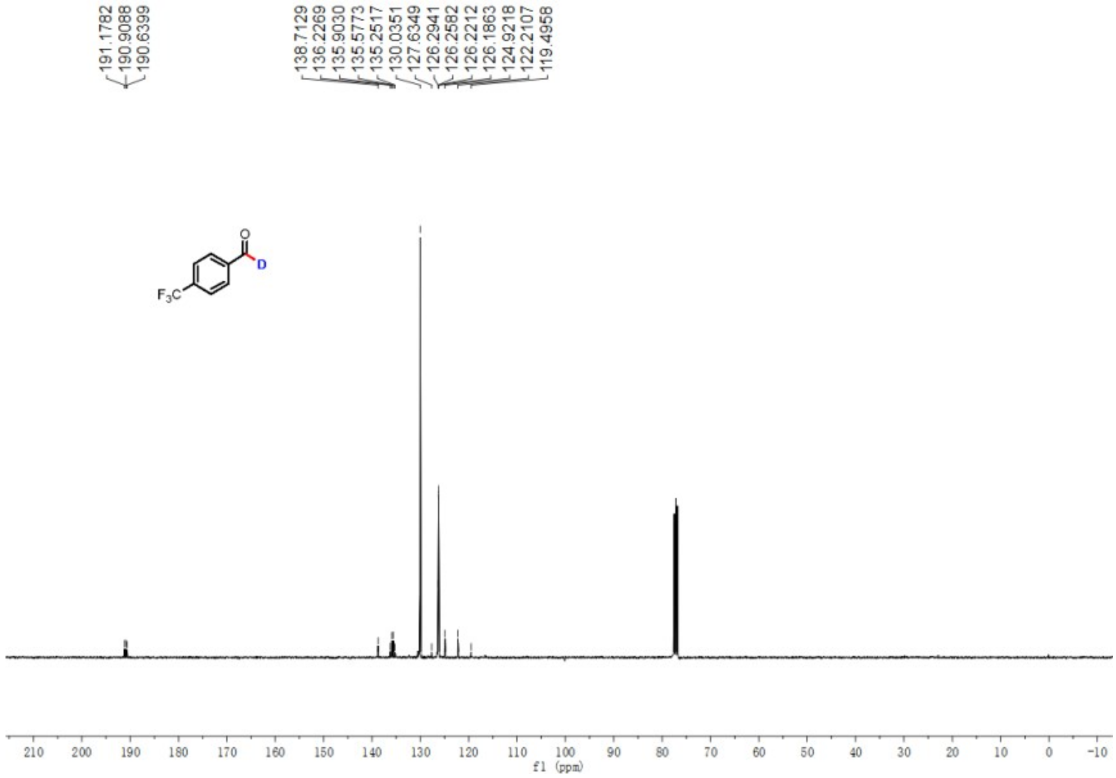

^{1}H NMR spectrum of compound **10m**

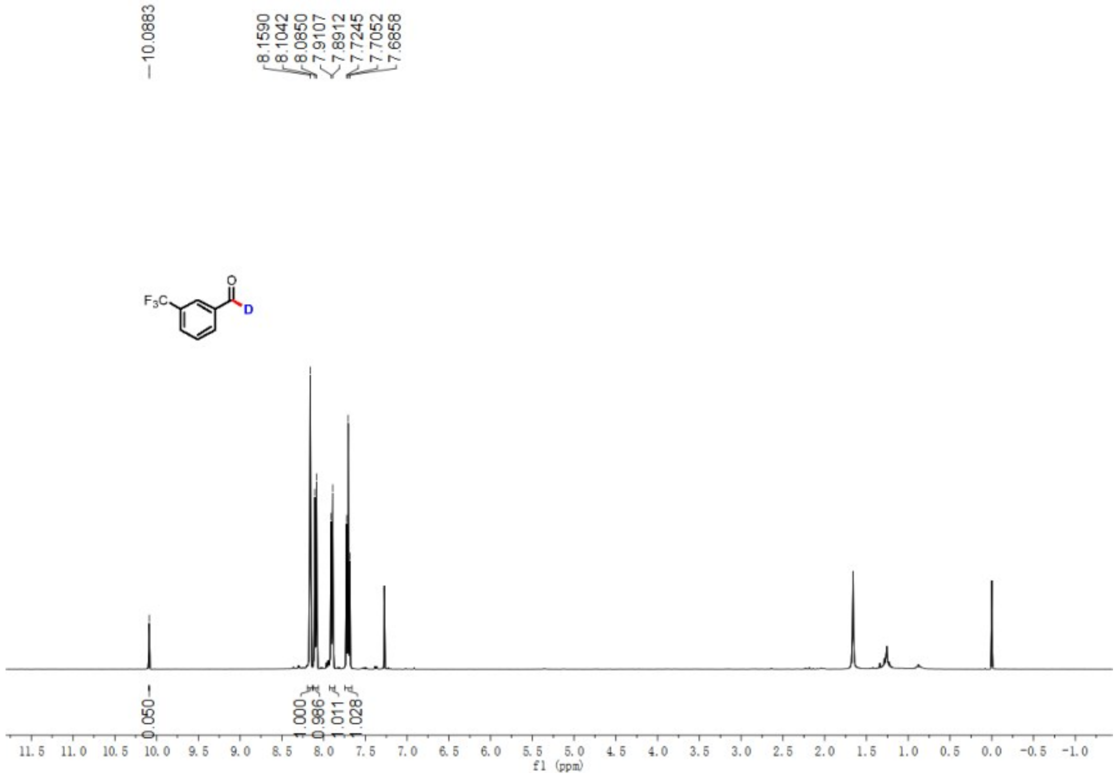

^{13}C NMR spectrum of compound **10m**

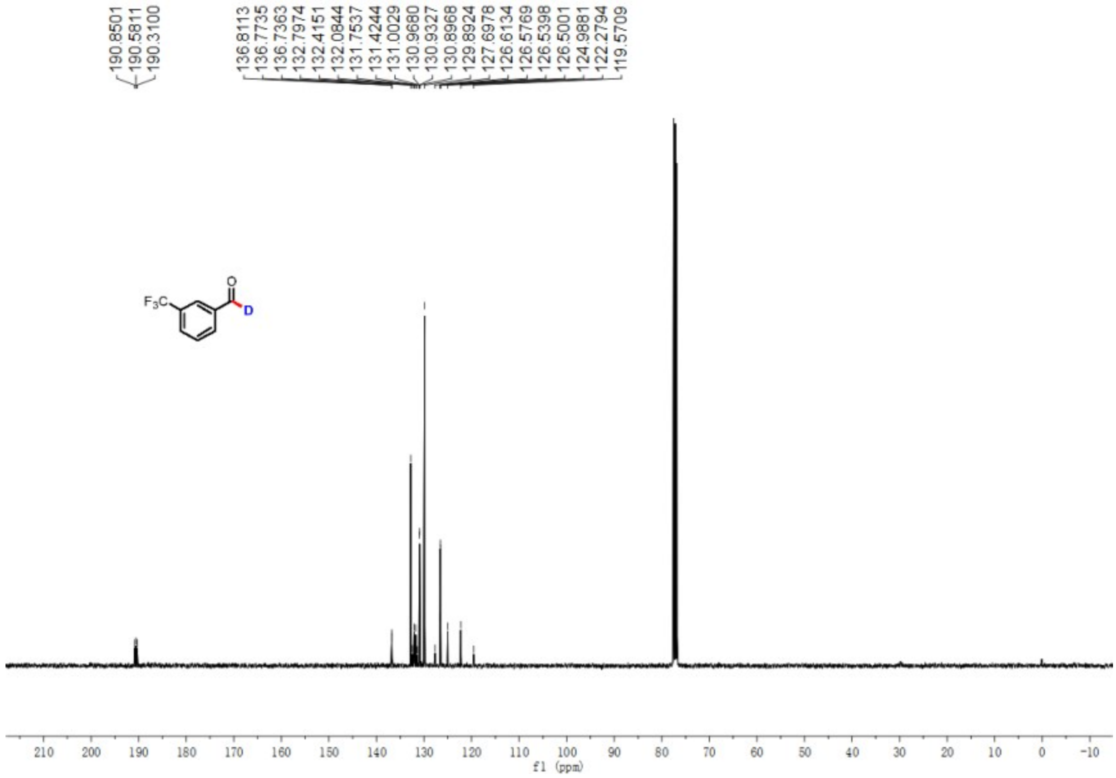

^{1}H NMR spectrum of compound **10n**

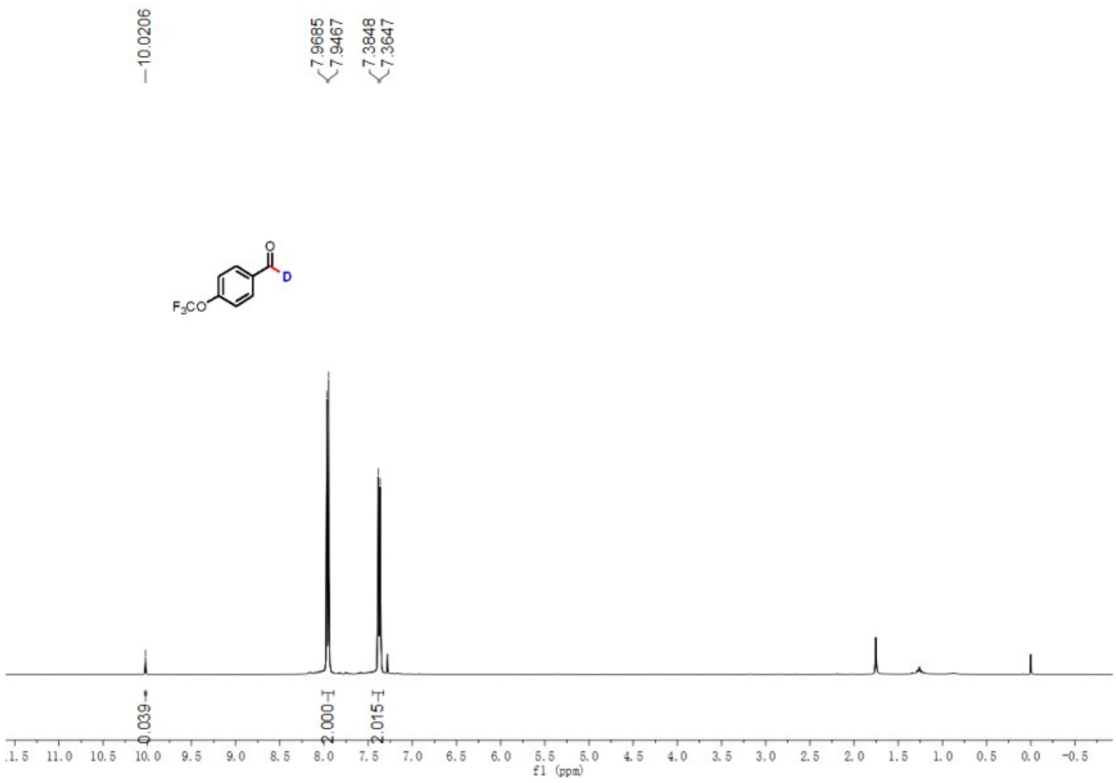

^{13}C NMR spectrum of compound **10n**

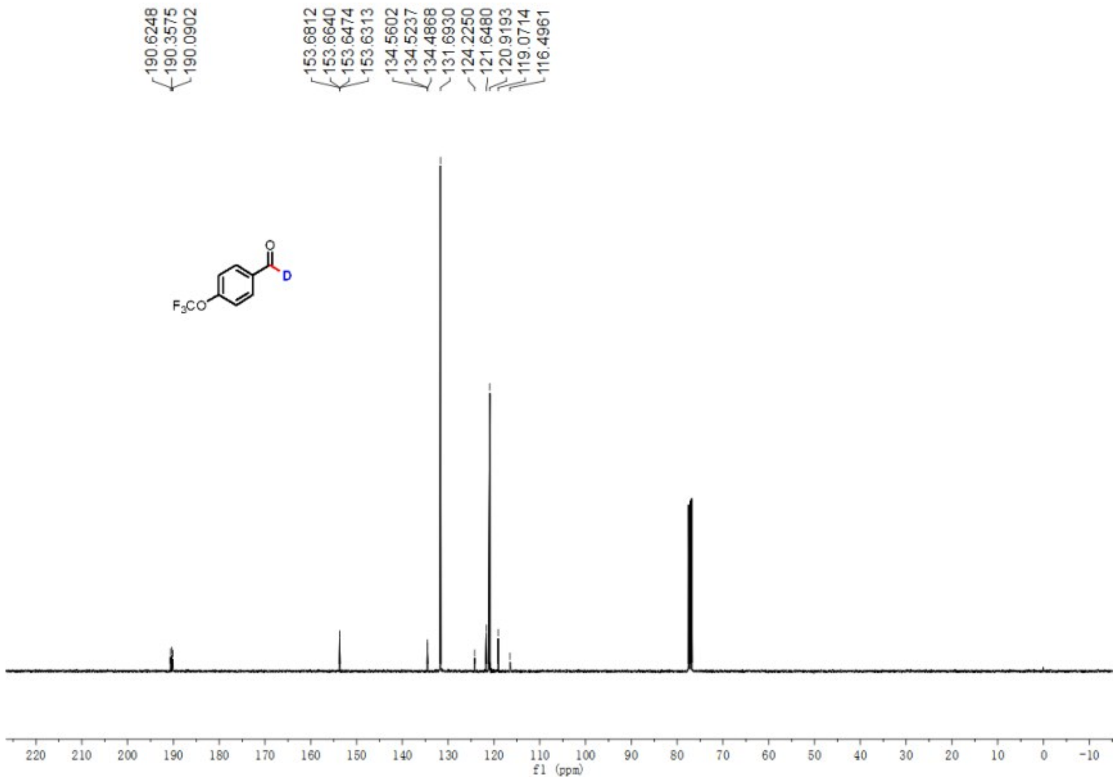

^{1}H NMR spectrum of compound **10o**

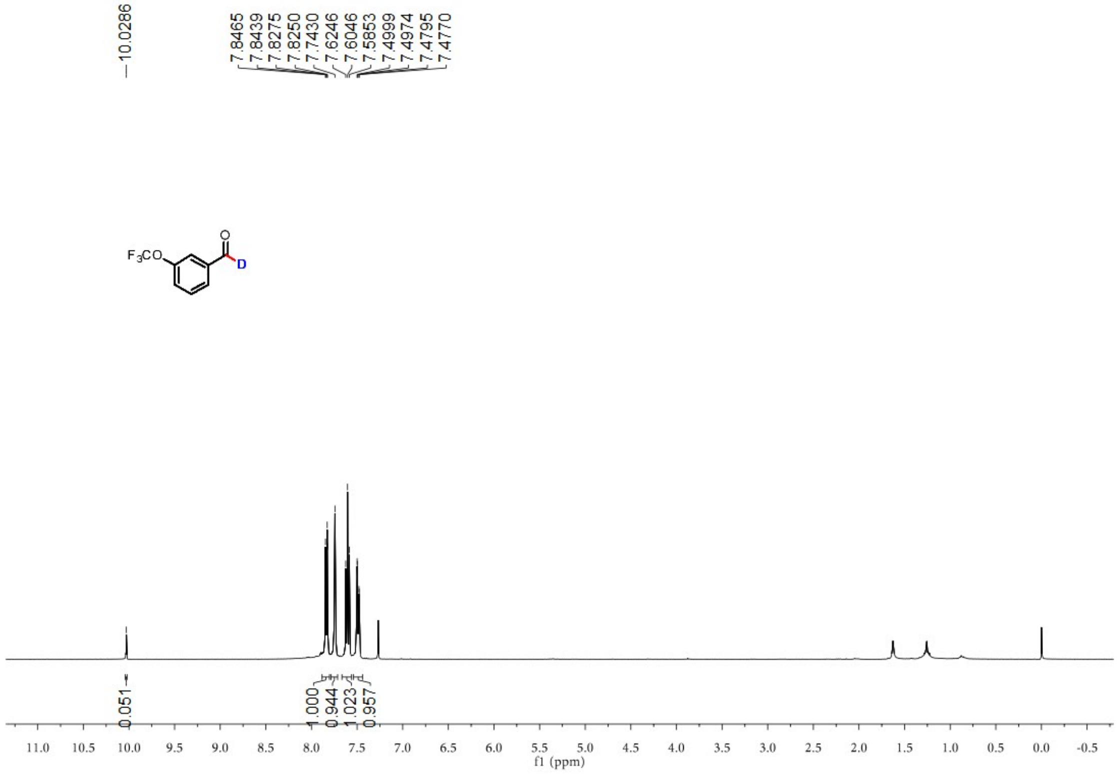

^{13}C NMR spectrum of compound **10o**

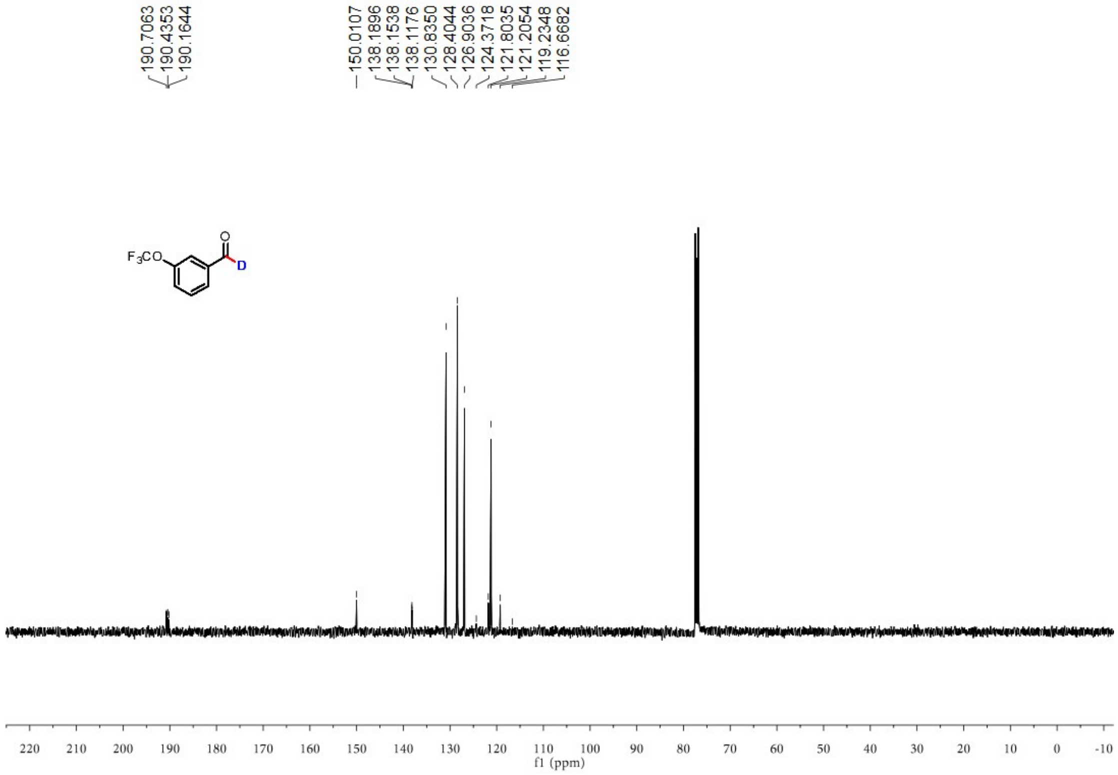

^{1}H NMR spectrum of compound **10p**

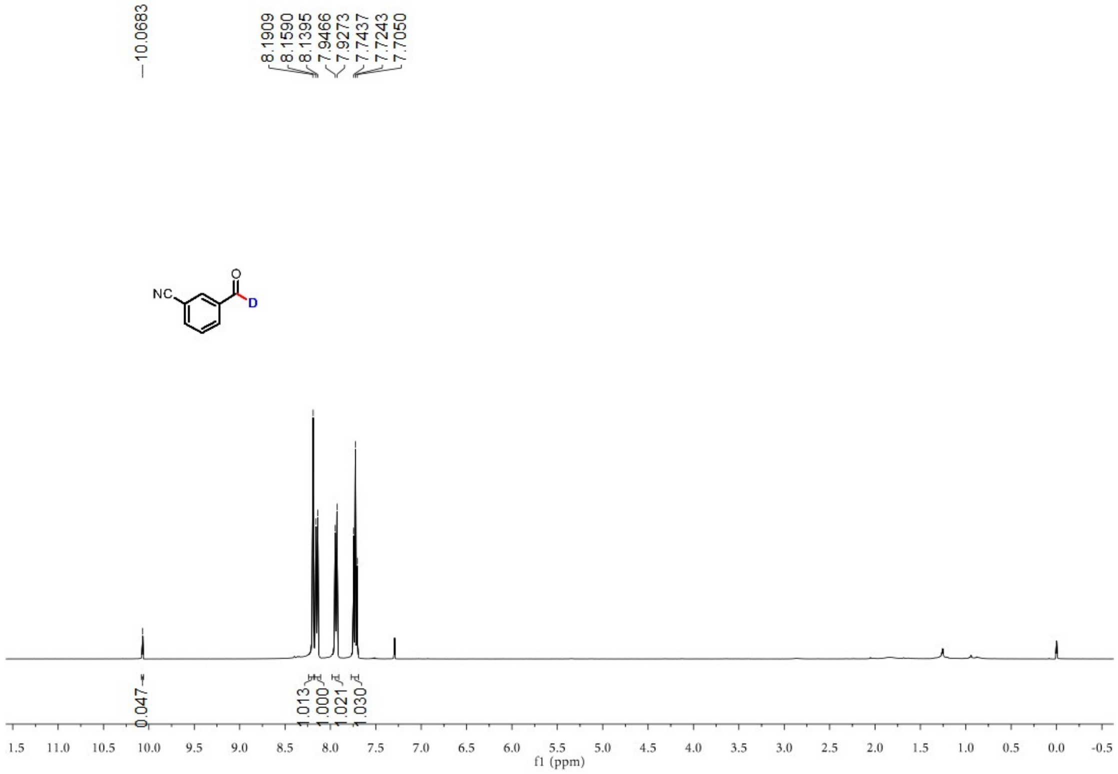

^{13}C NMR spectrum of compound **10p**

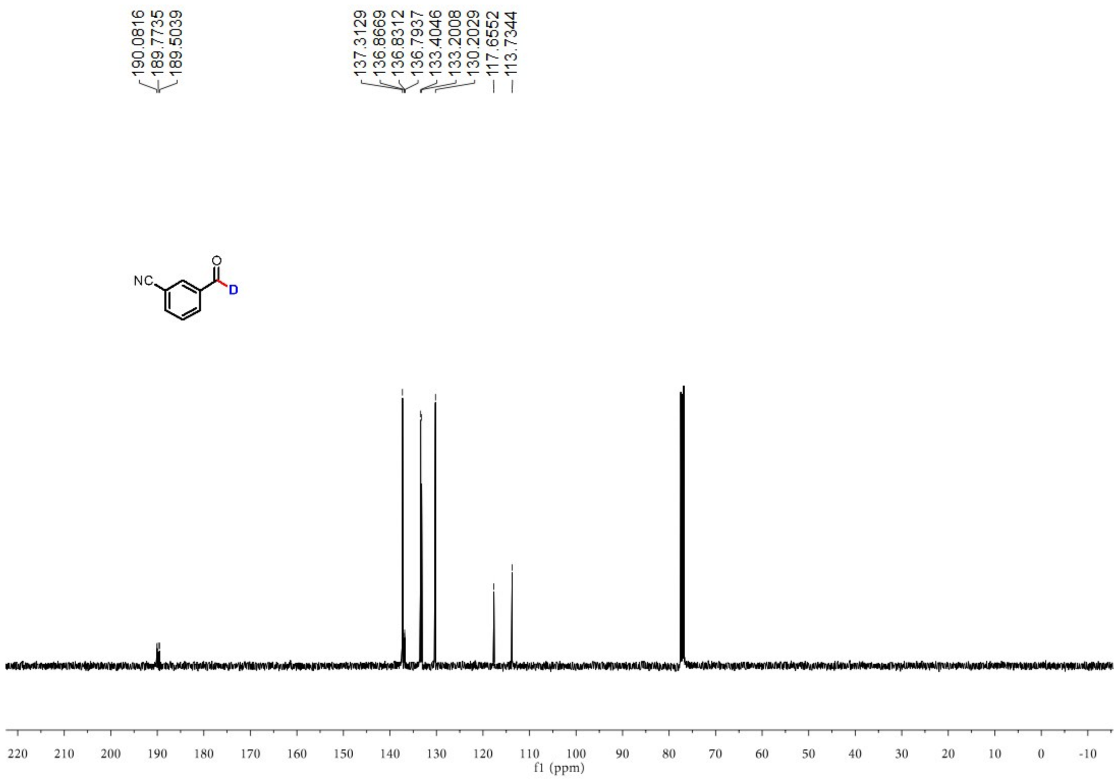

^{1}H NMR spectrum of compound **10q**

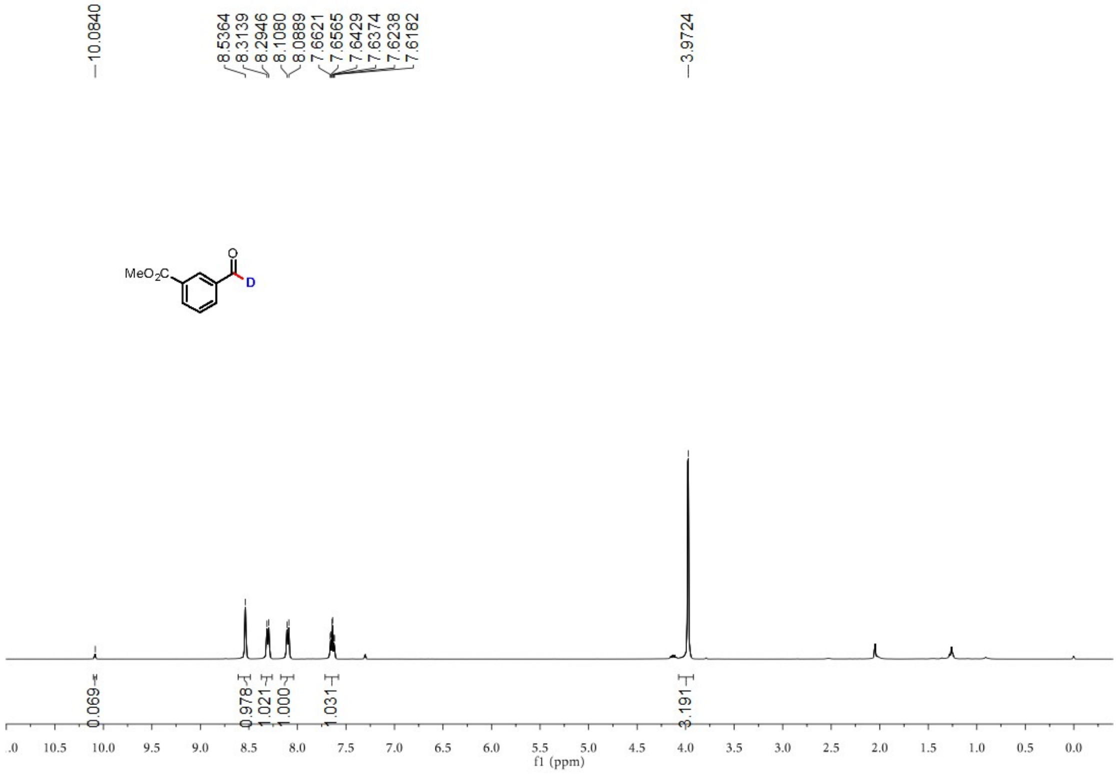

^{13}C NMR spectrum of compound **10q**

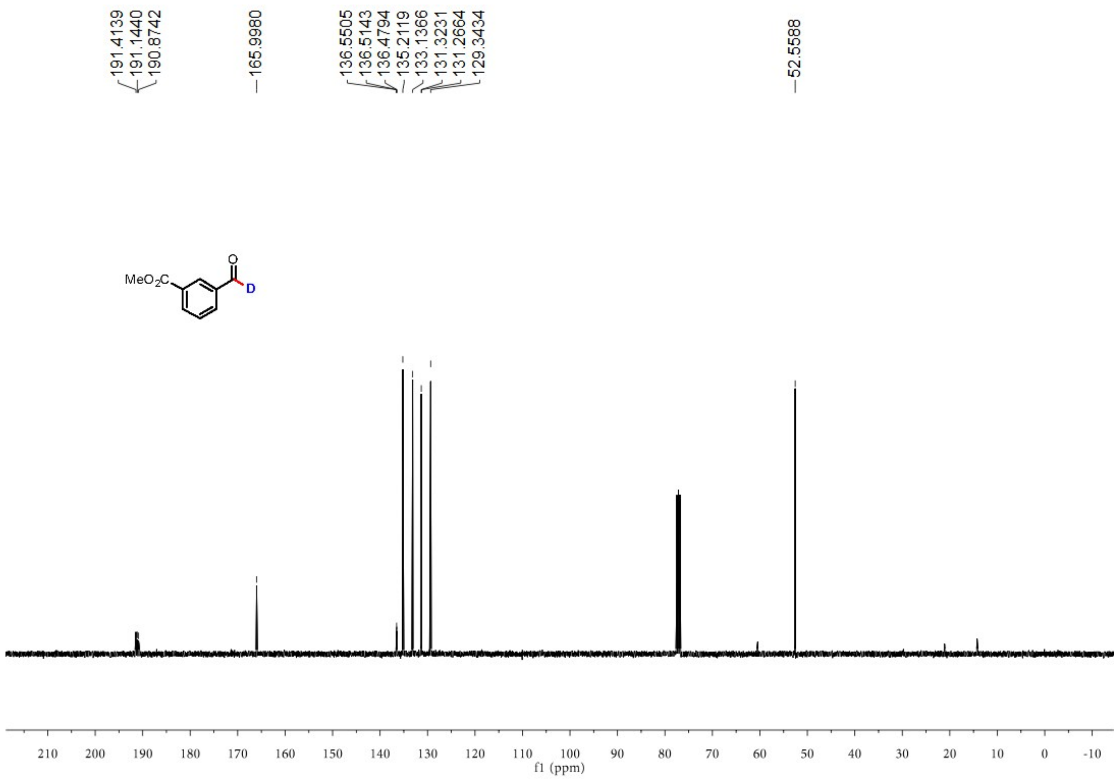

^{1}H NMR spectrum of compound **10r**

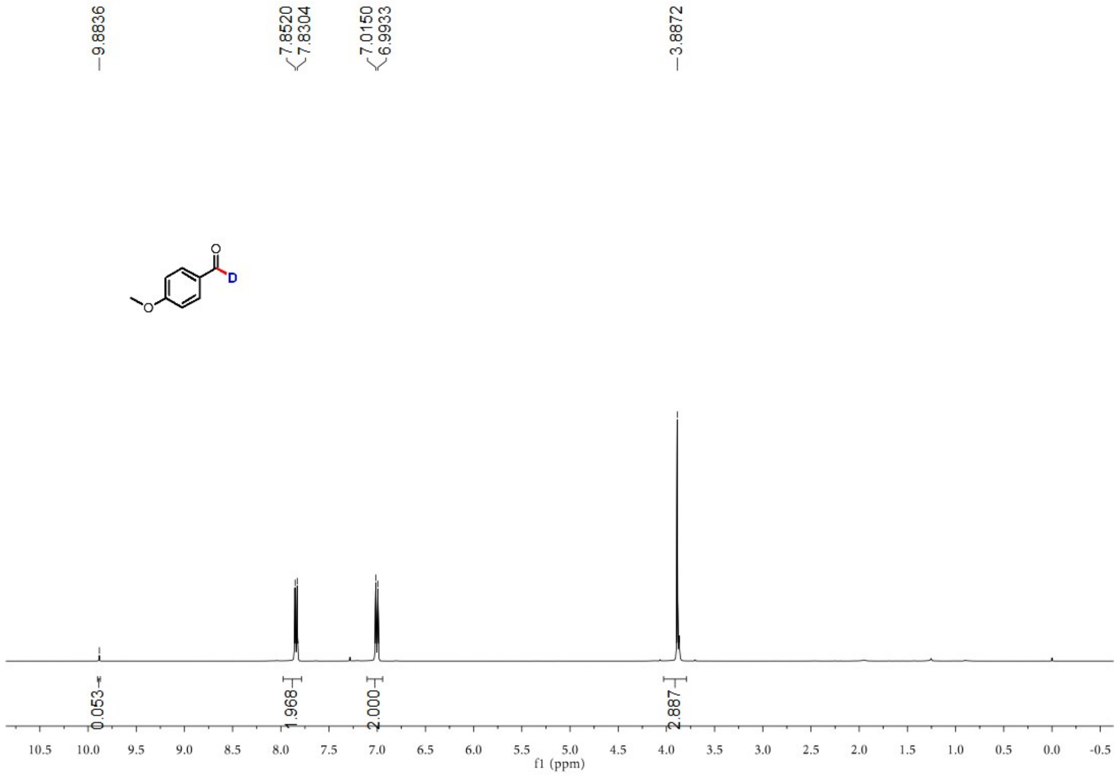

^{13}C NMR spectrum of compound **10r**

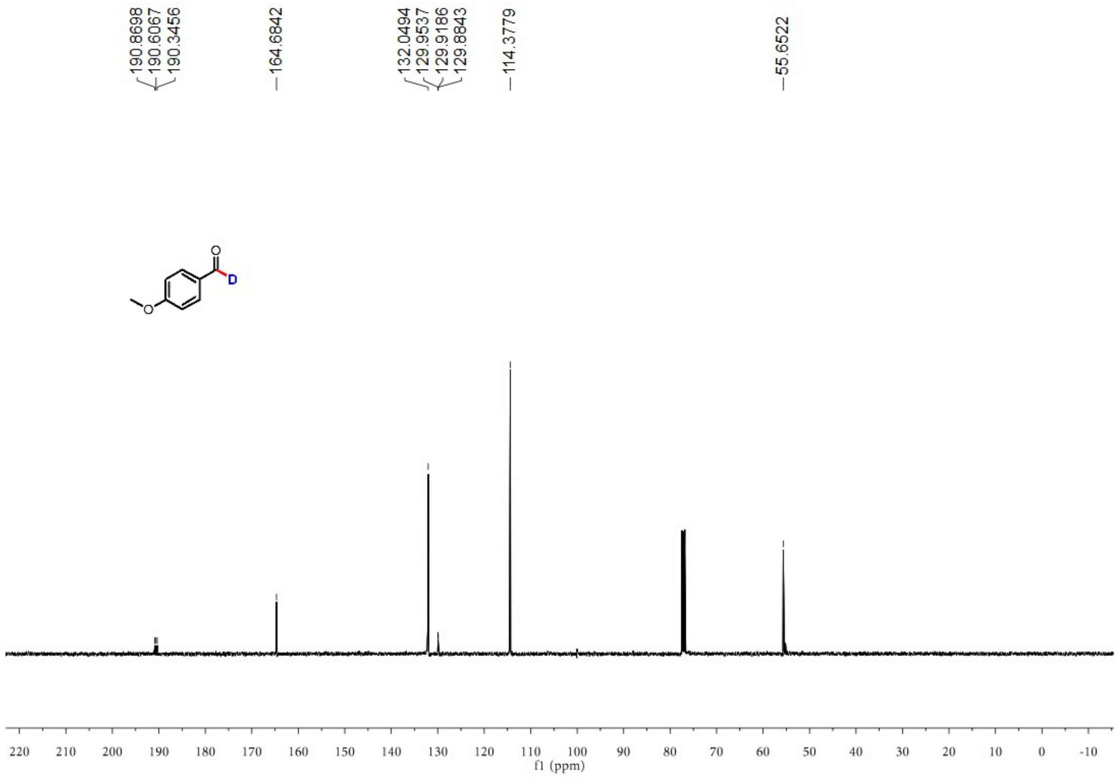

¹H NMR spectrum of compound **10s**

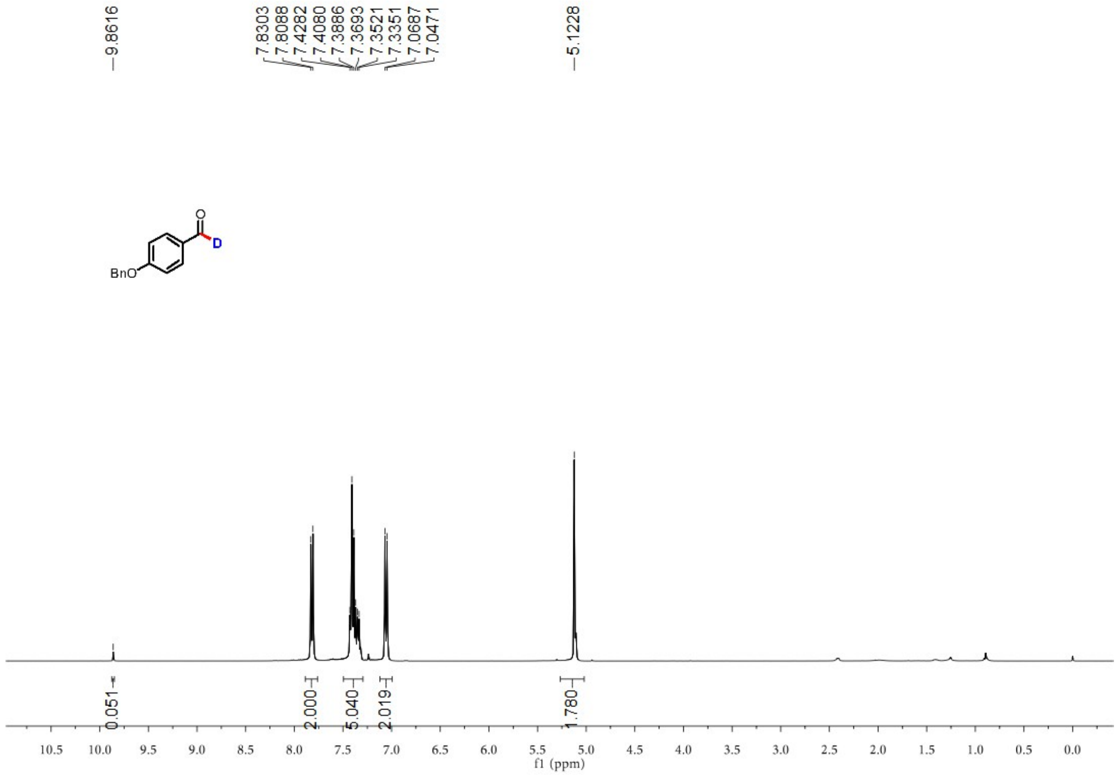

^{13}C NMR spectrum of compound **10s**

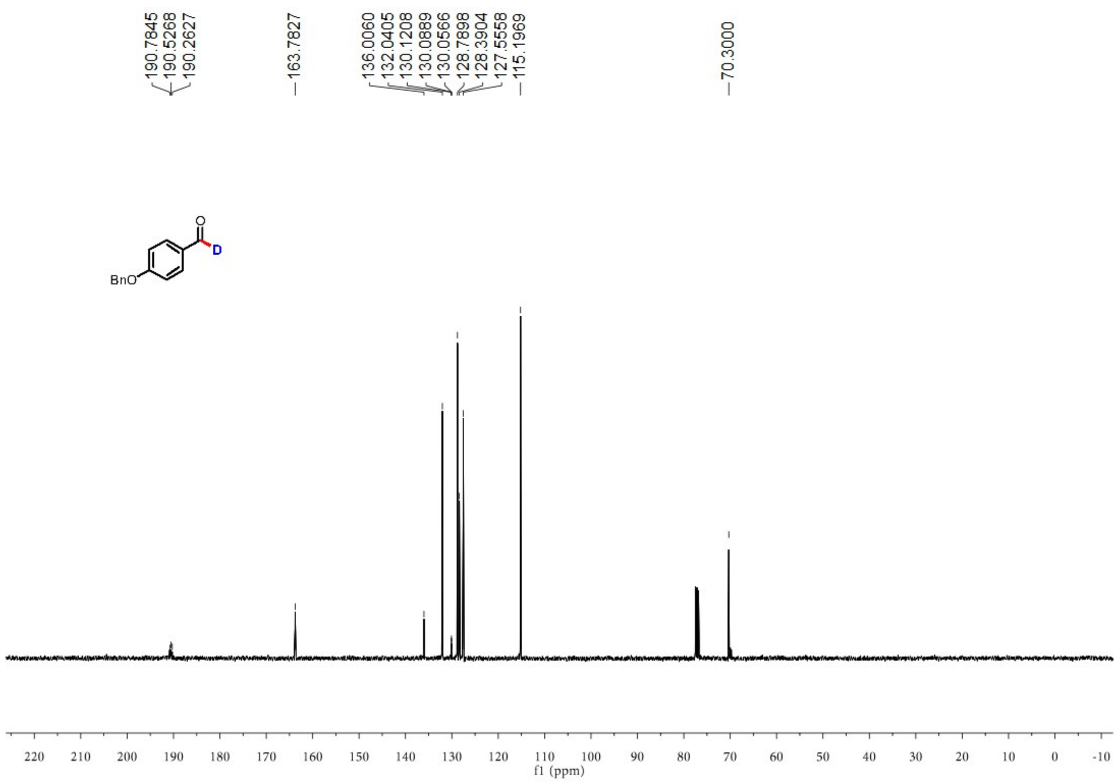

^{1}H NMR spectrum of compound **10t**

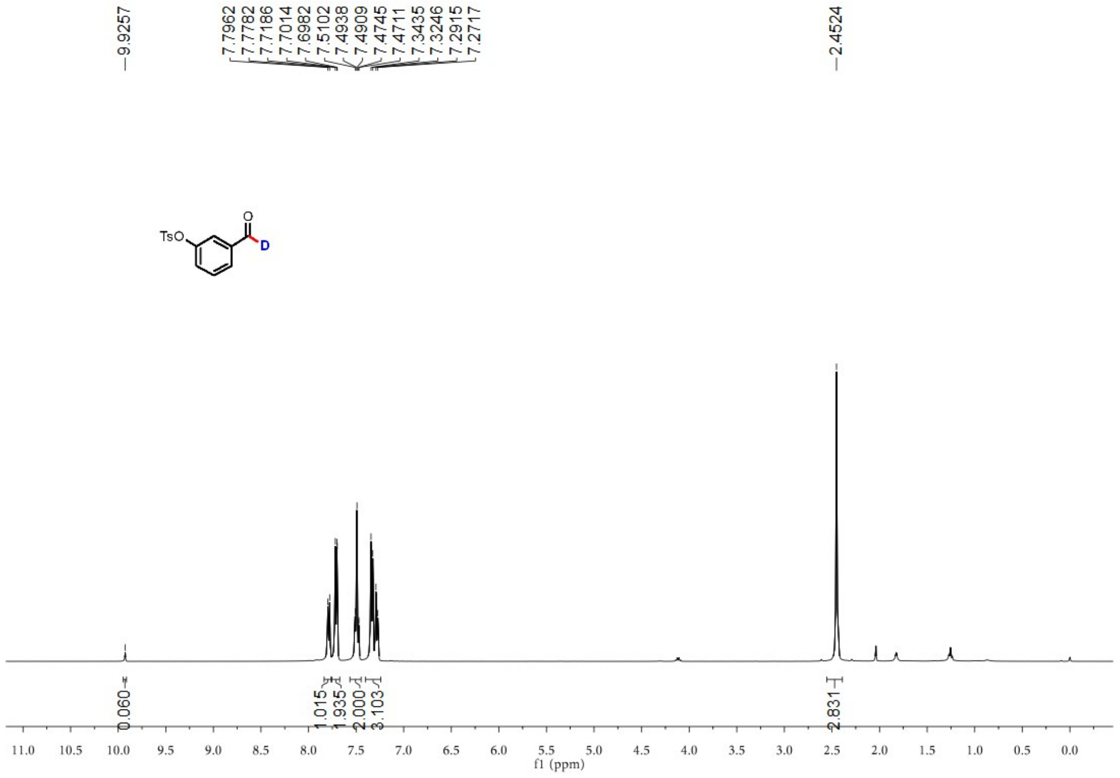

^{13}C NMR spectrum of compound **10t**

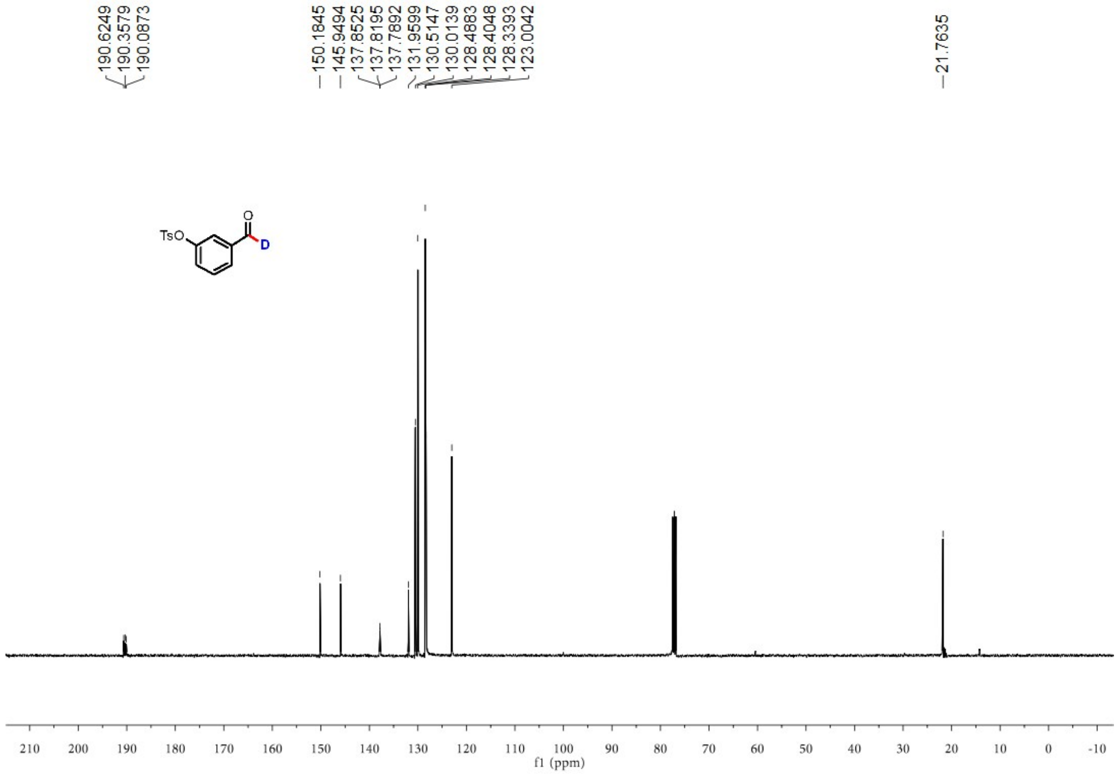

^{1}H NMR spectrum of compound **10u**

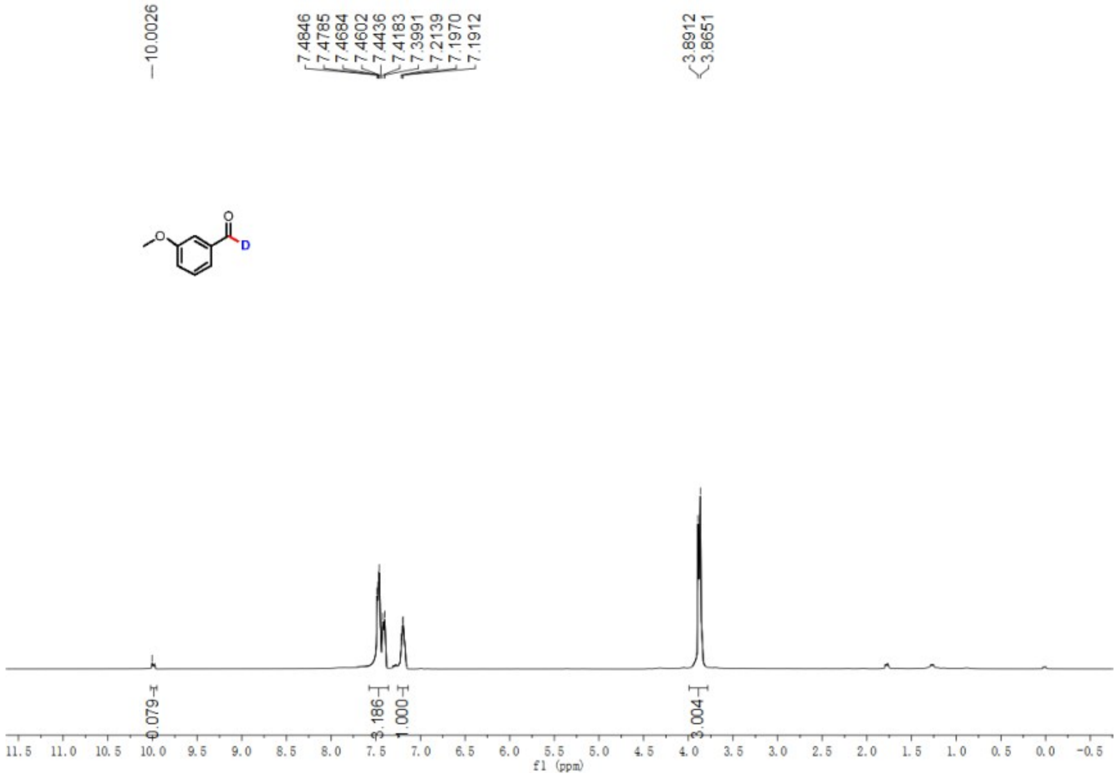

^{13}C NMR spectrum of compound **10u**

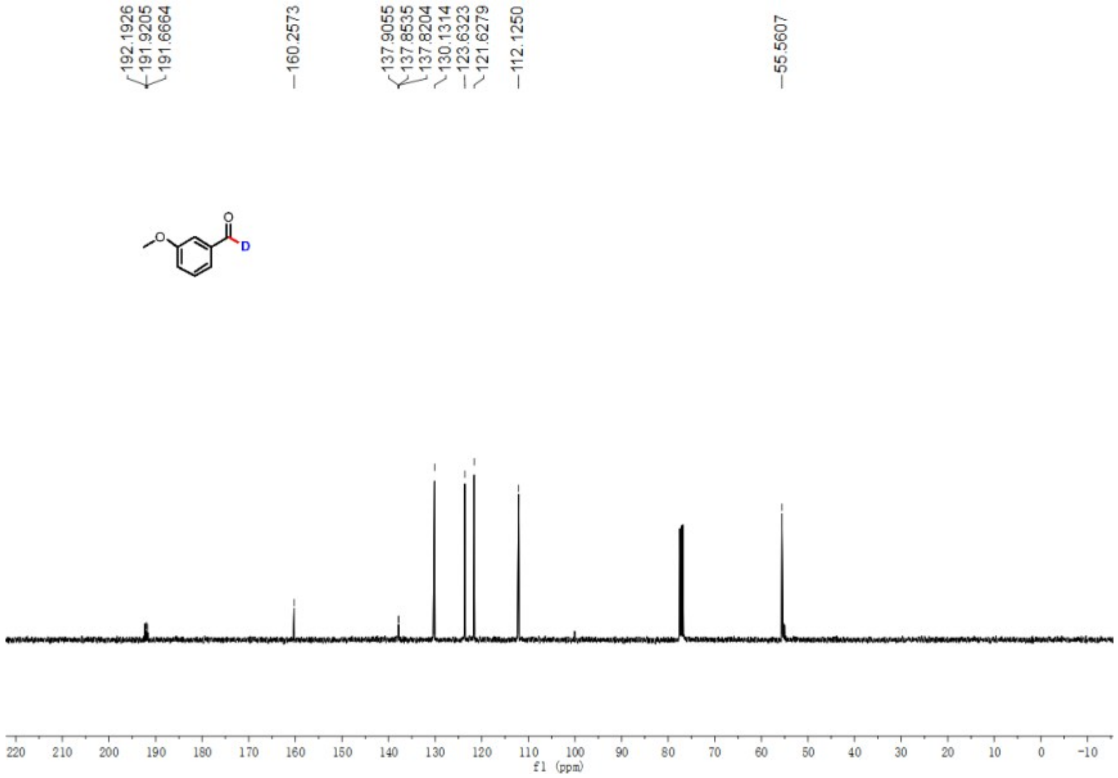

^{1}H NMR spectrum of compound **10v**

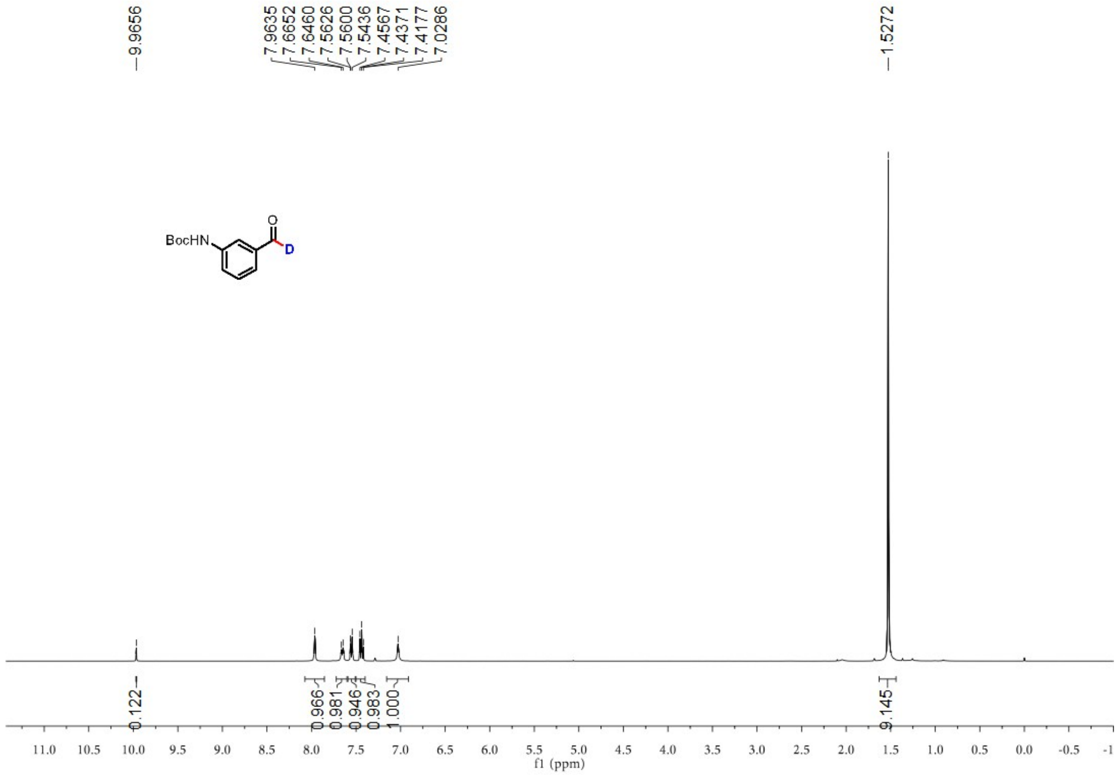

^{13}C NMR spectrum of compound **10v**

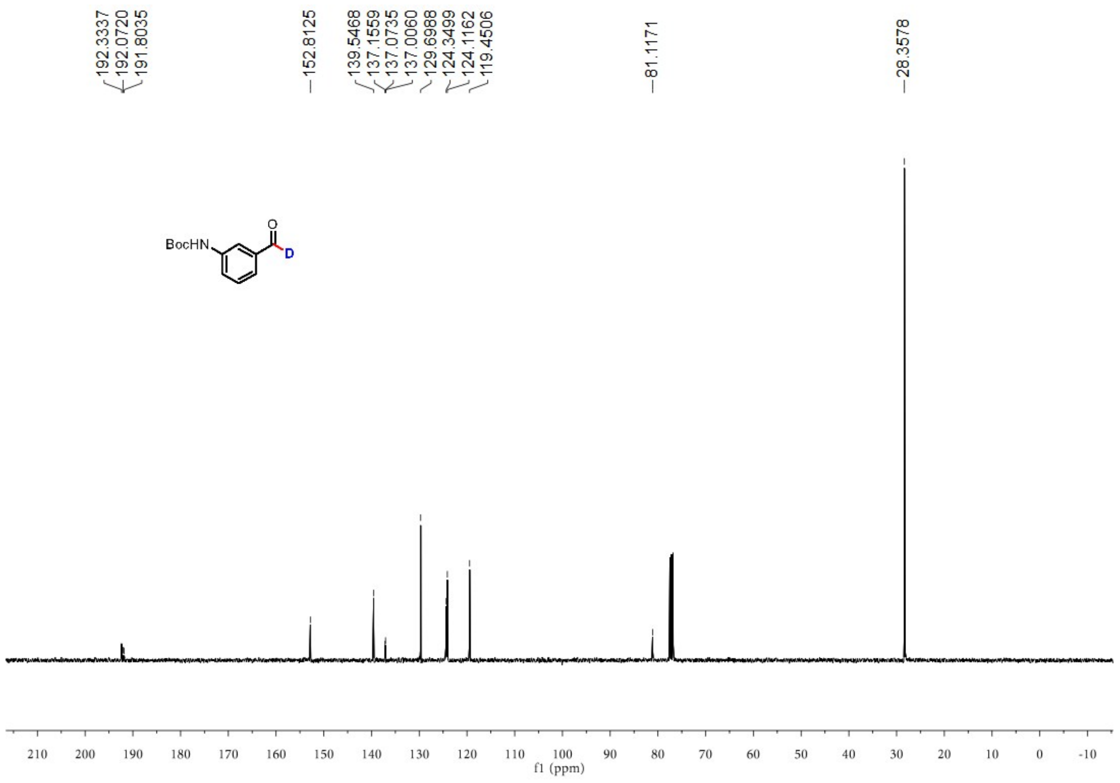

^{1}H NMR spectrum of compound **10w**

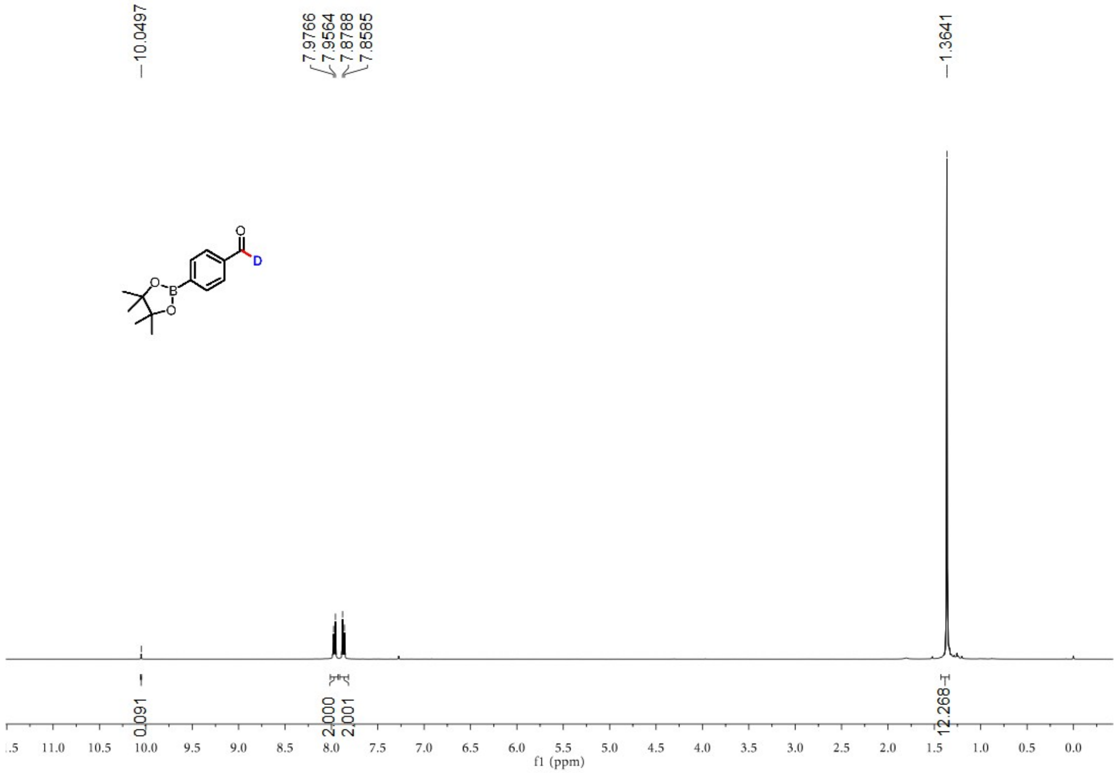

^{13}C NMR spectrum of compound **10w**

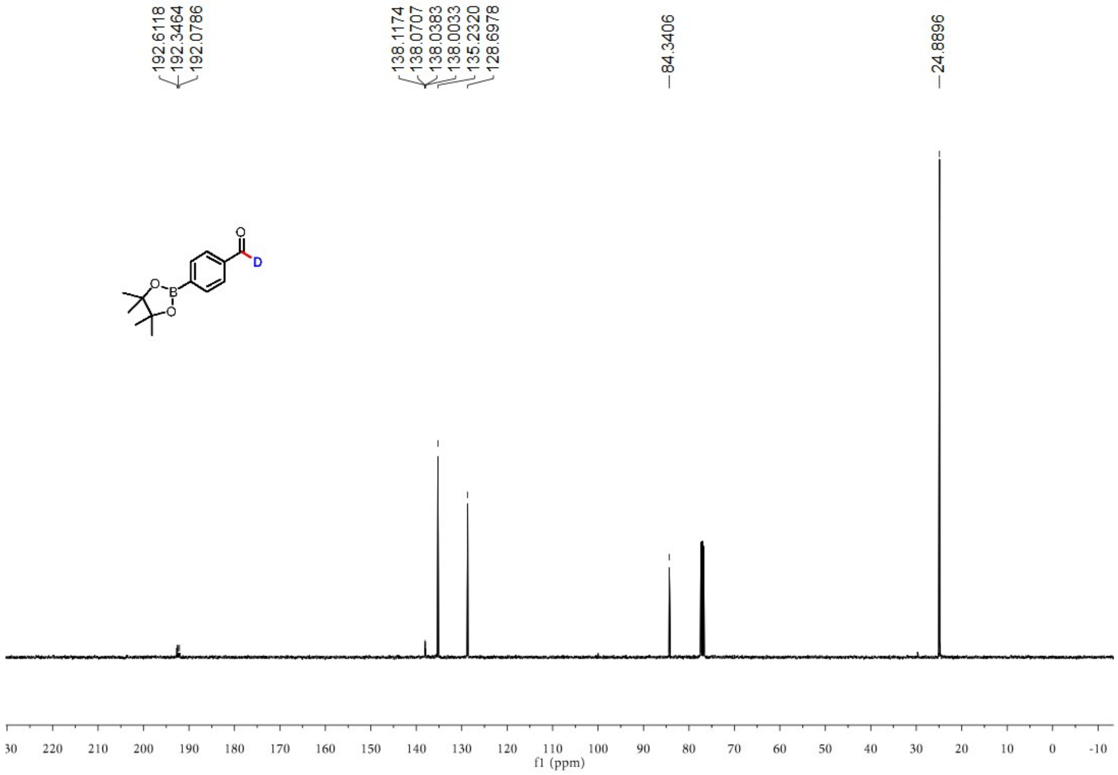

^{1}H NMR spectrum of compound **10x**

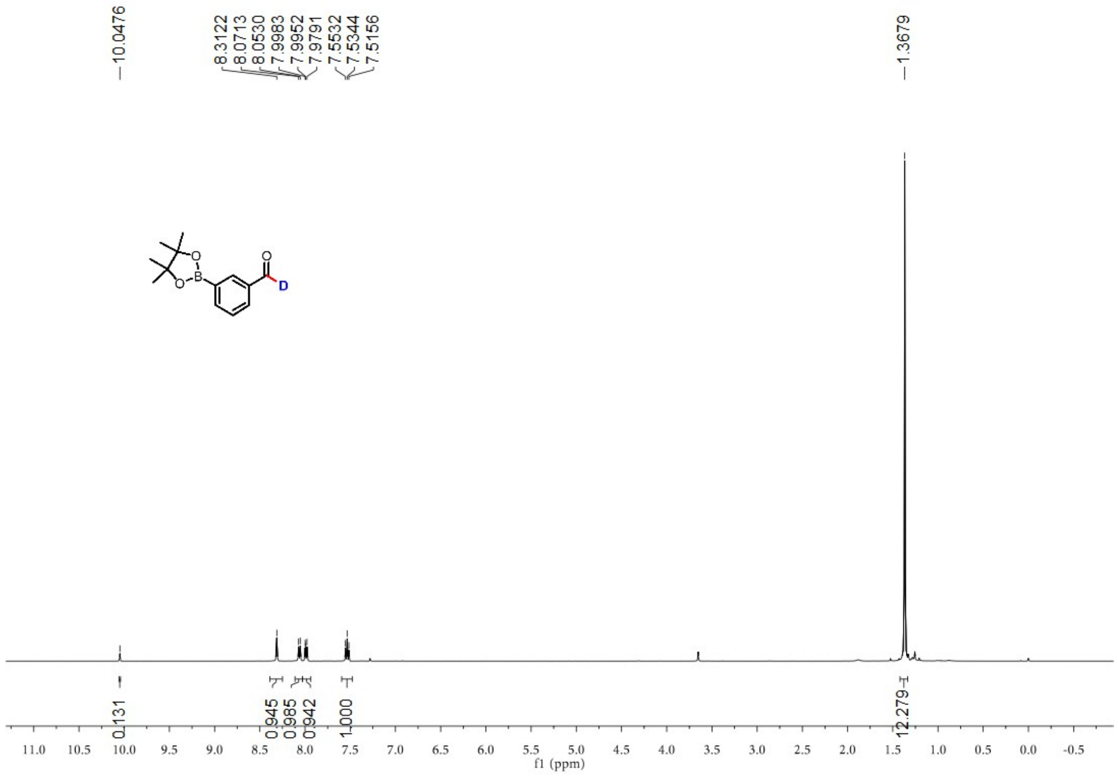

^{13}C NMR spectrum of compound **10x**

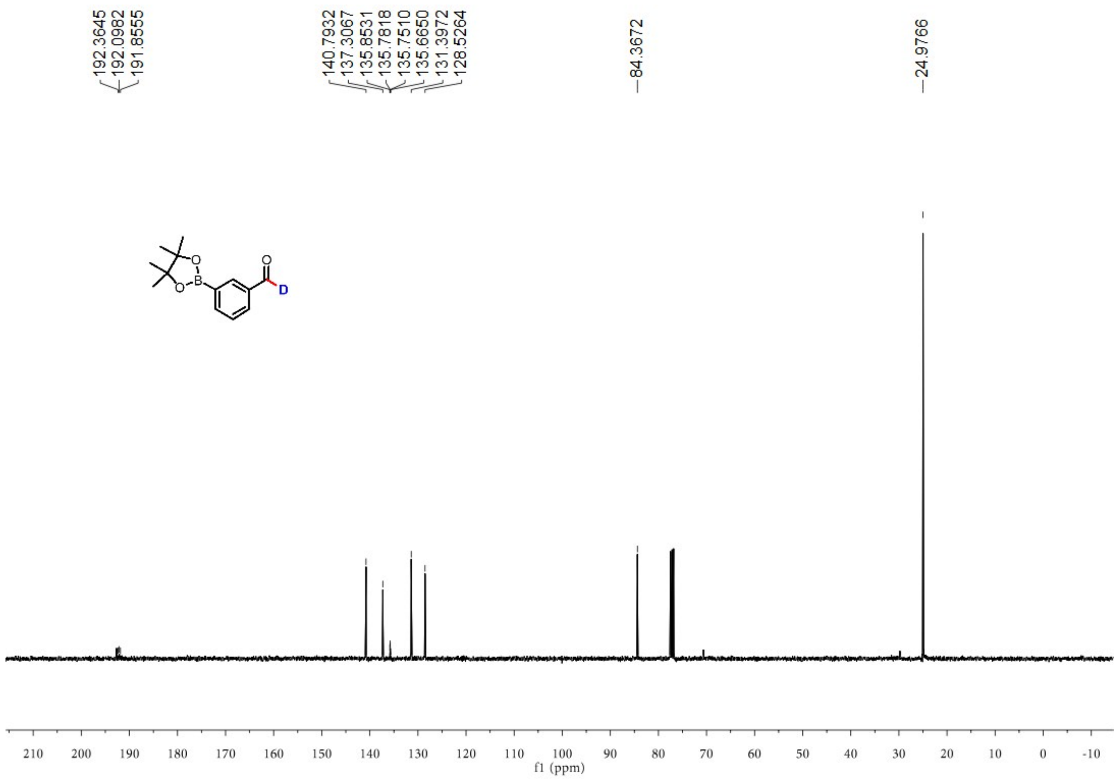

^{1}H NMR spectrum of compound **10y**

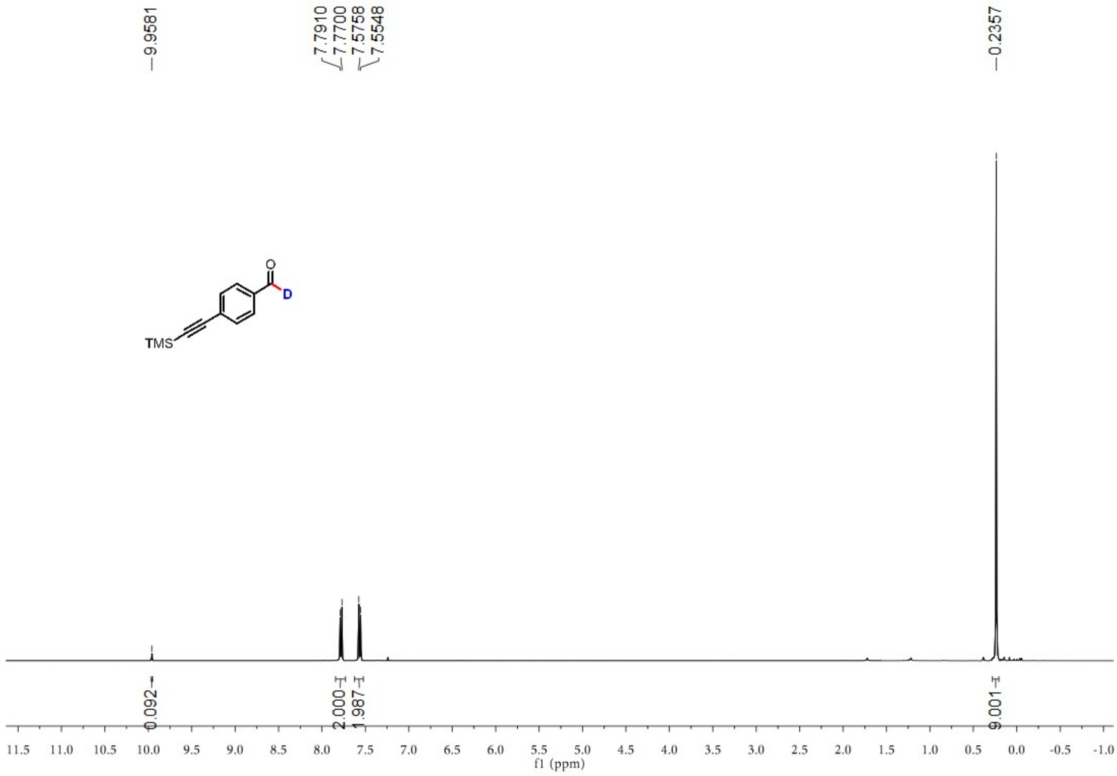

^{13}C NMR spectrum of compound **10y**

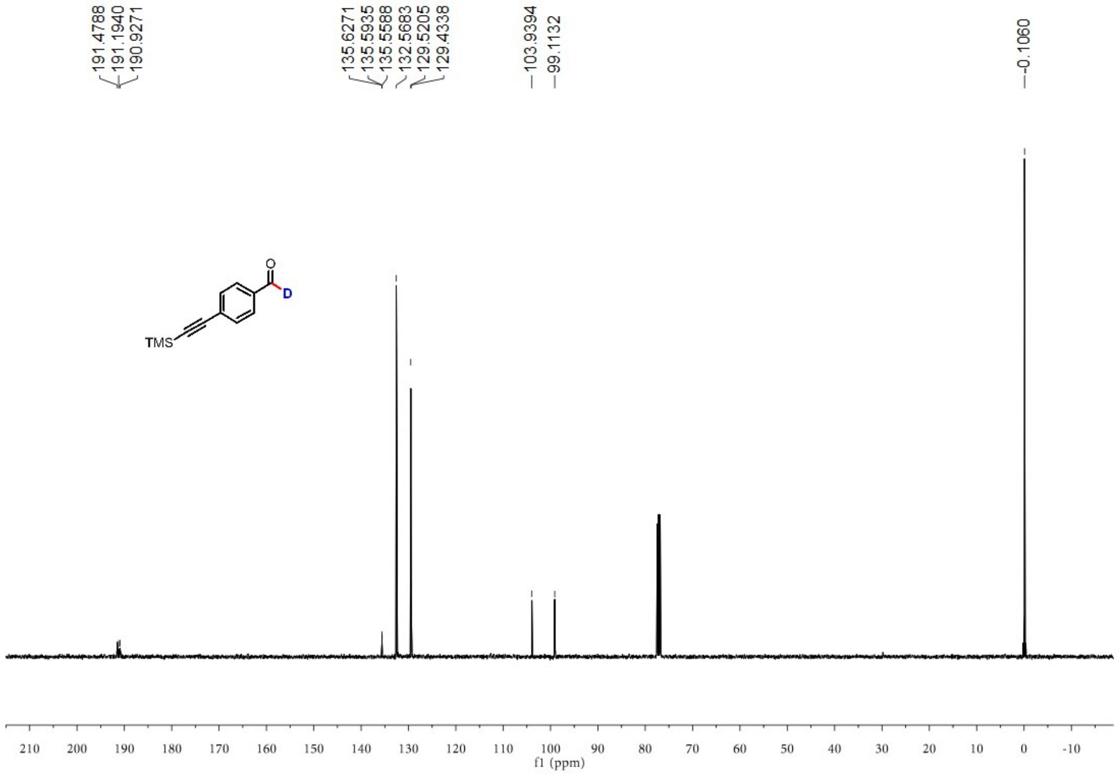

^{1}H NMR spectrum of compound **10z**

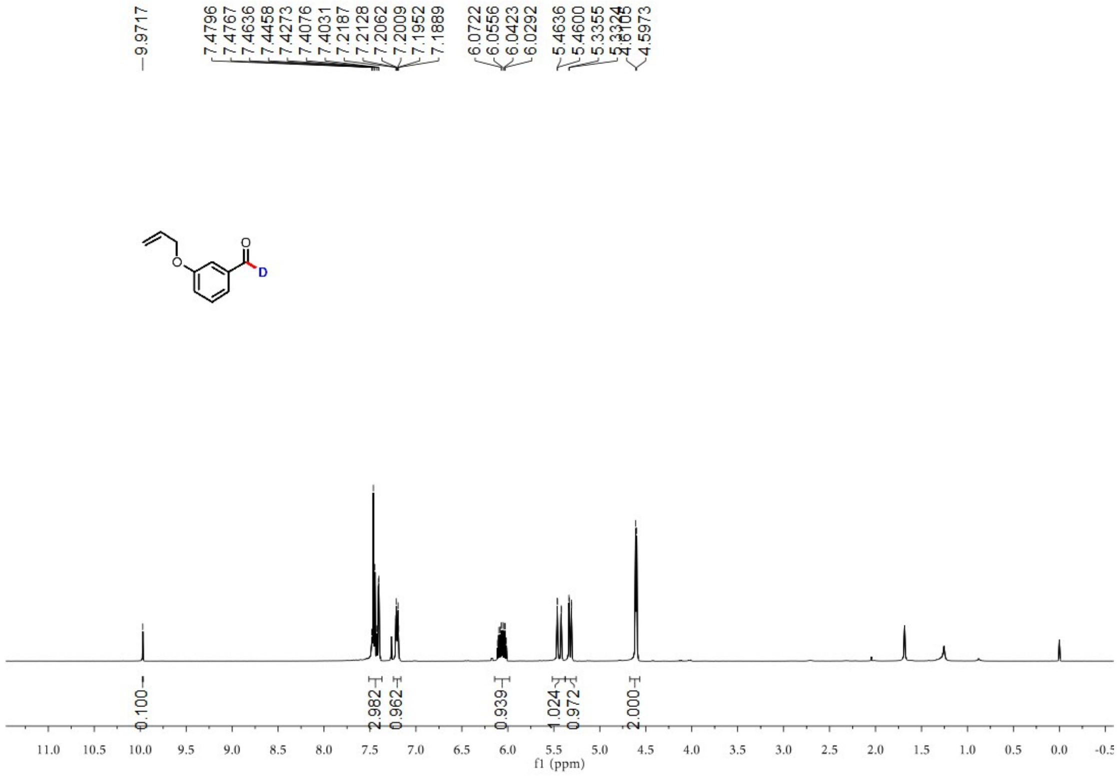

^{13}C NMR spectrum of compound **10z**

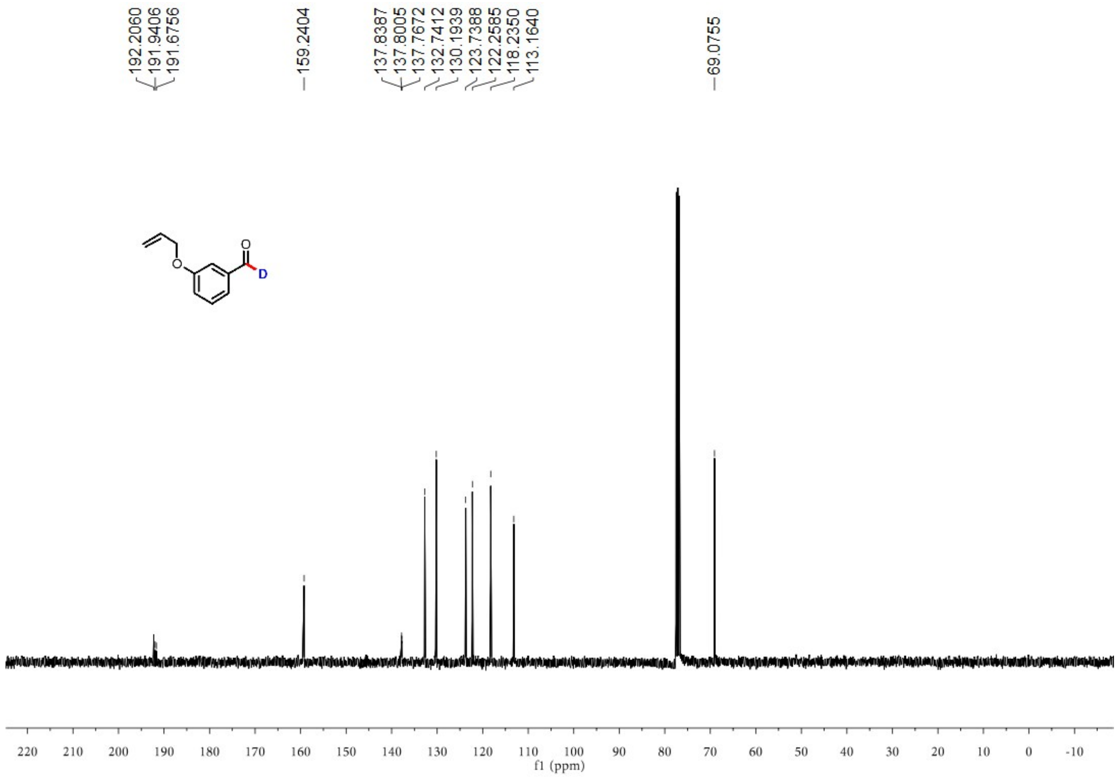

^{1}H NMR spectrum of compound **10aa**

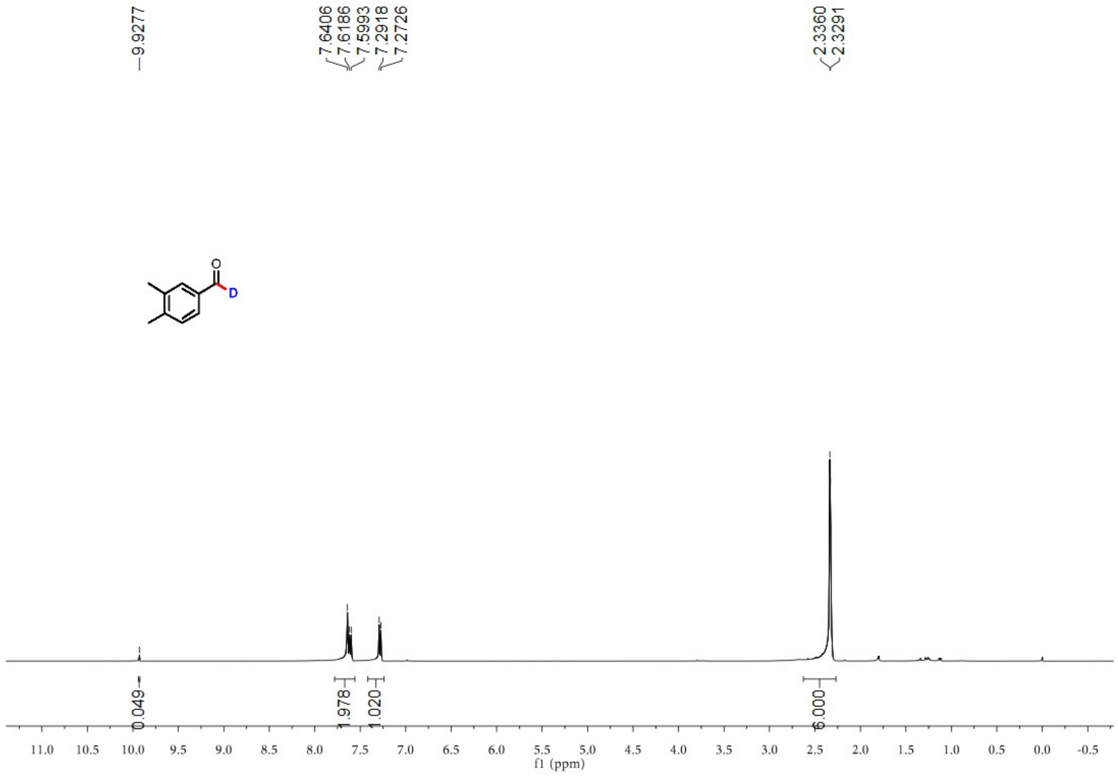

^{13}C NMR spectrum of compound **10aa**

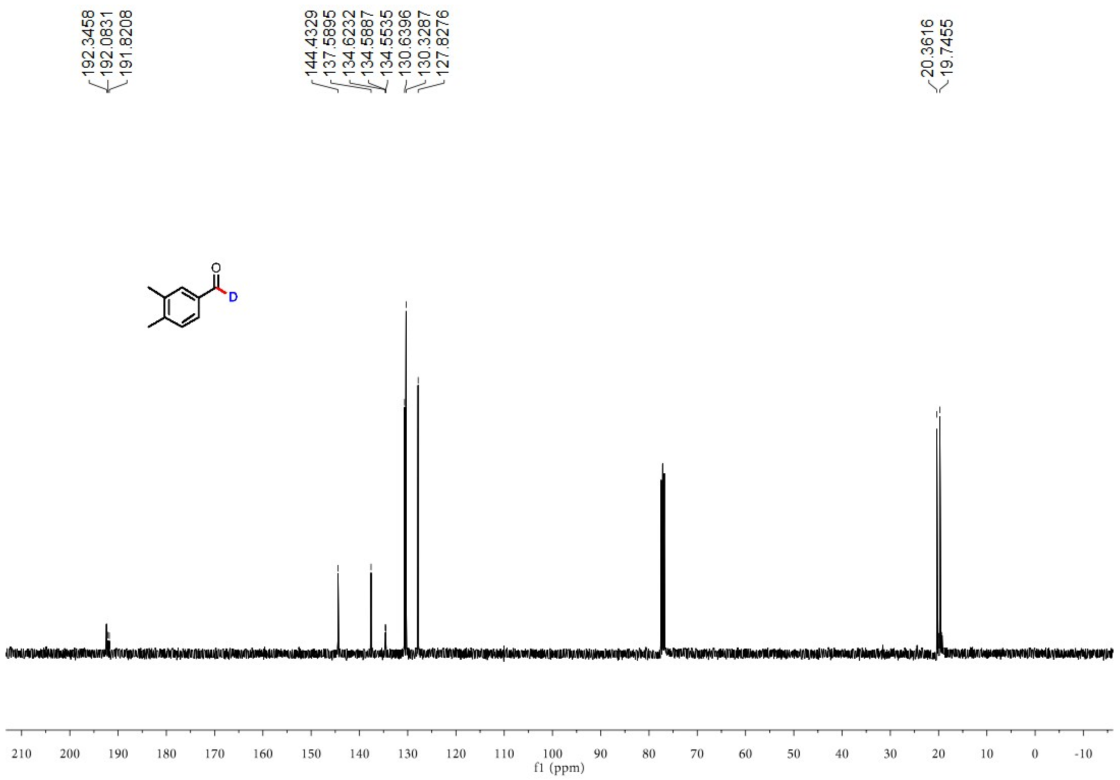

^{1}H NMR spectrum of compound **10bb**

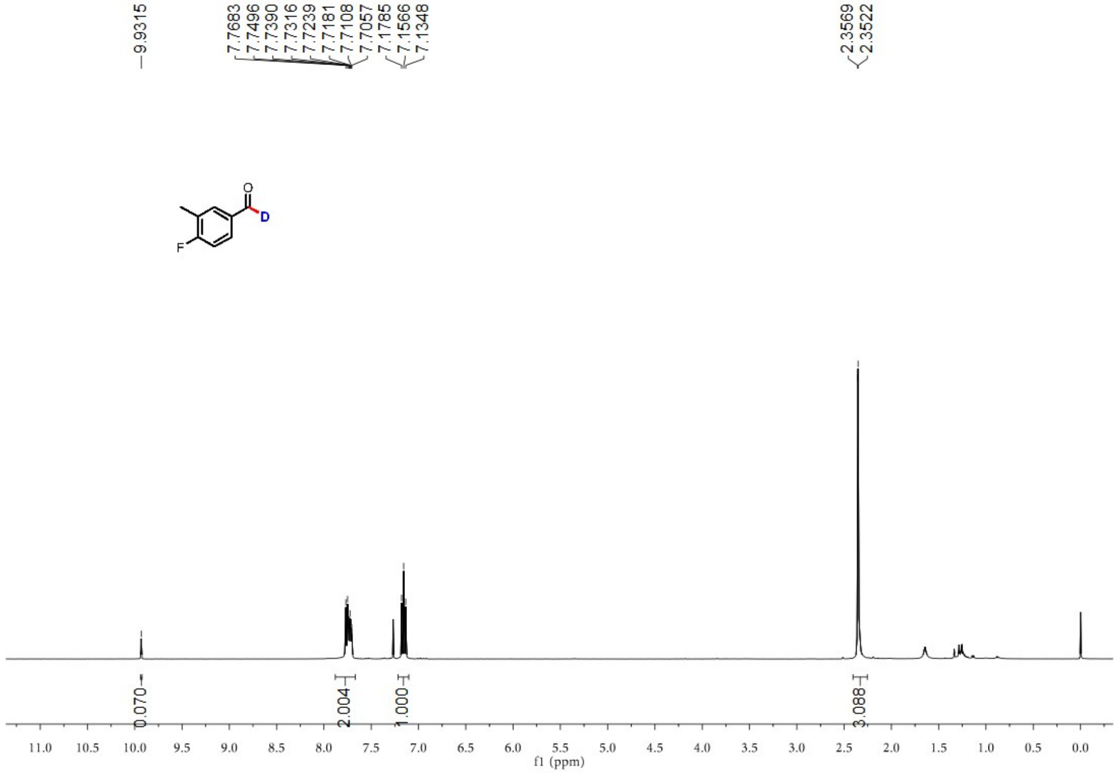

^{13}C NMR spectrum of compound **10bb**

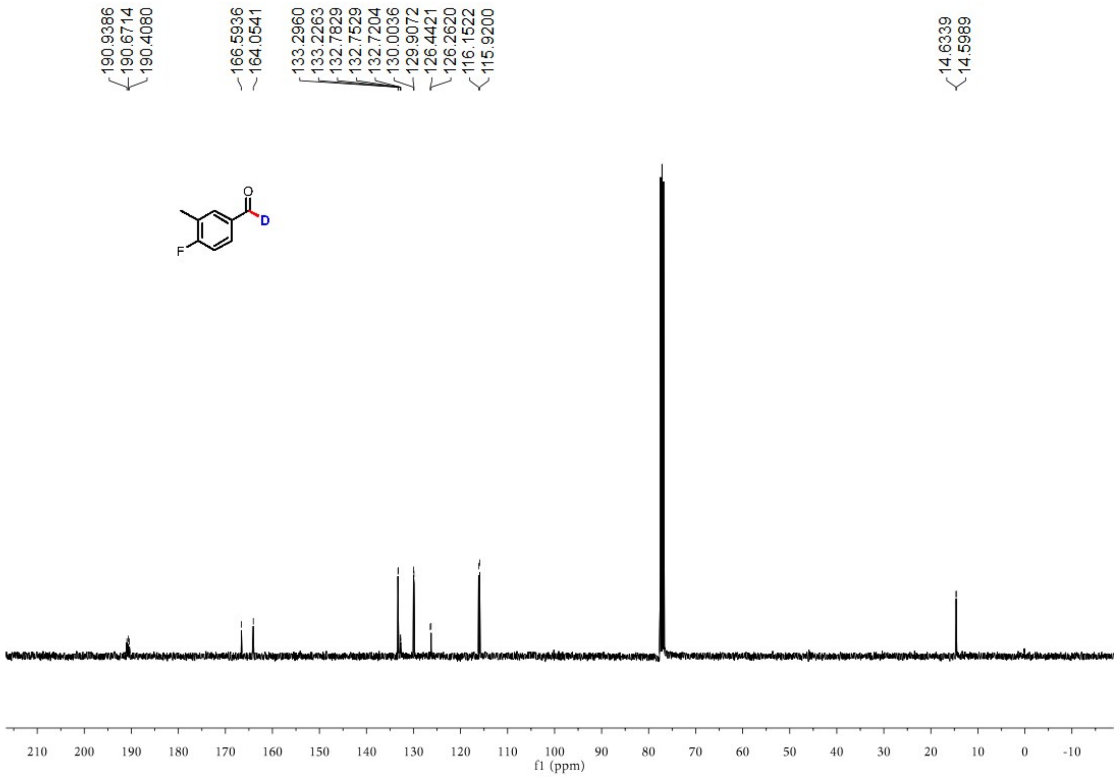

^{1}H NMR spectrum of compound **10cc**

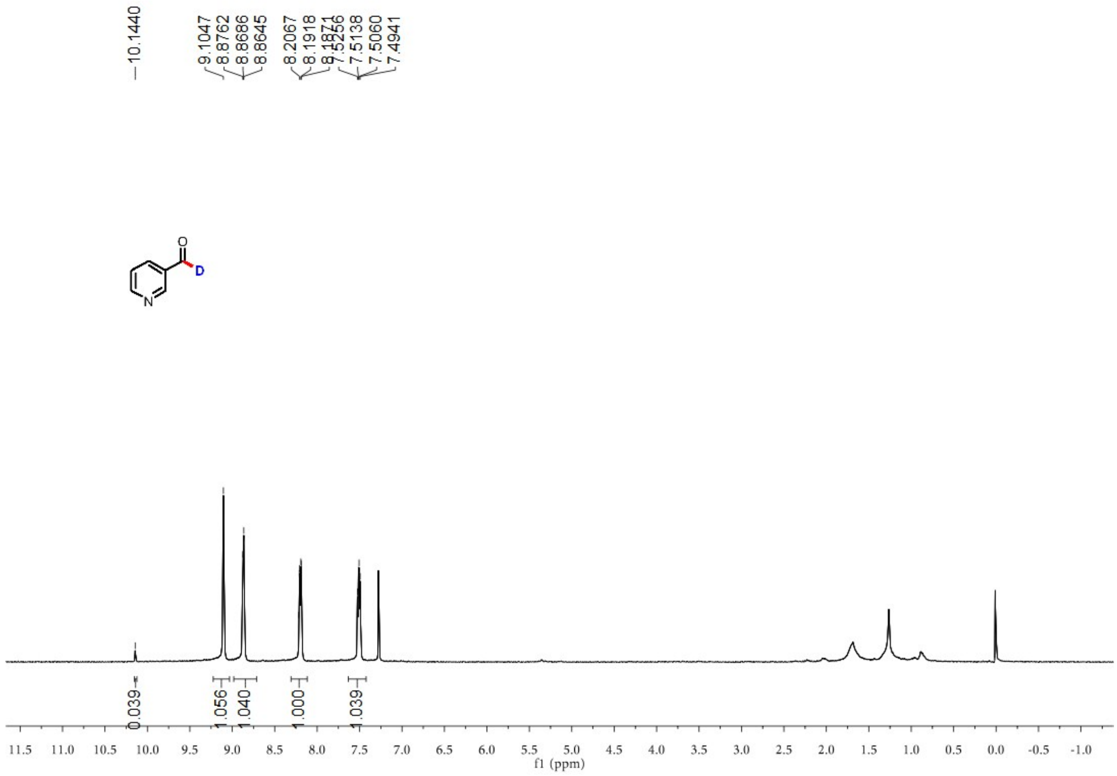

^{13}C NMR spectrum of compound **10cc**

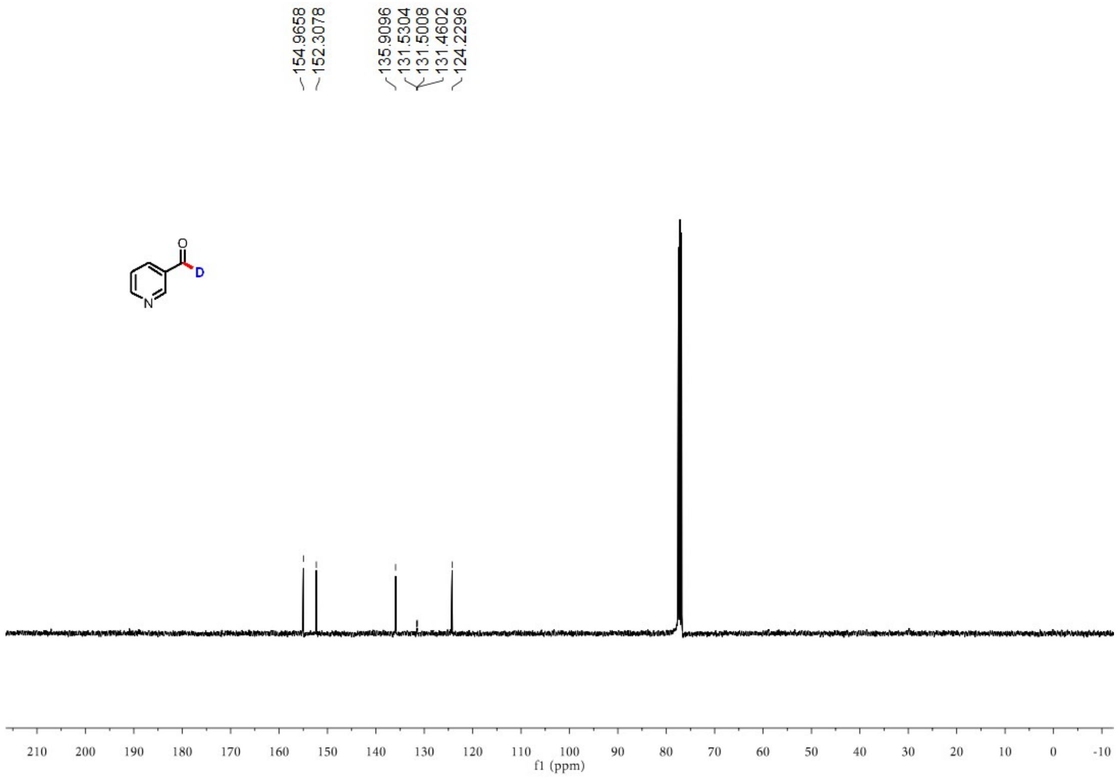

^{1}H NMR spectrum of compound **10dd**

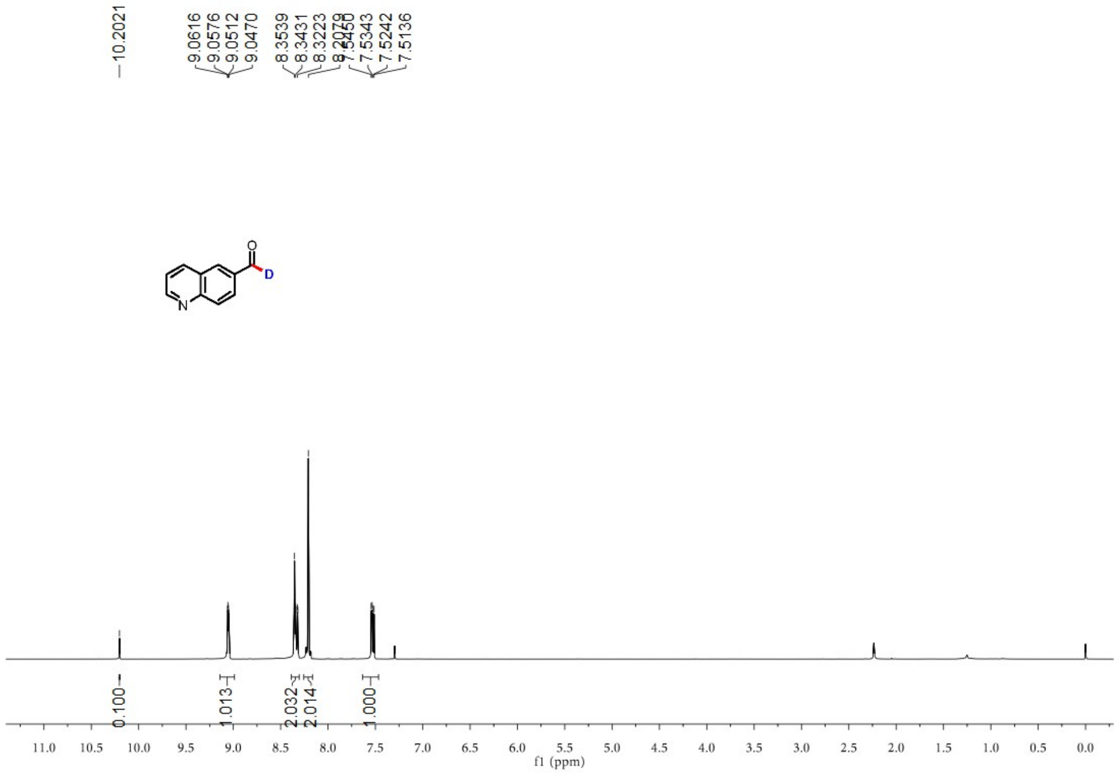

^{13}C NMR spectrum of compound **10dd**

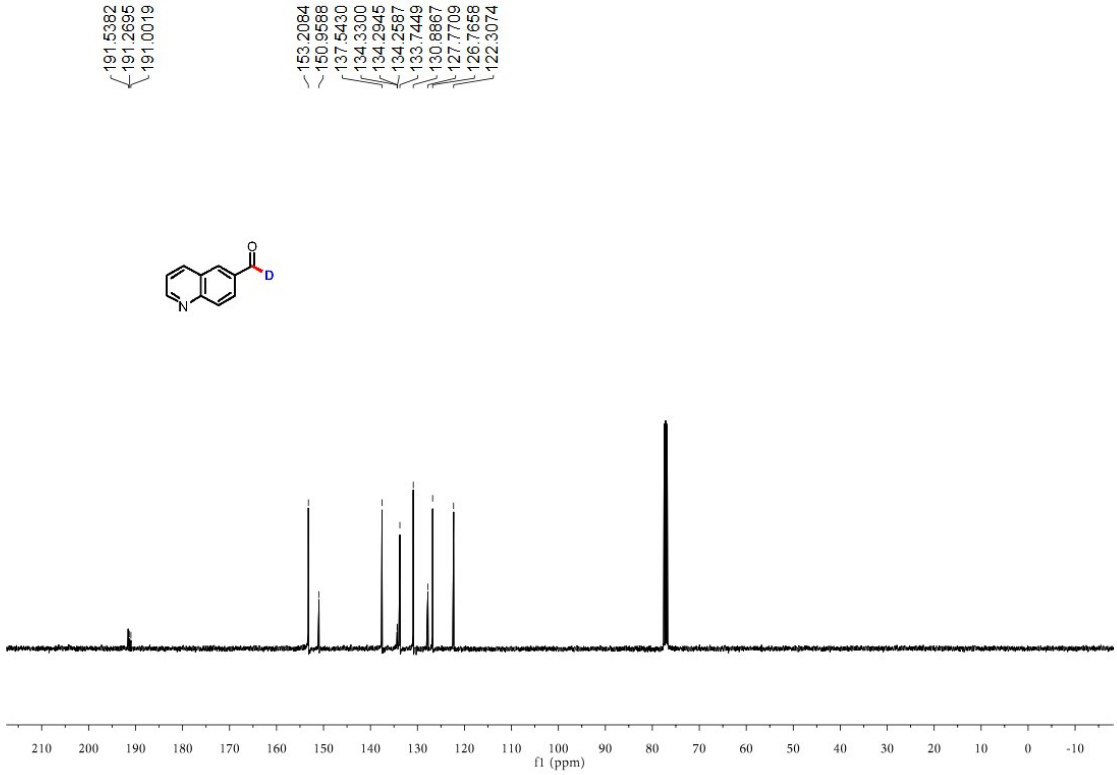

^{1}H NMR spectrum of compound **10ee**

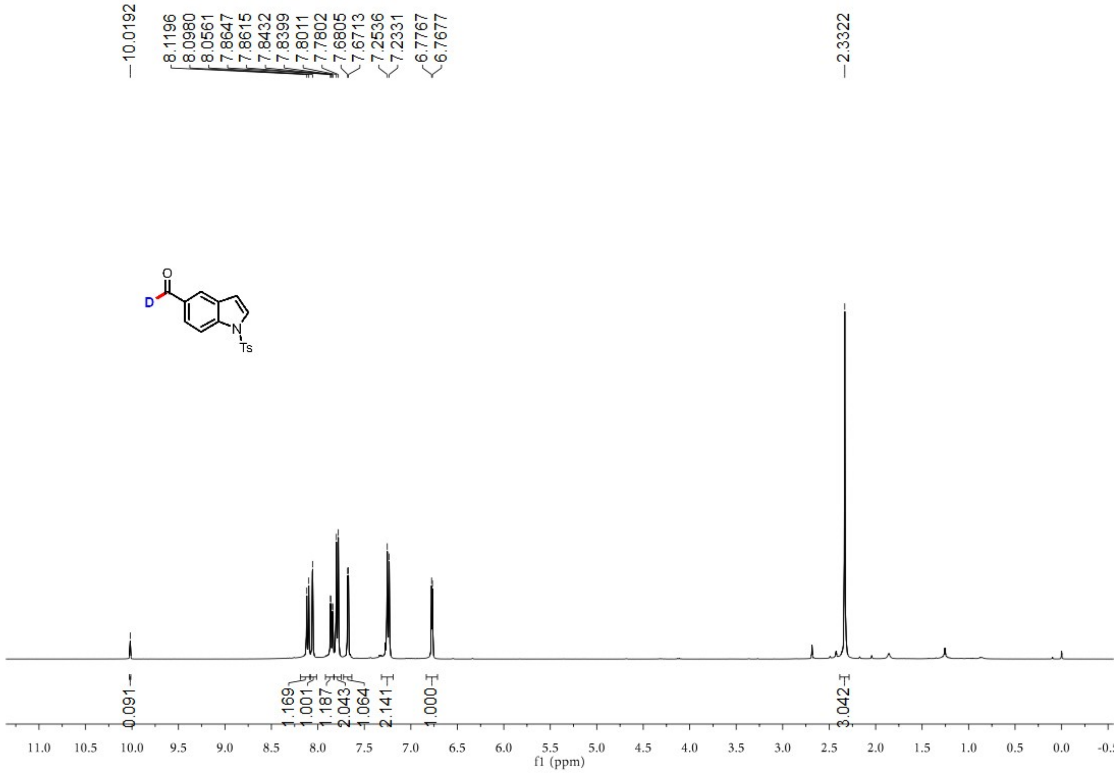

^{13}C NMR spectrum of compound **10ee**

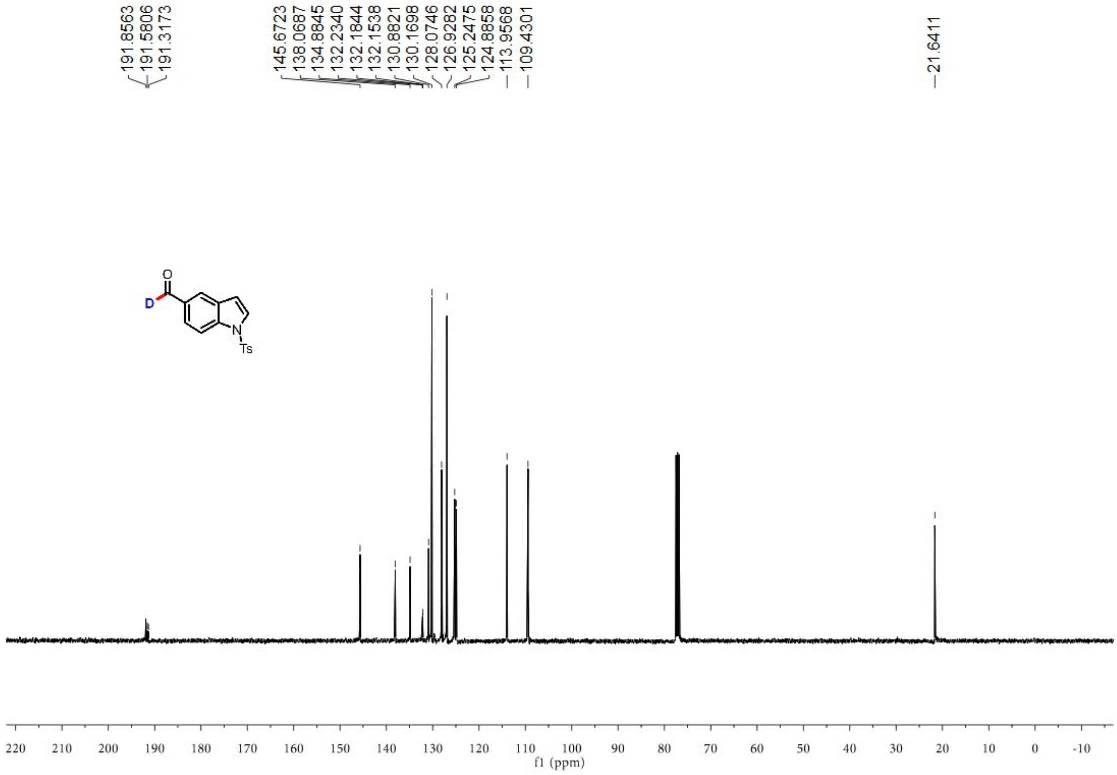

^{1}H NMR spectrum of compound **10ff**

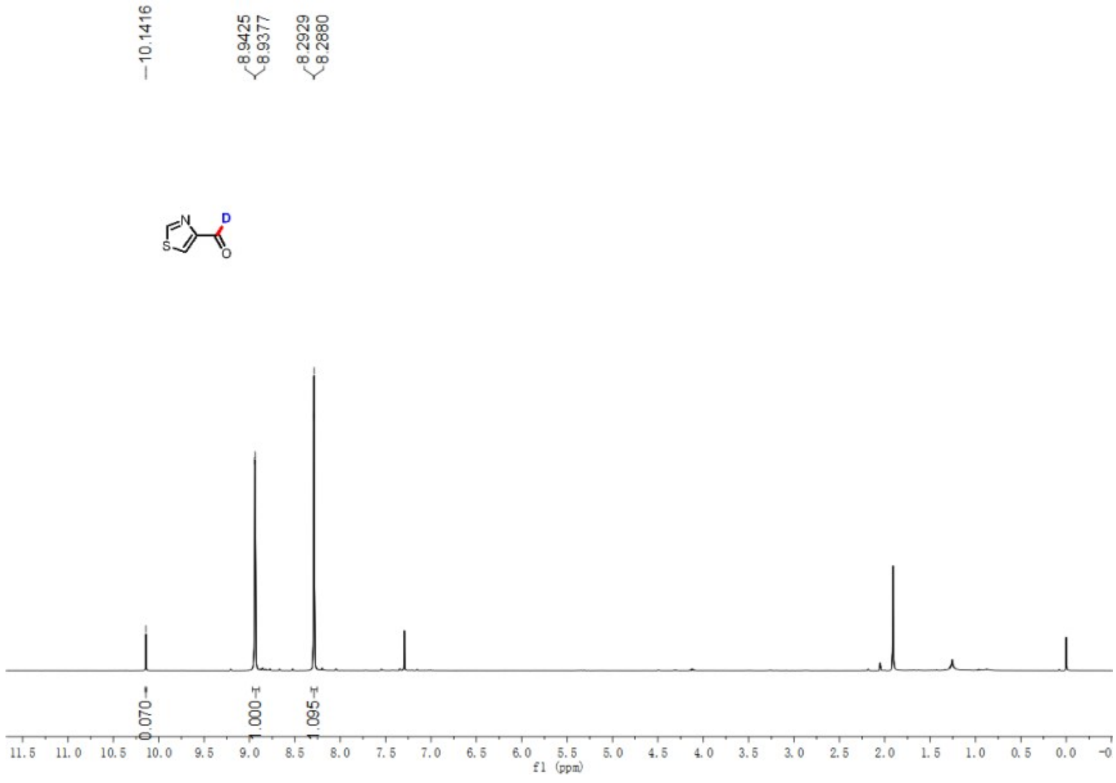

^{13}C NMR spectrum of compound **10ff**

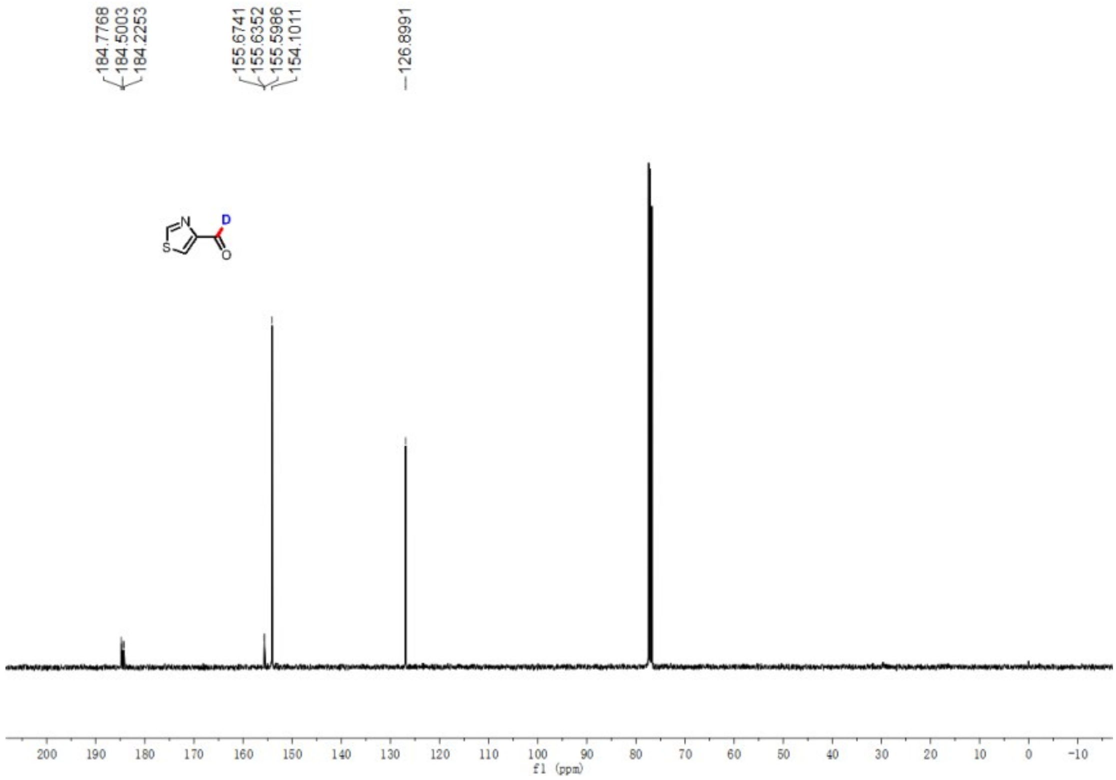

^{1}H NMR spectrum of compound **10gg**

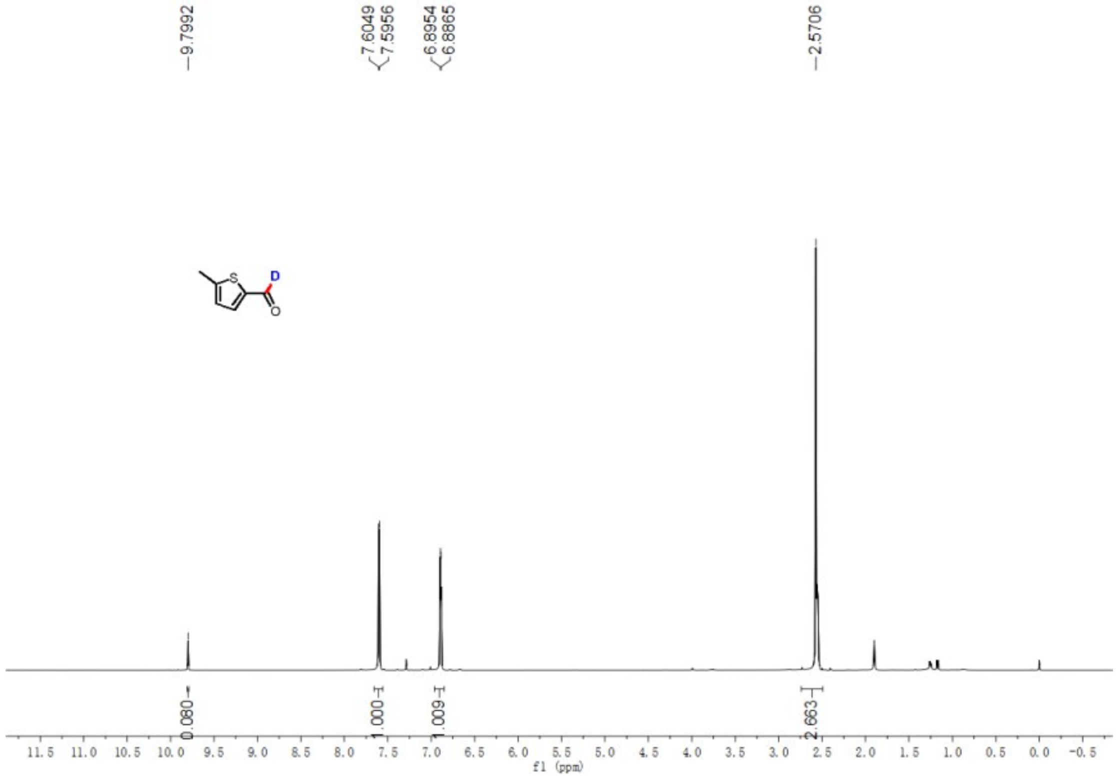

^{13}C NMR spectrum of compound **10gg**

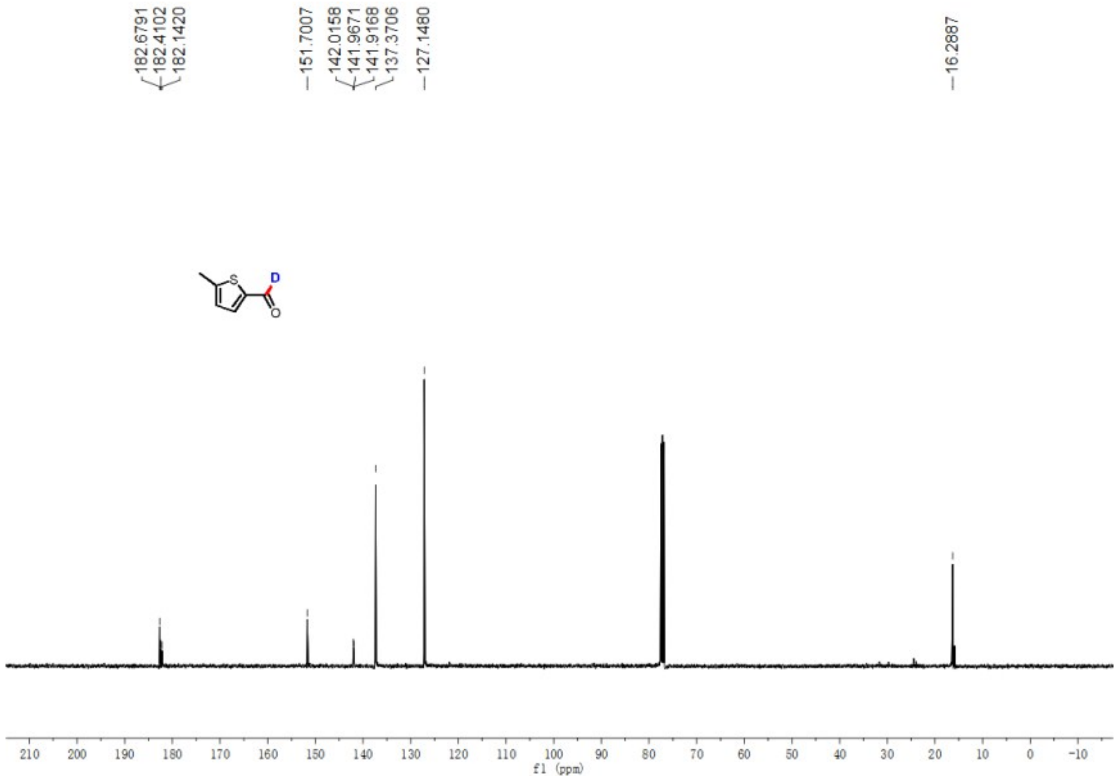

^{1}H NMR spectrum of compound **10hh**

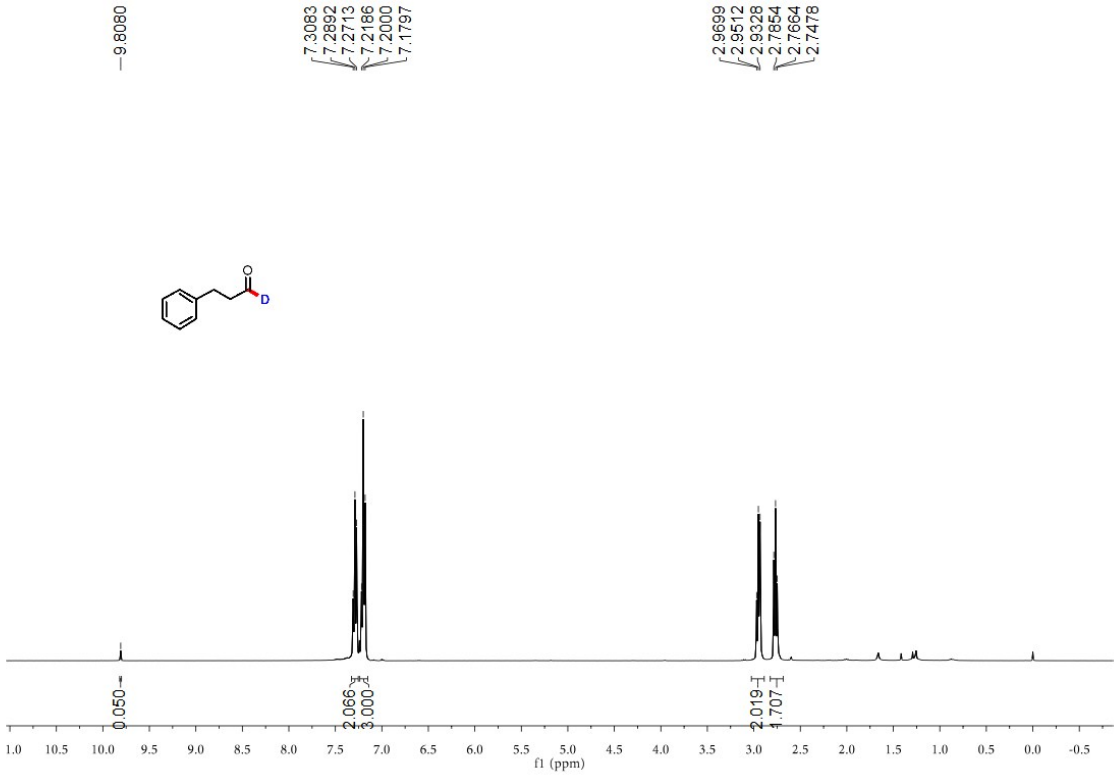

^{13}C NMR spectrum of compound **10hh**

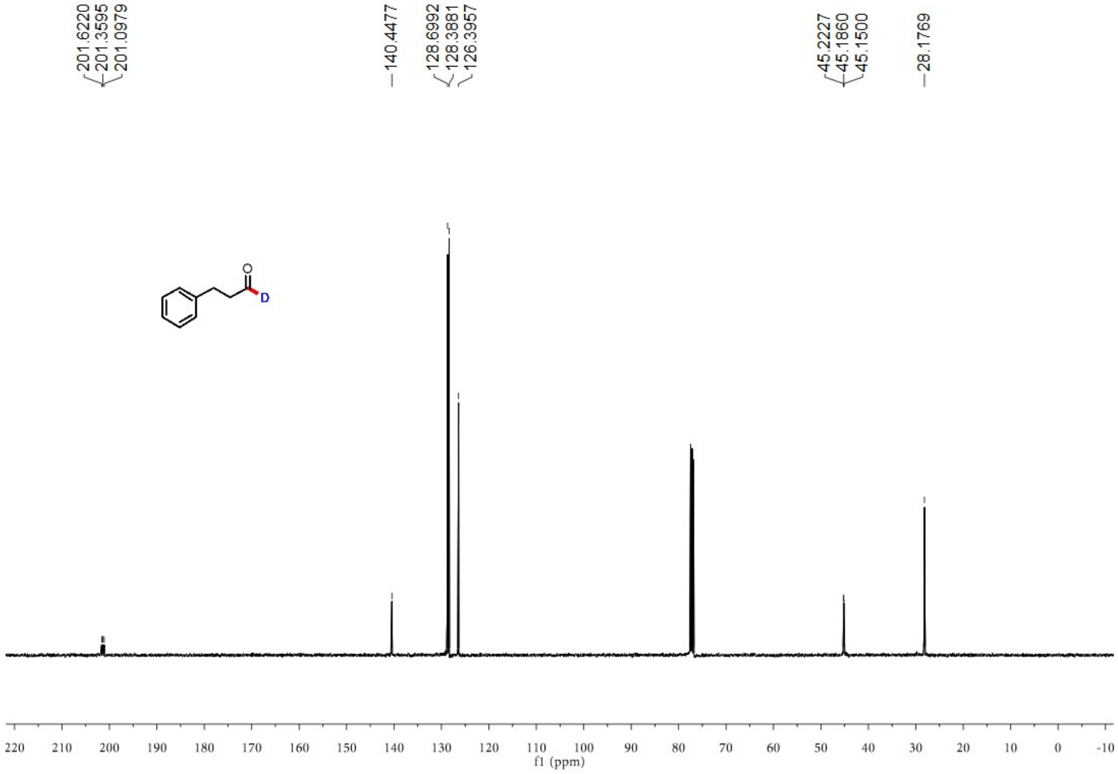

^{1}H NMR spectrum of compound **10ii**

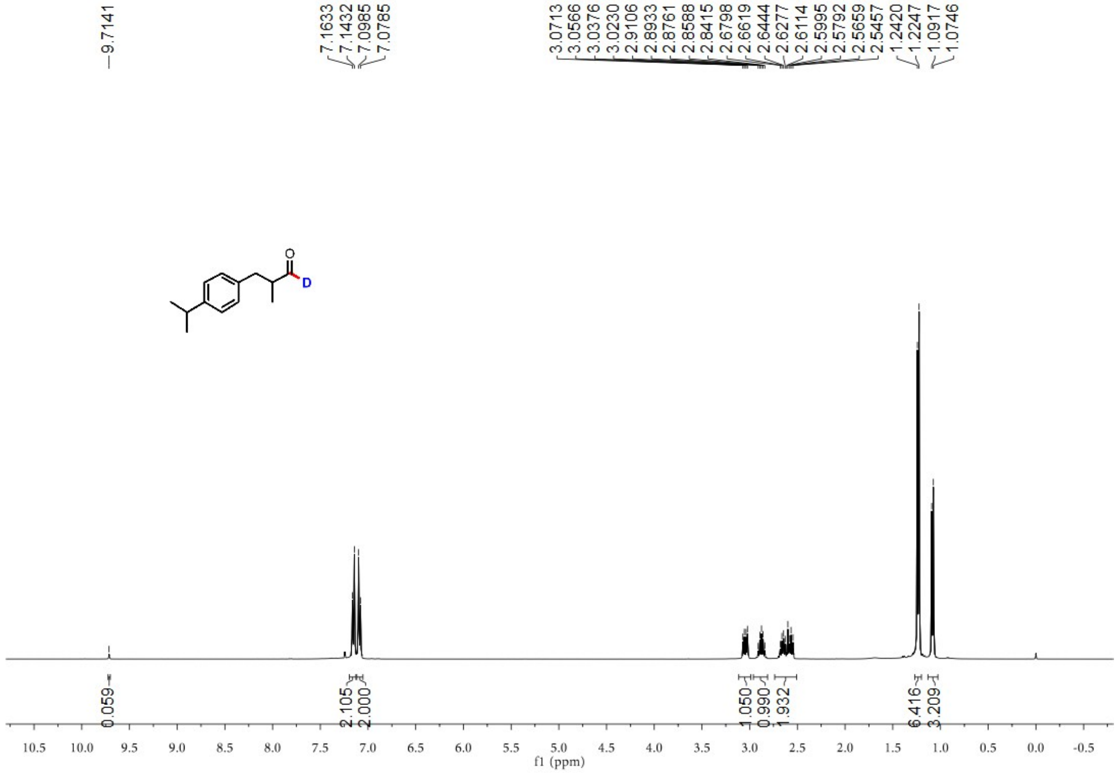

^{13}C NMR spectrum of compound **10ii**

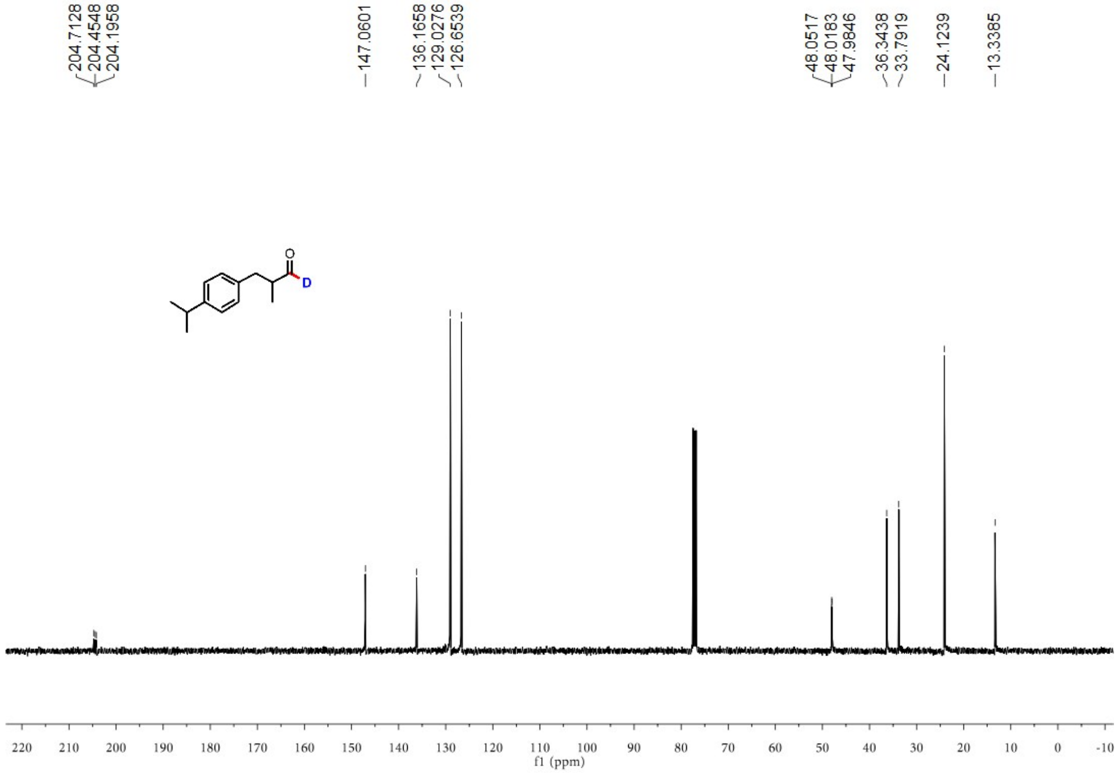

^{1}H NMR spectrum of compound **10jj**

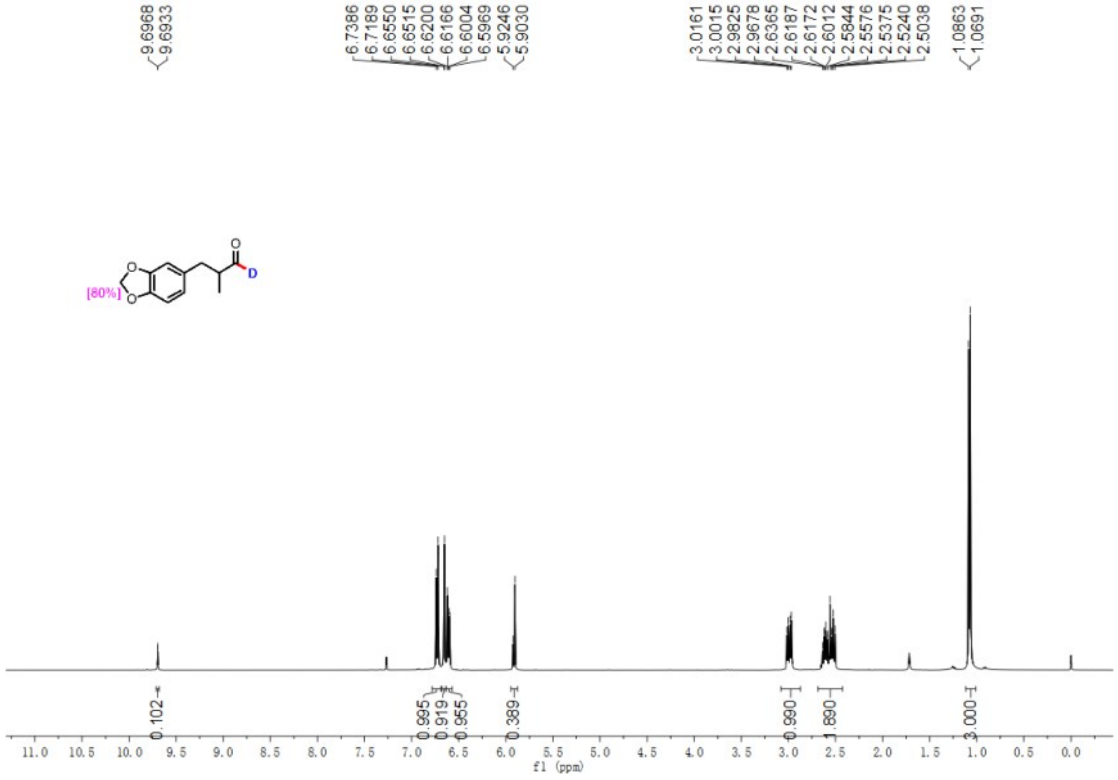

^{13}C NMR spectrum of compound **10jj**

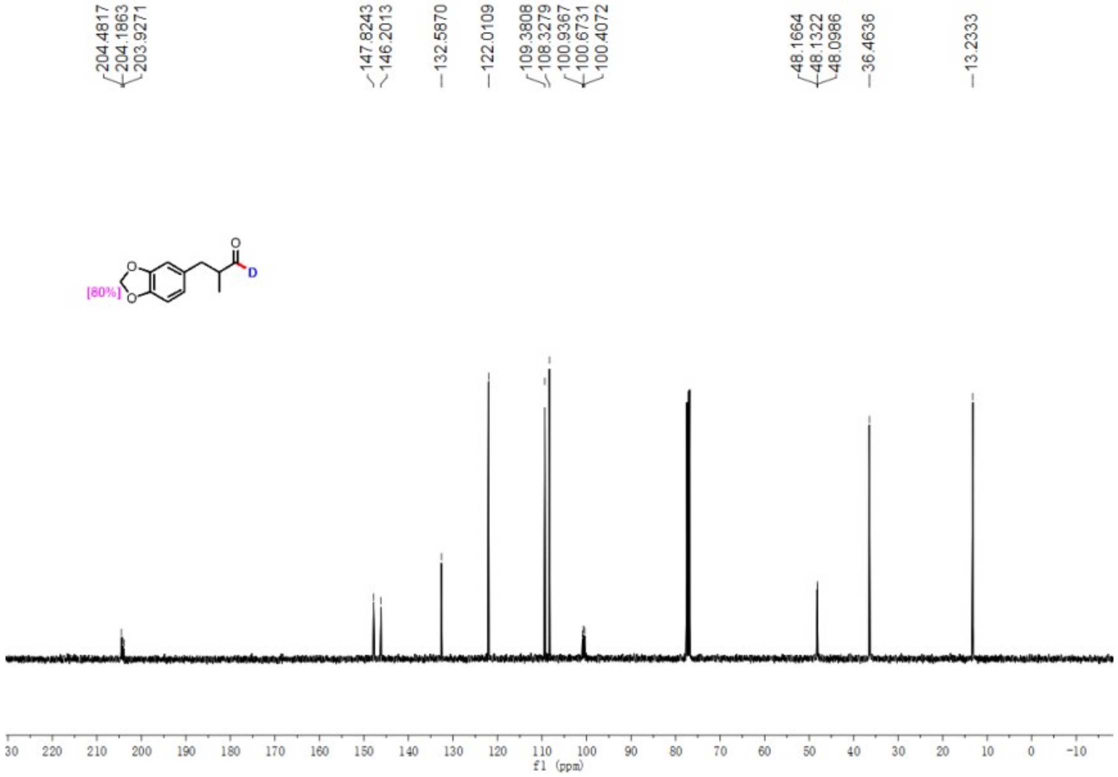

^{1}H NMR spectrum of compound **10kk**

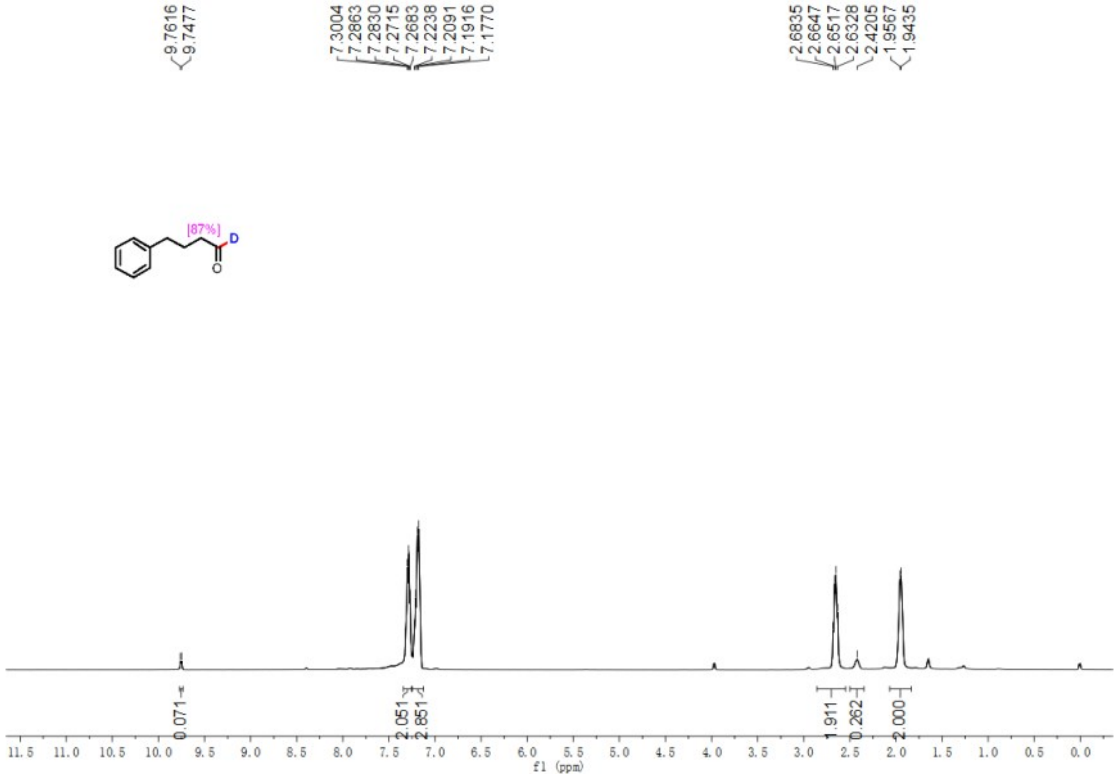

^{13}C NMR spectrum of compound **10kk**

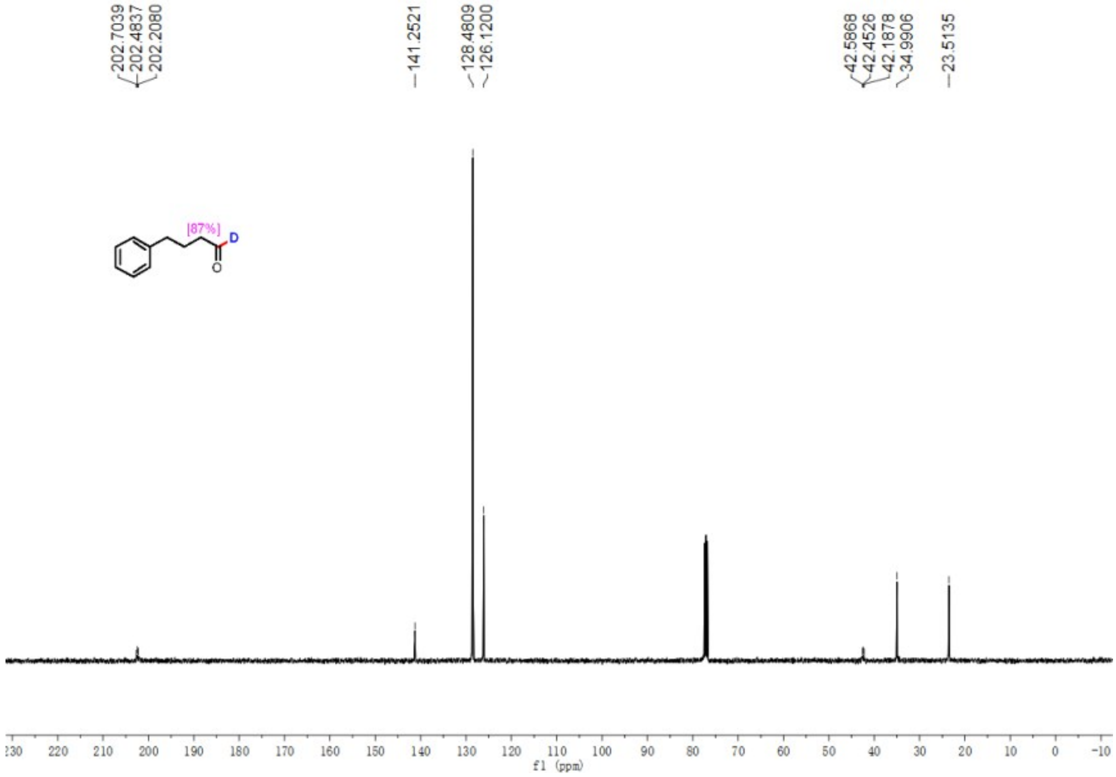

^{1}H NMR spectrum of compound **10ll**

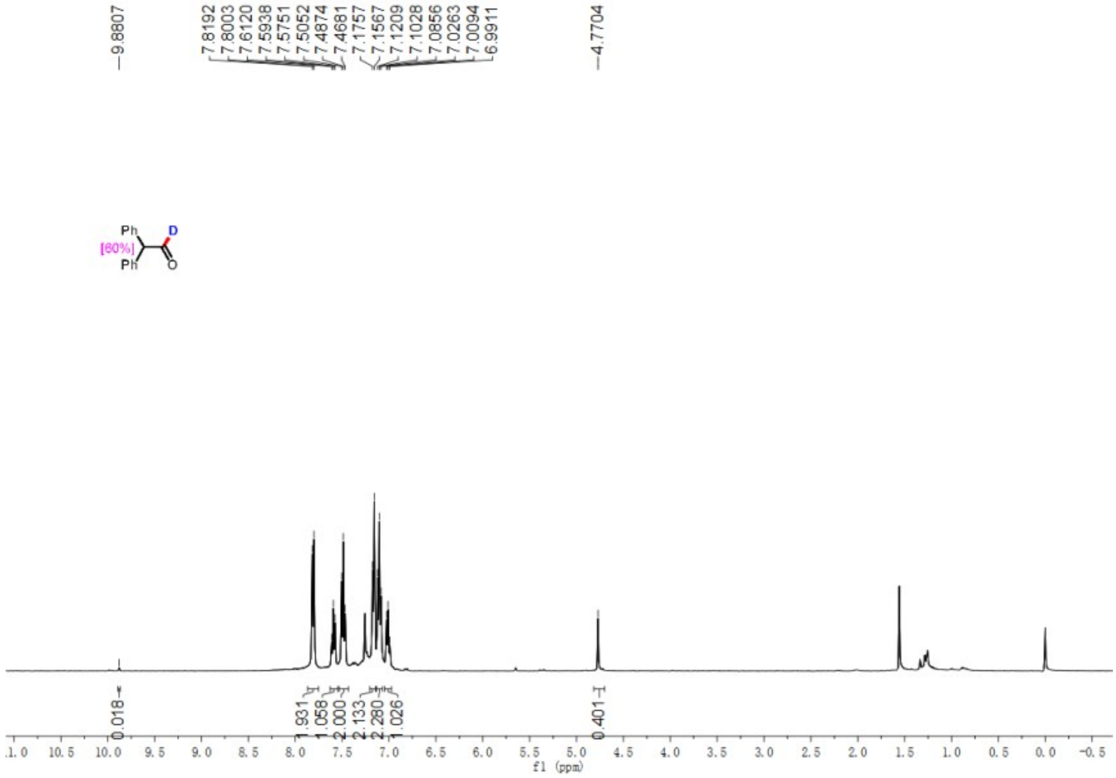

^{13}C NMR spectrum of compound **10ll**

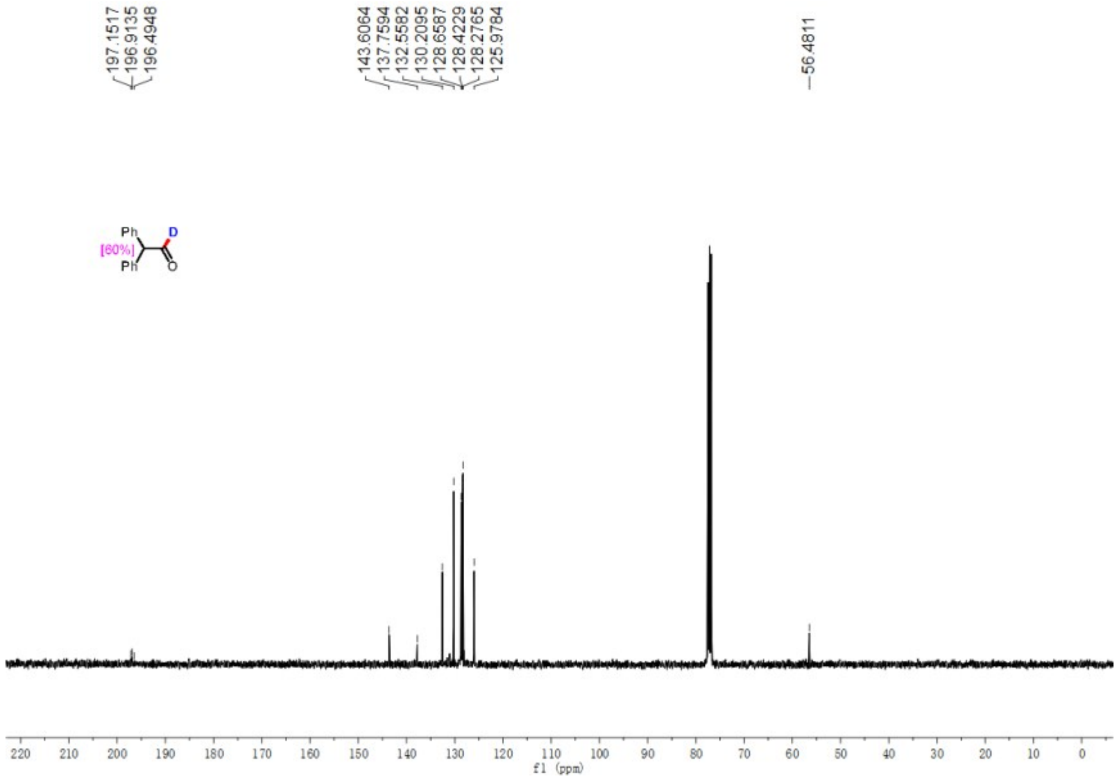

^{1}H NMR spectrum of compound **10mm**

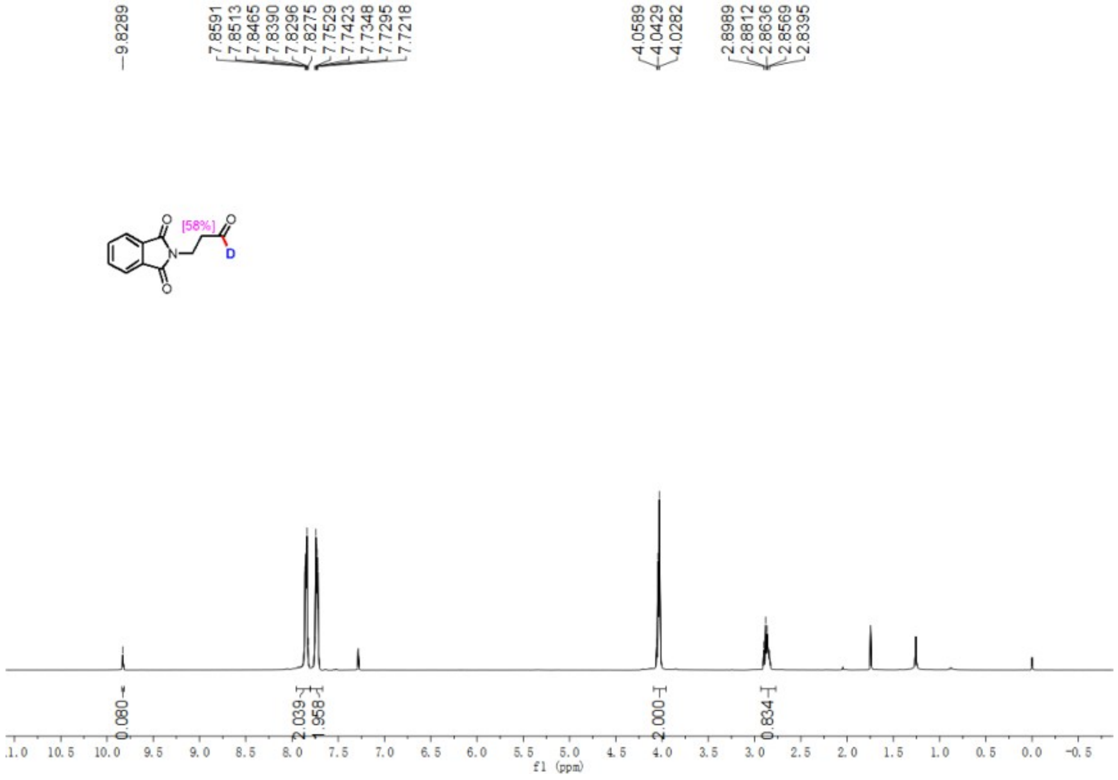

^{13}C NMR spectrum of compound **10mm**

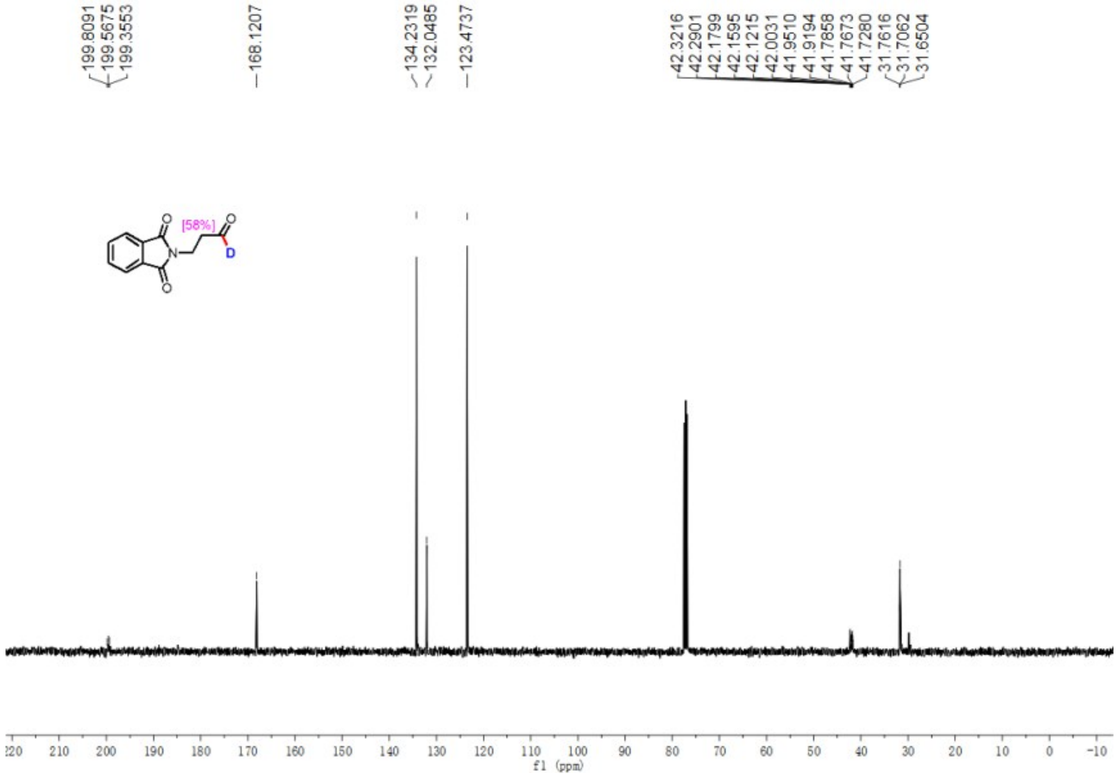

^{1}H NMR spectrum of compound **10nn**

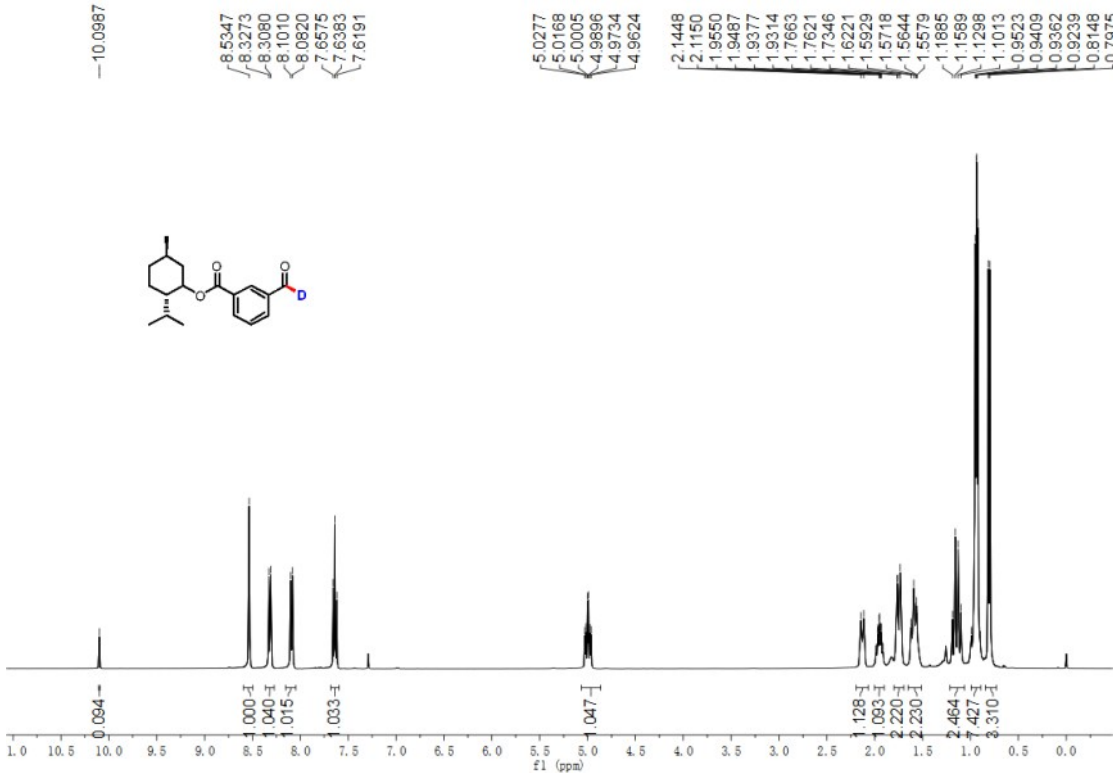

^{13}C NMR spectrum of compound **10nn**

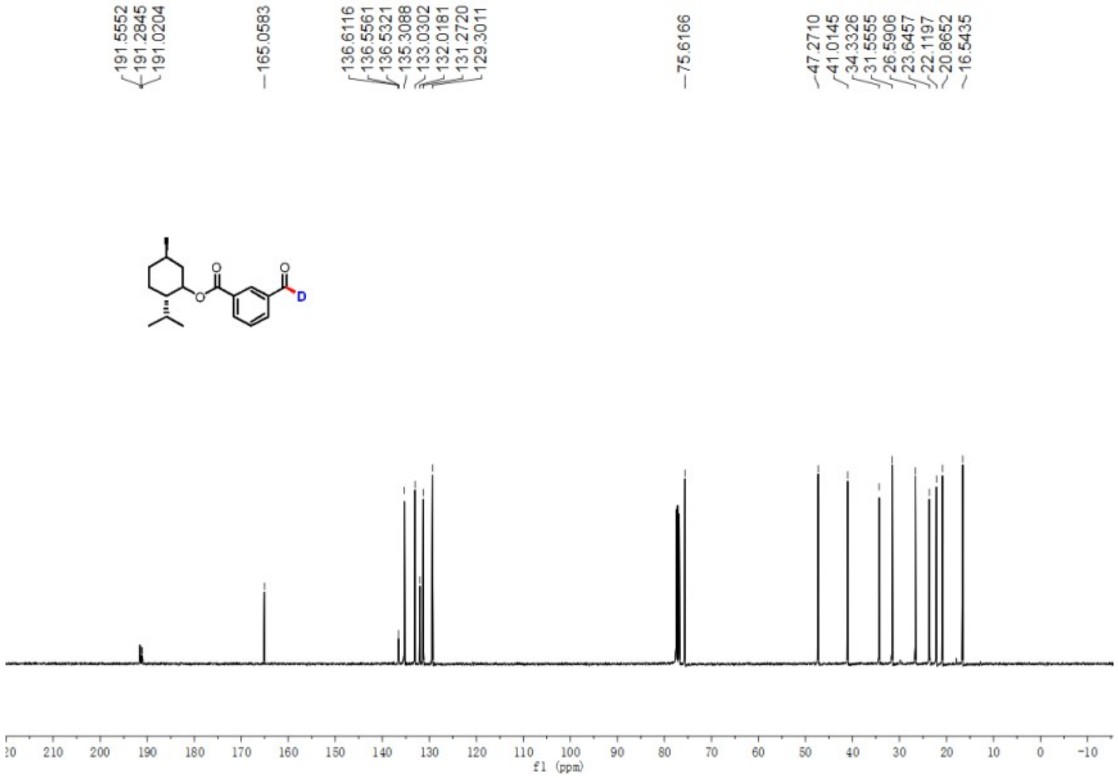

^{1}H NMR spectrum of compound **1oo**

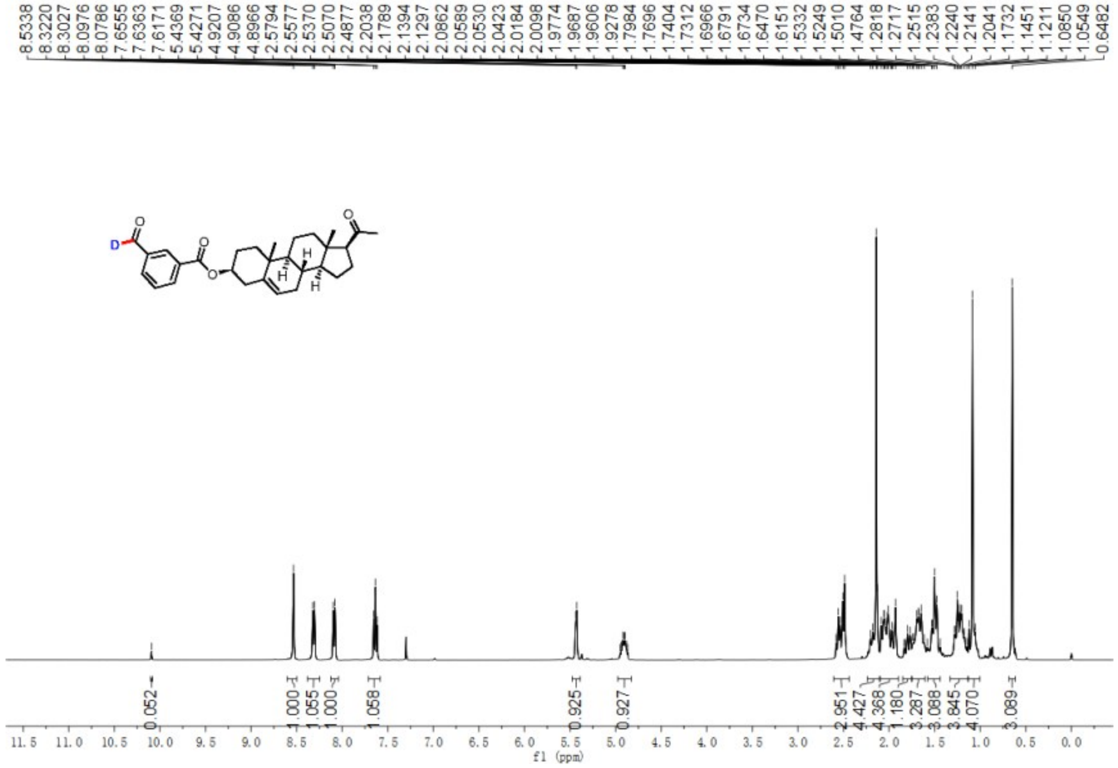

^{13}C NMR spectrum of compound **10oo**

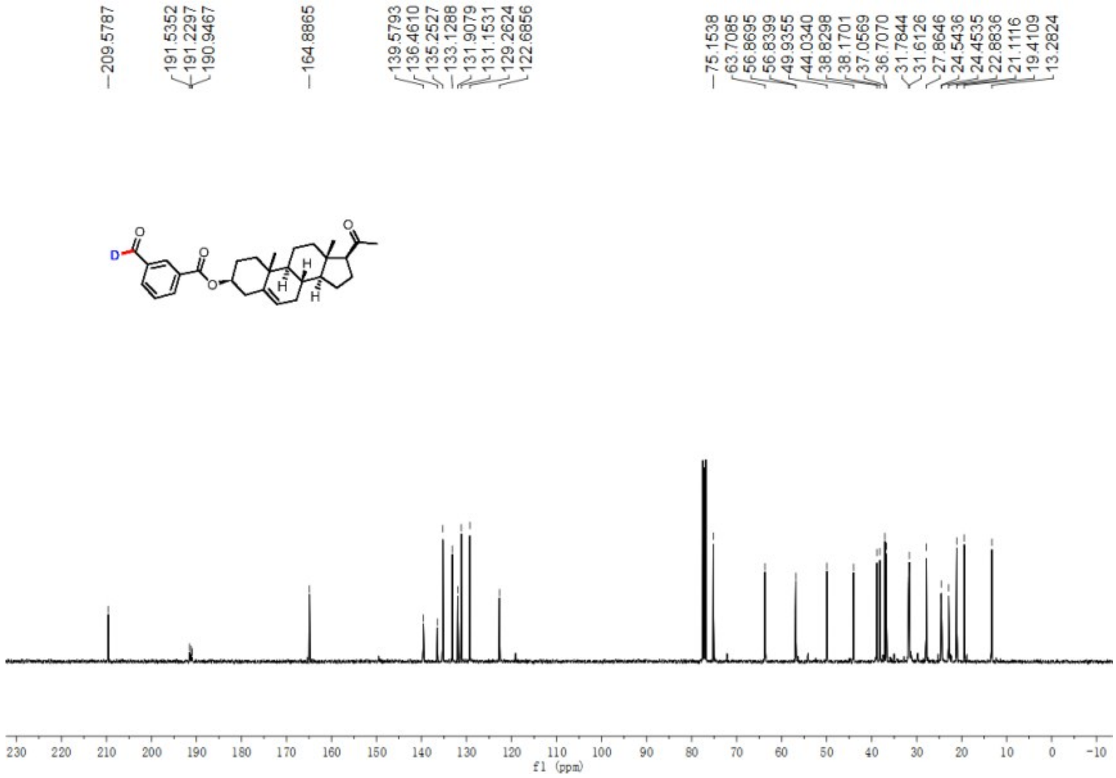

^{1}H NMR spectrum of compound **10pp**

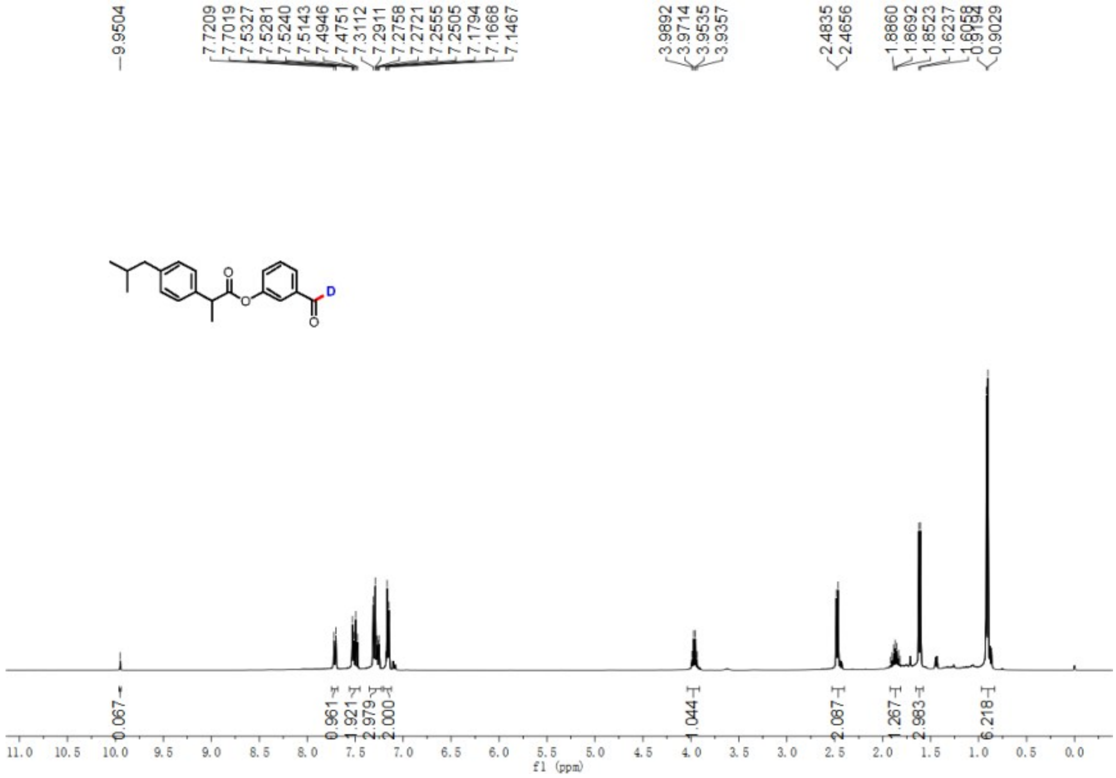

^{13}C NMR spectrum of compound **10pp**

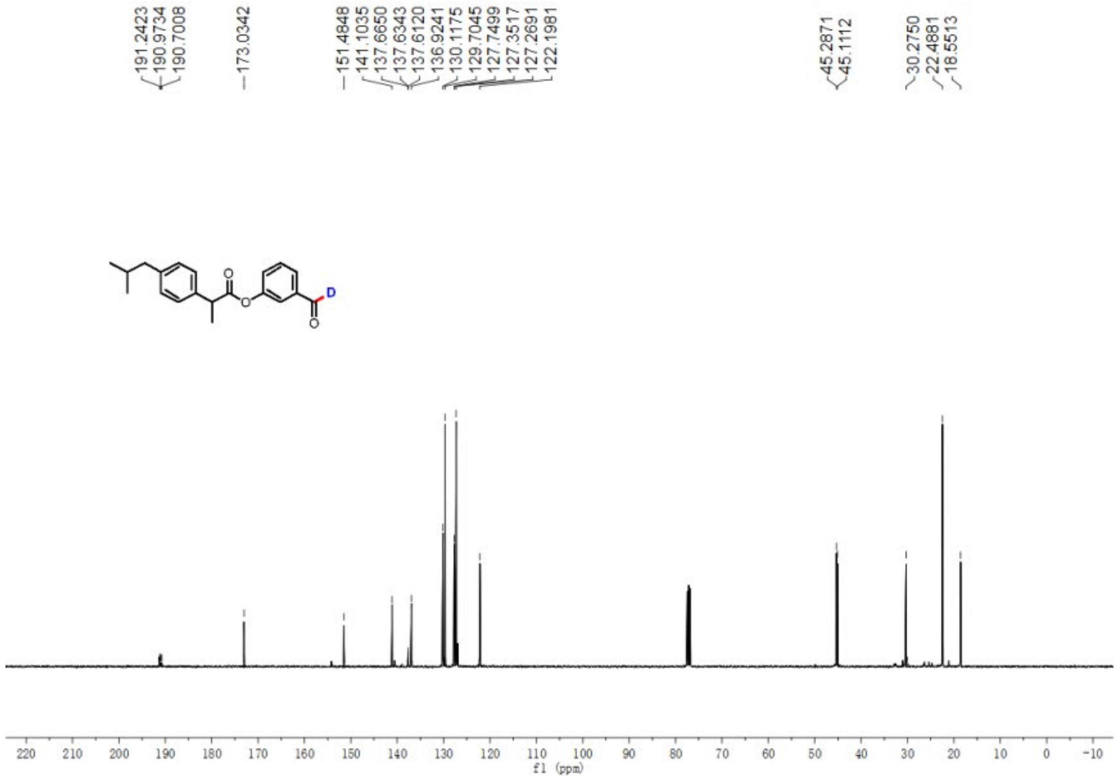

^{1}H NMR spectrum of compound **10qq**

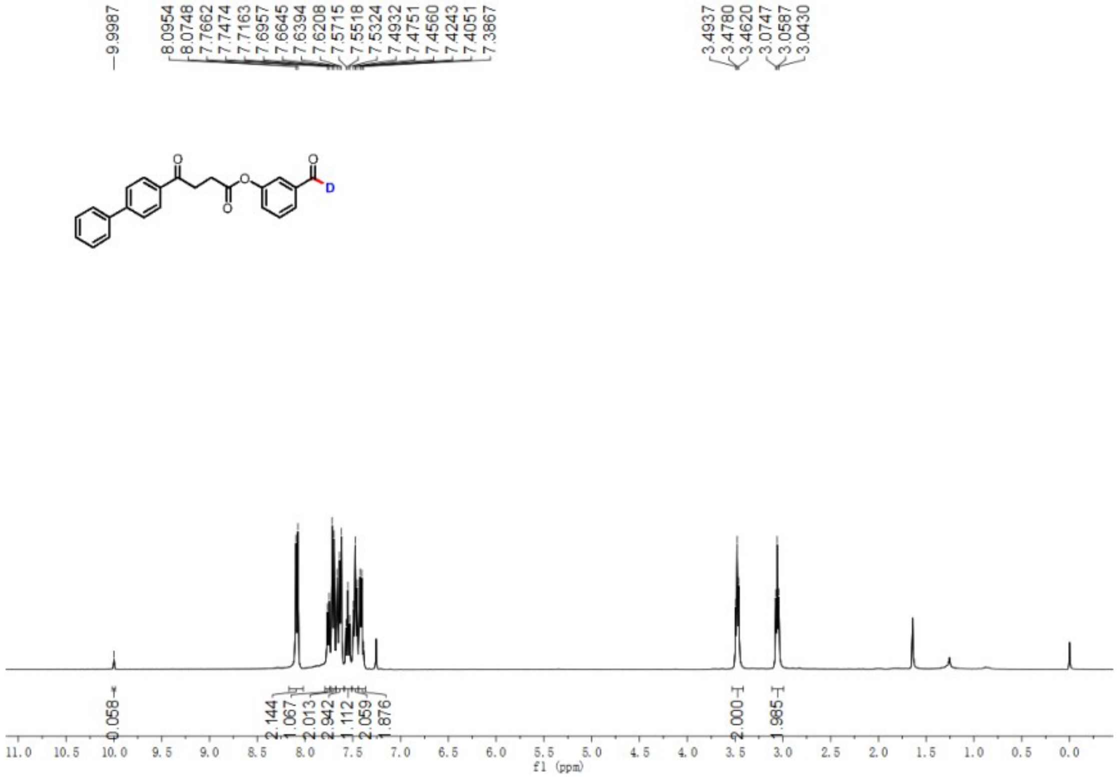

^{13}C NMR spectrum of compound **10qq**

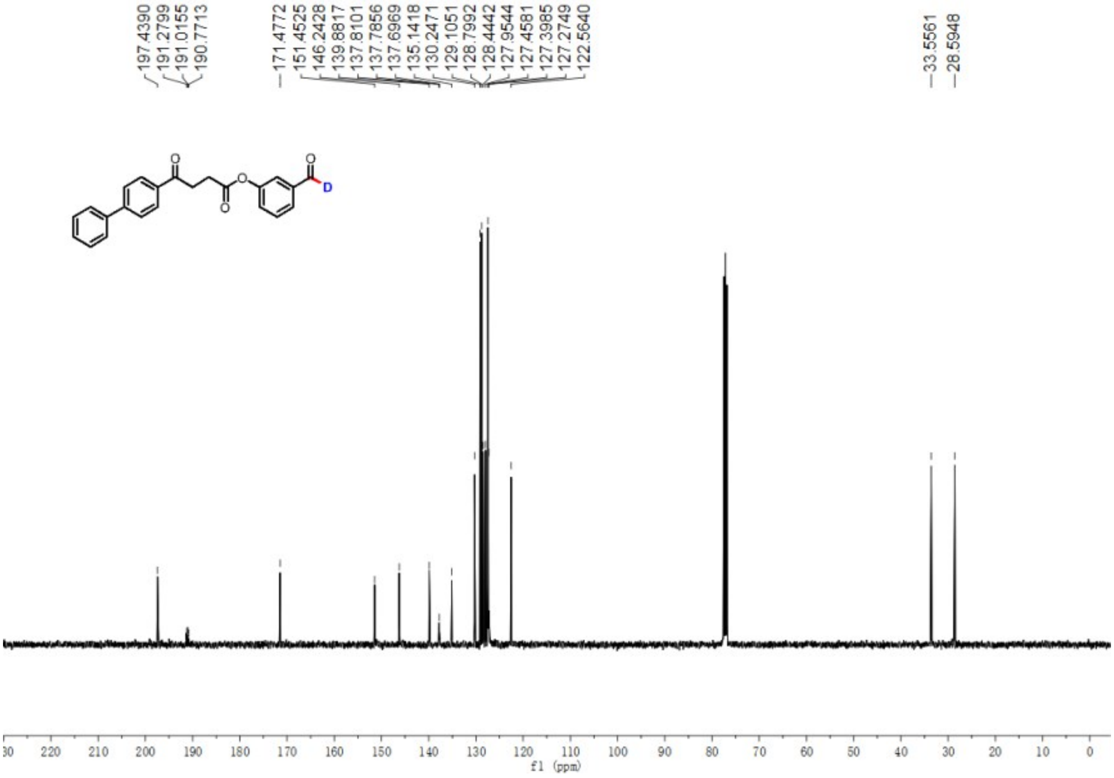

^{1}H NMR spectrum of compound **10rr**

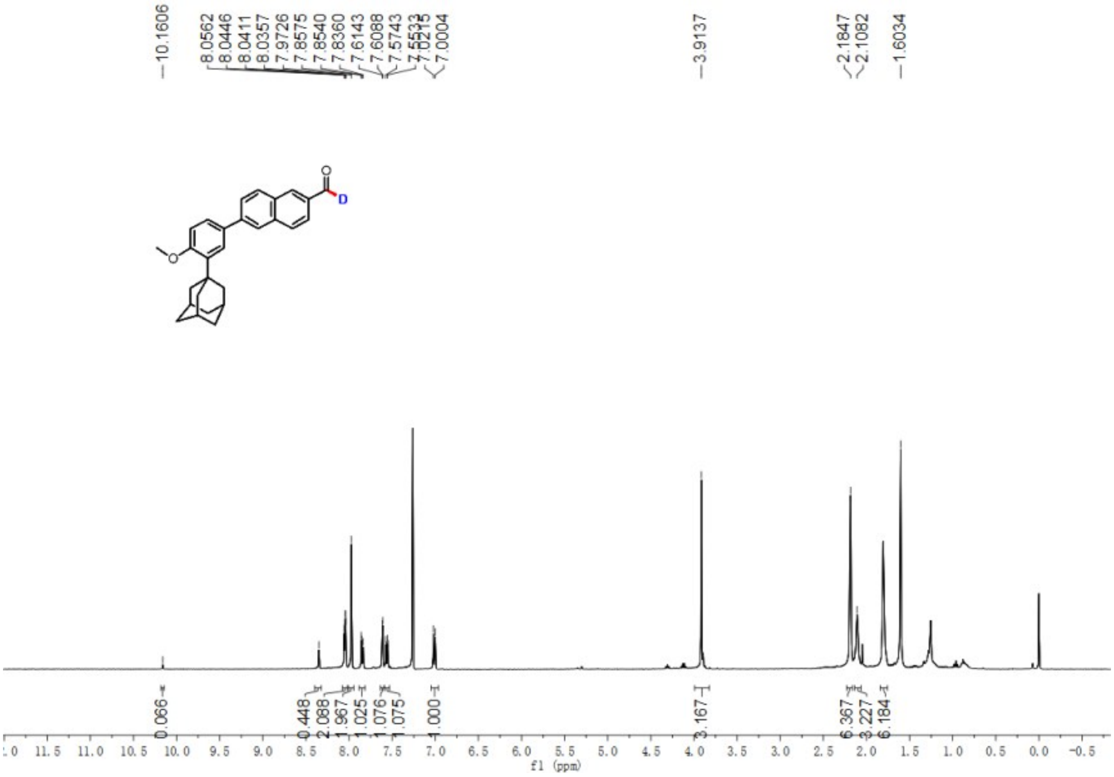

^{13}C NMR spectrum of compound **10rr**

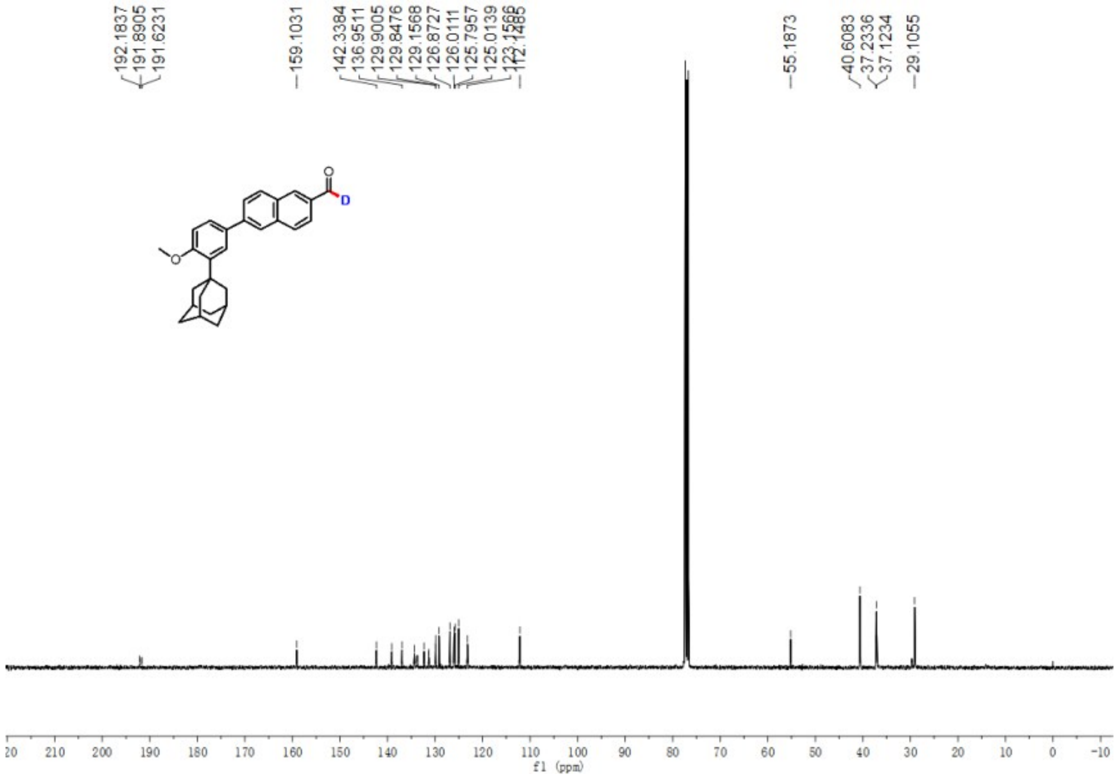

^{1}H NMR spectrum of compound **11**

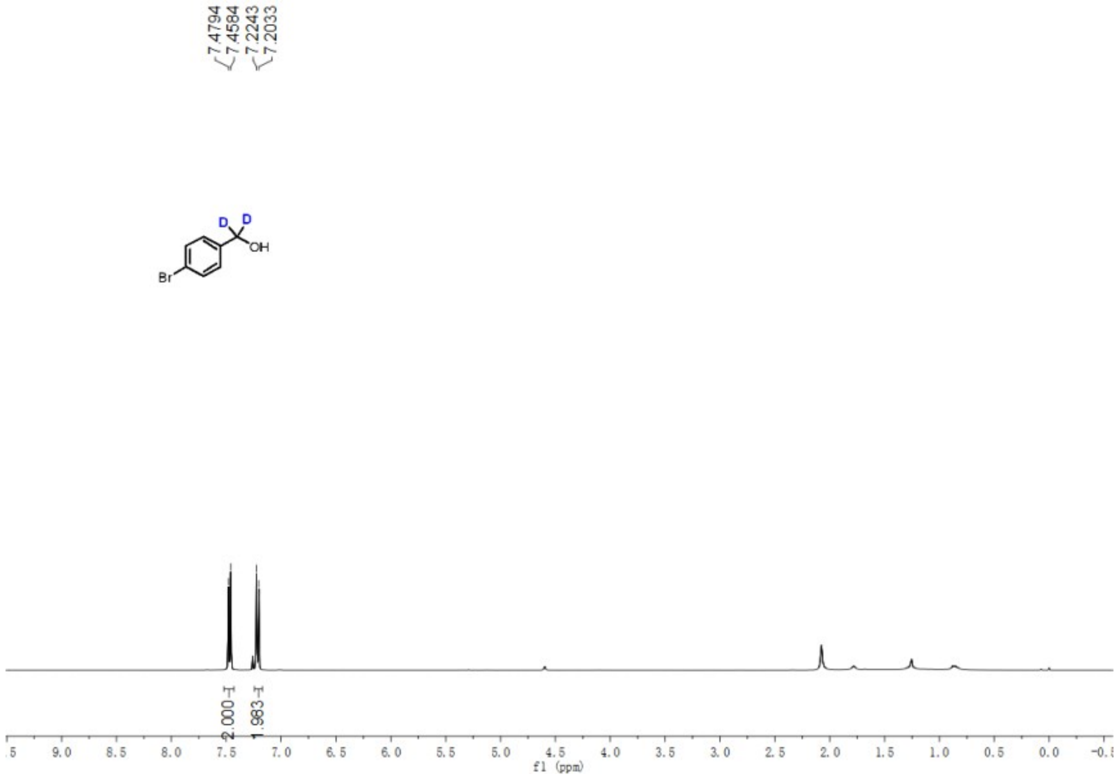

^{13}C NMR spectrum of compound **11**

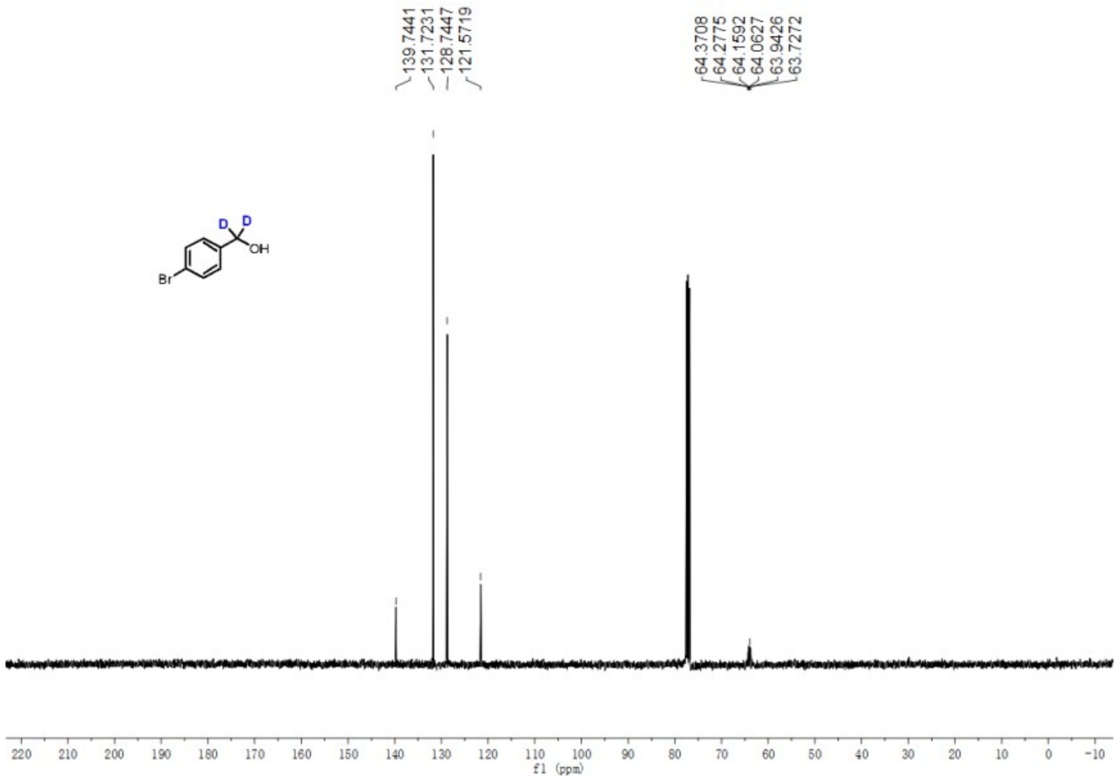

^{1}H NMR spectrum of compound **12**

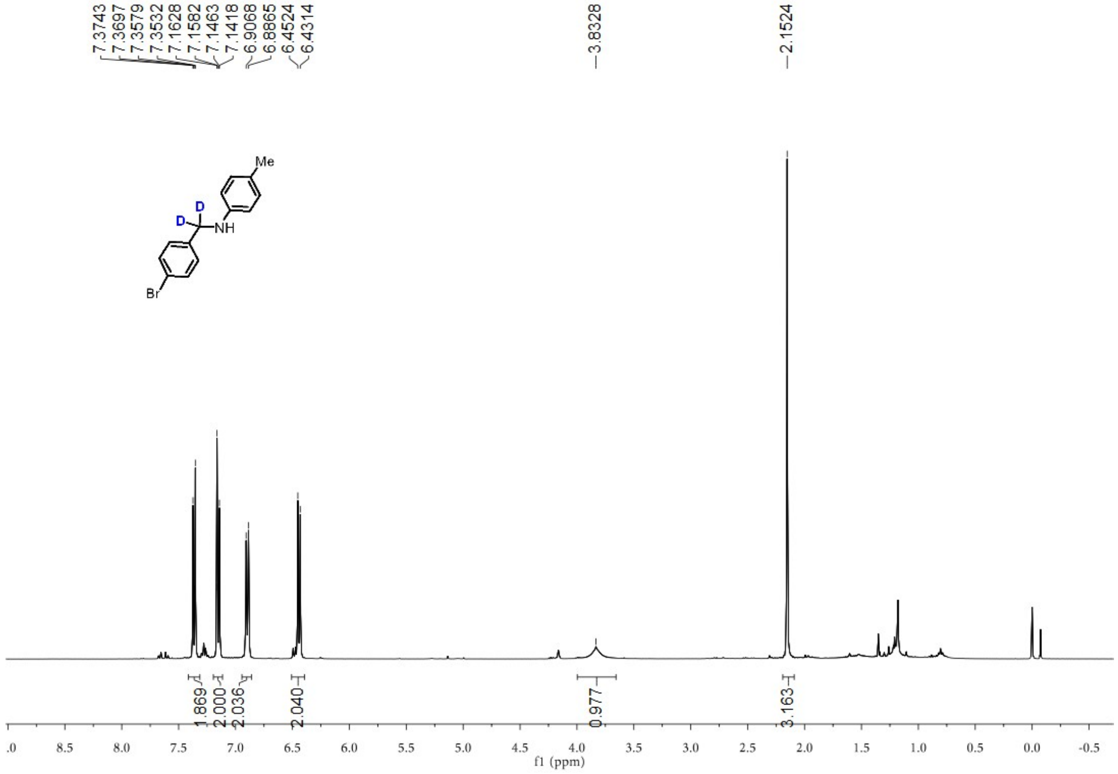

^{13}C NMR spectrum of compound **12**

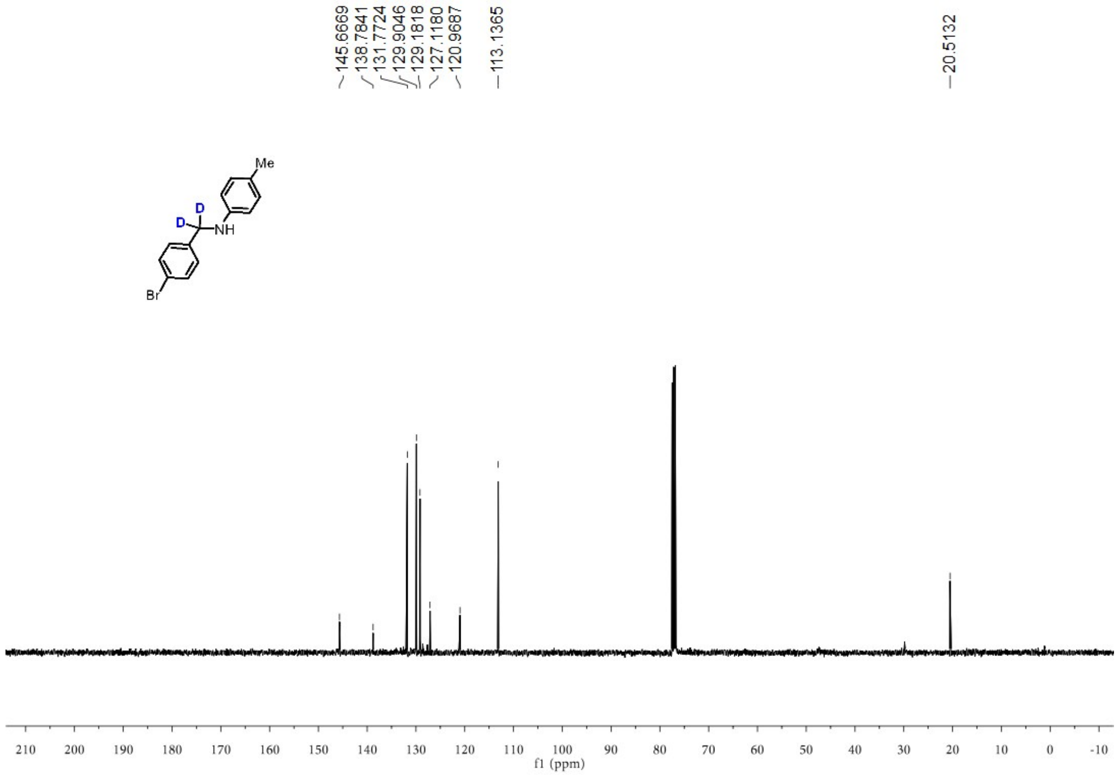

^{1}H NMR spectrum of compound **13**

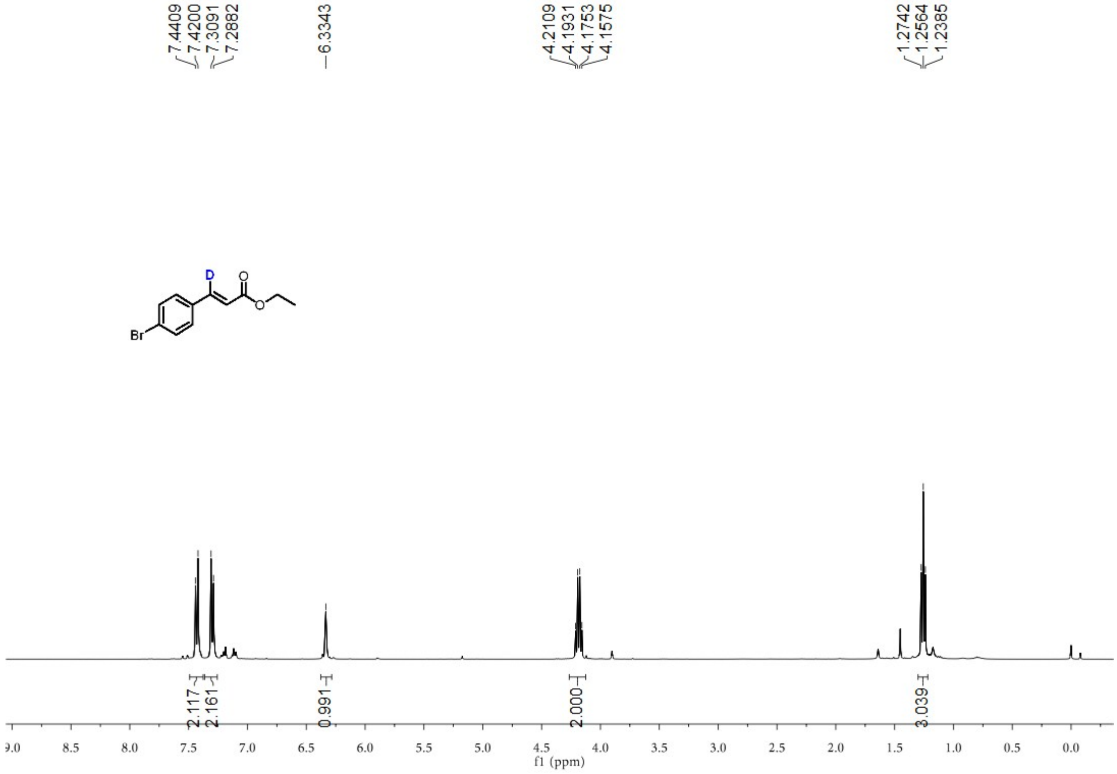

^{13}C NMR spectrum of compound **13**

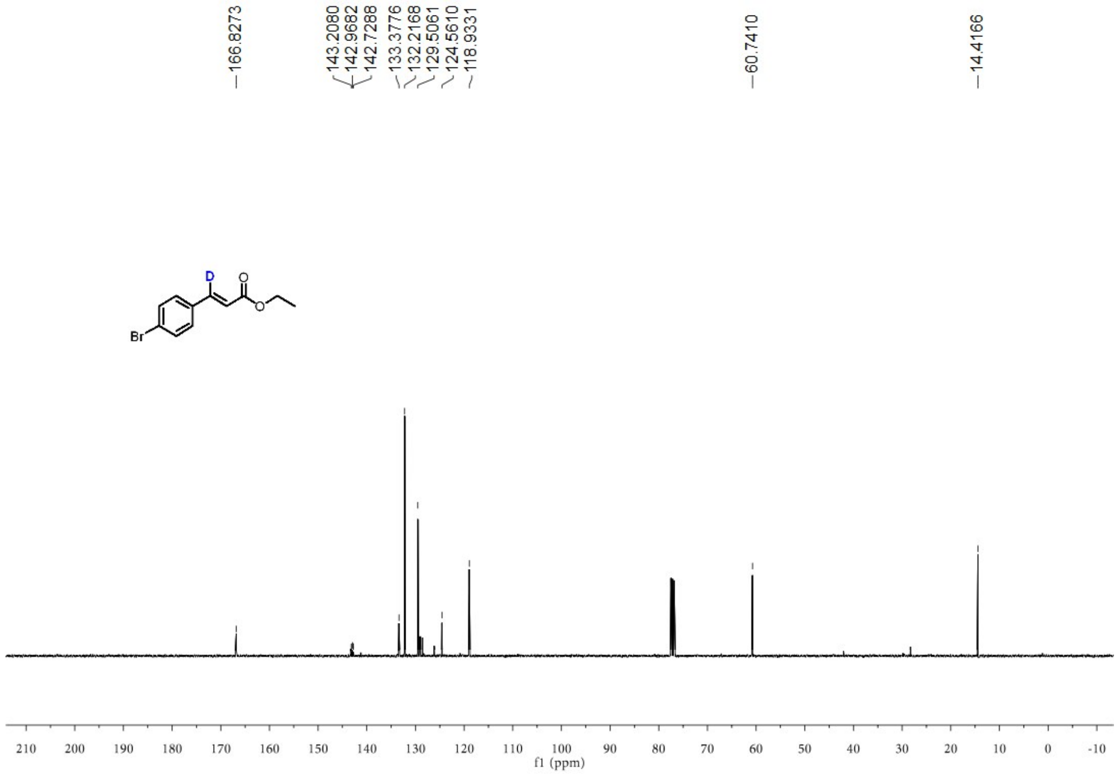

^{1}H NMR spectrum of compound **14**

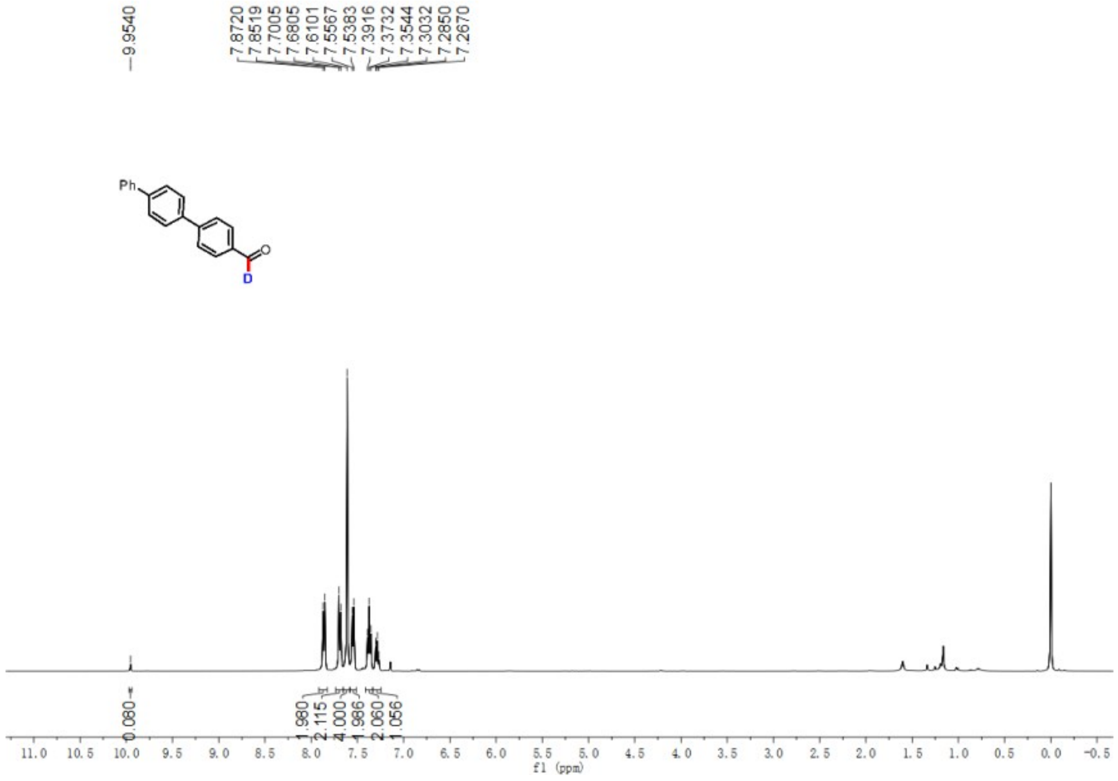

^{13}C NMR spectrum of compound **14**

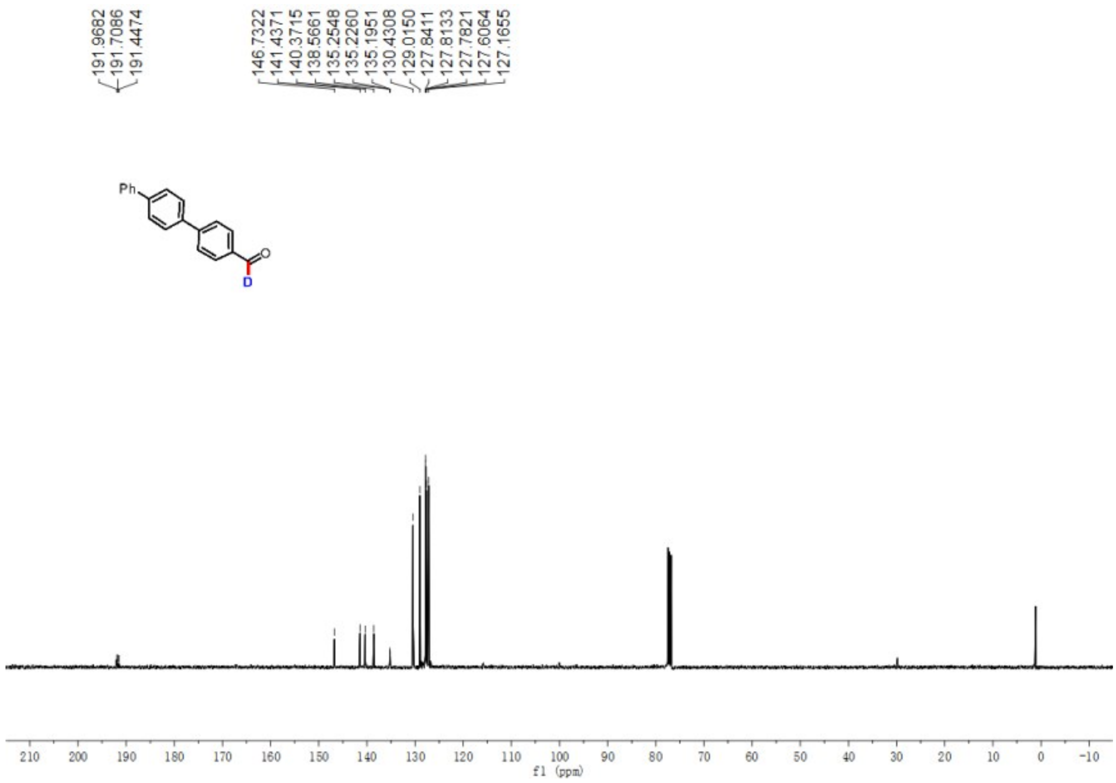

^{1}H NMR spectrum of compound **15**

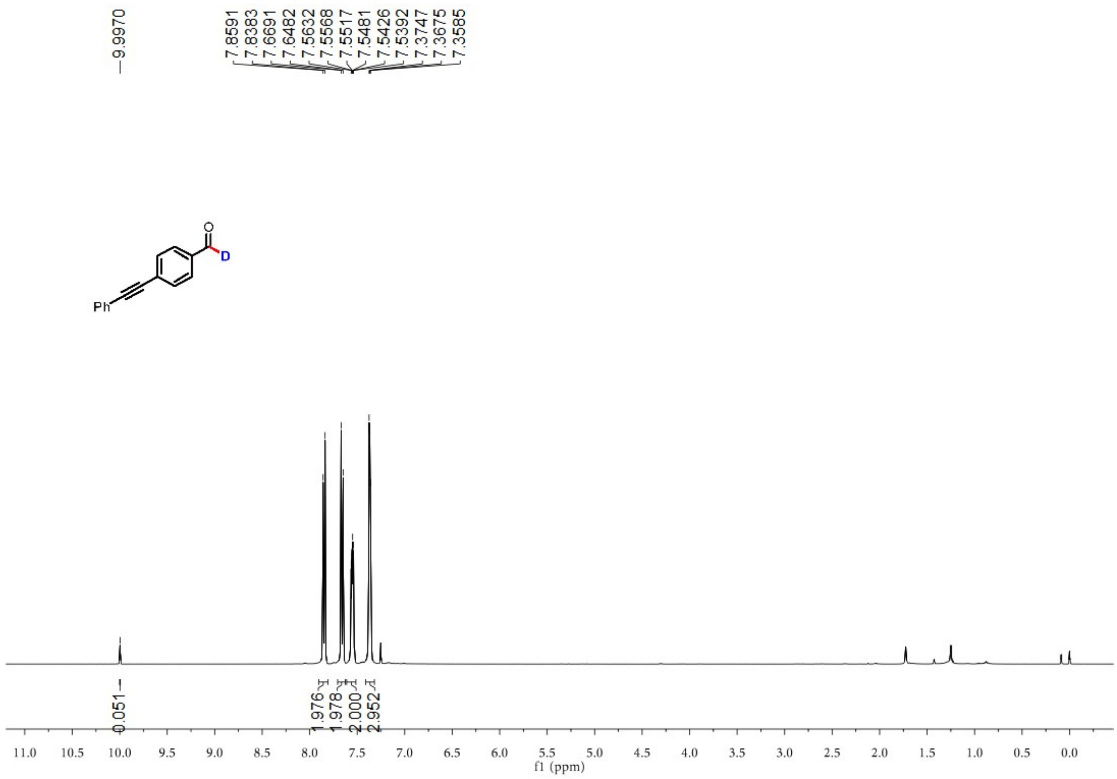

^{13}C NMR spectrum of compound **15**

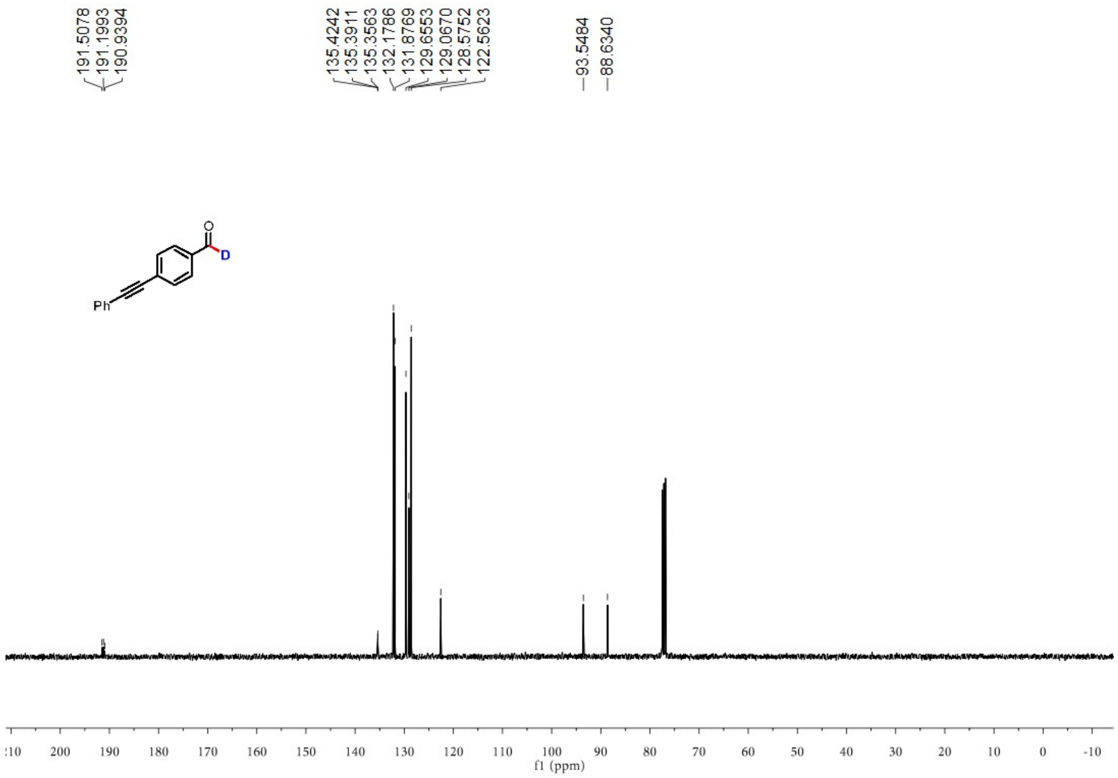

^{1}H NMR spectrum of compound **10tt**

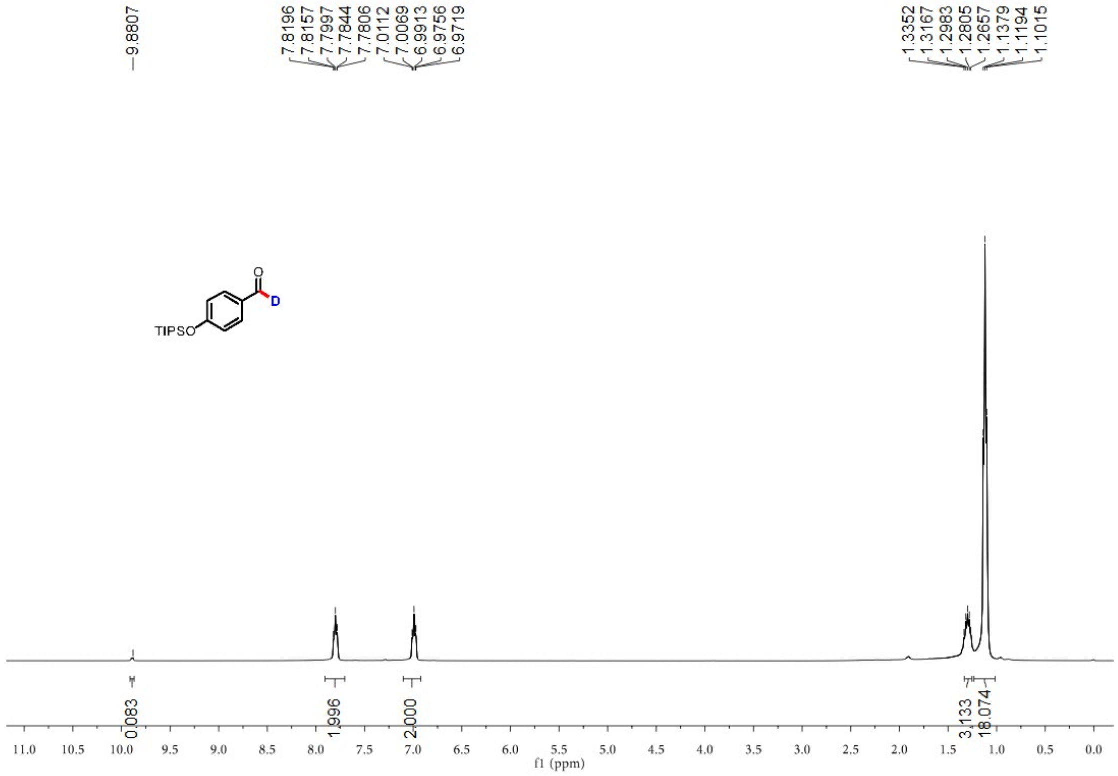

^{13}C NMR spectrum of compound **10tt**

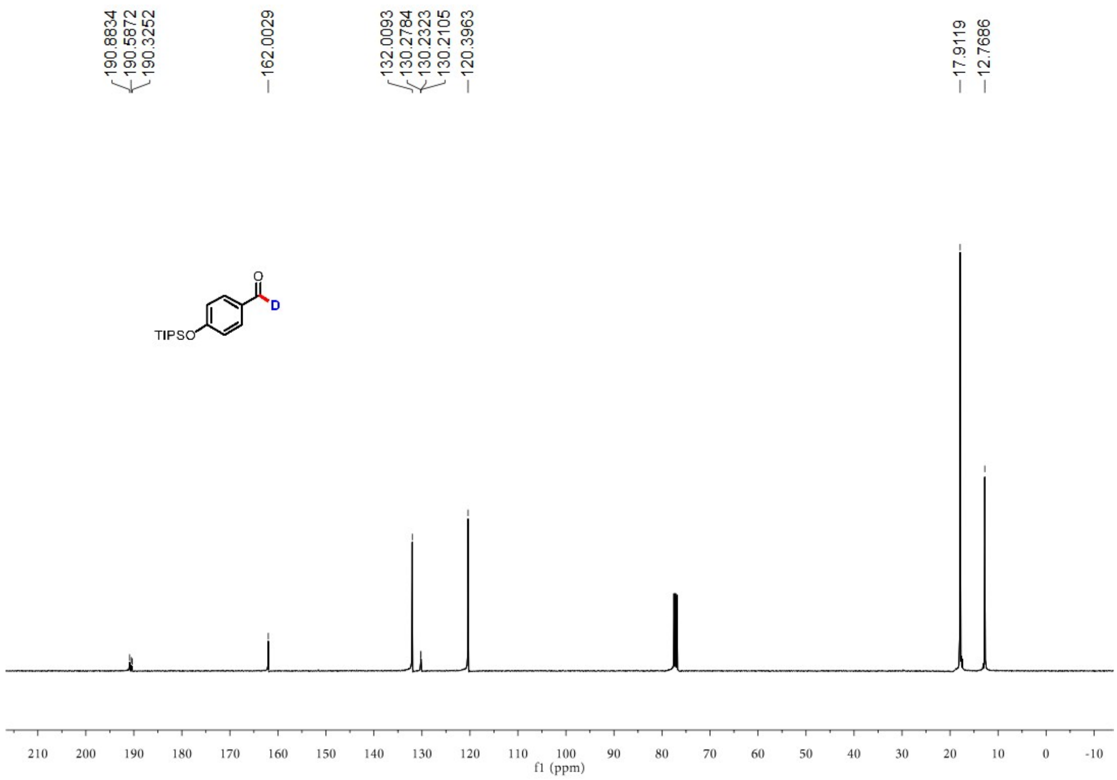

^{1}H NMR spectrum of compound **16**

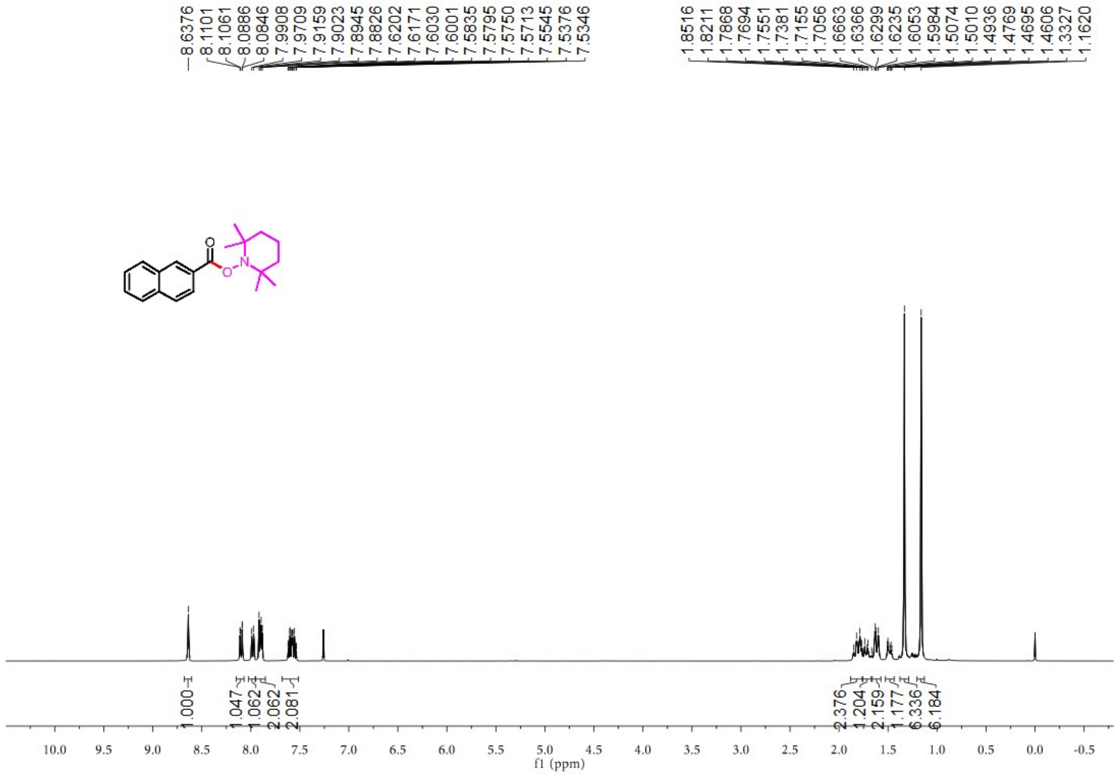

^{13}C NMR spectrum of compound **16**

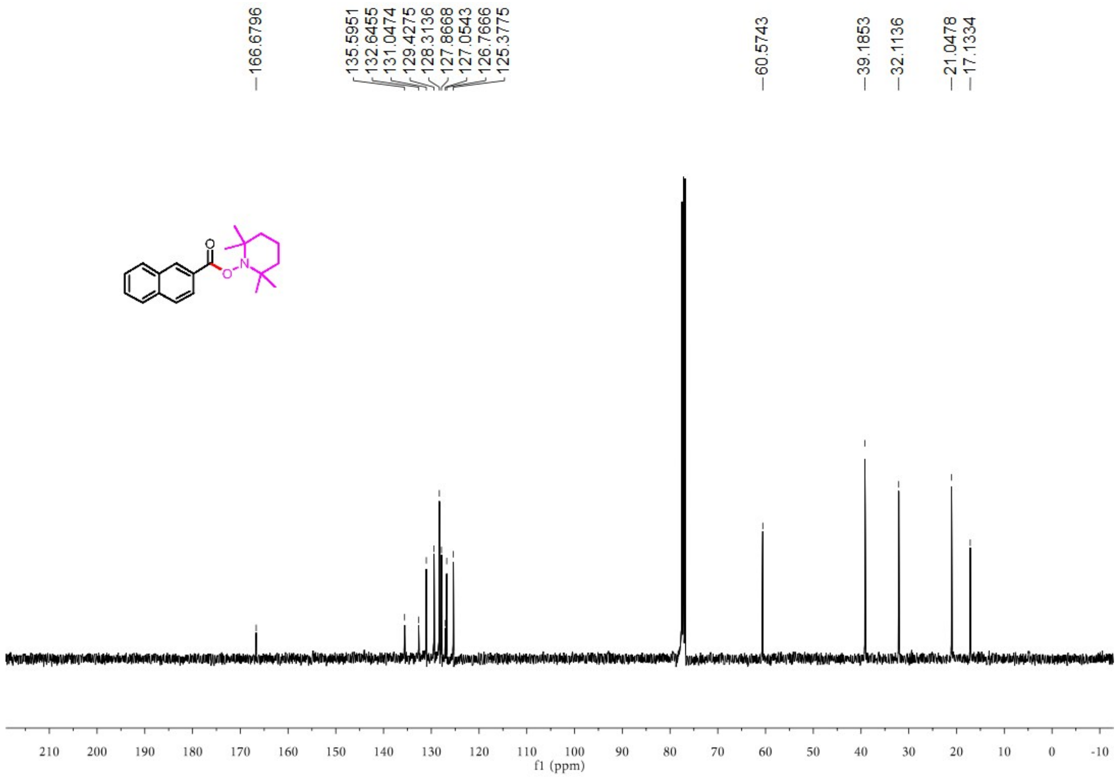

^{1}H NMR spectrum of compound **18**

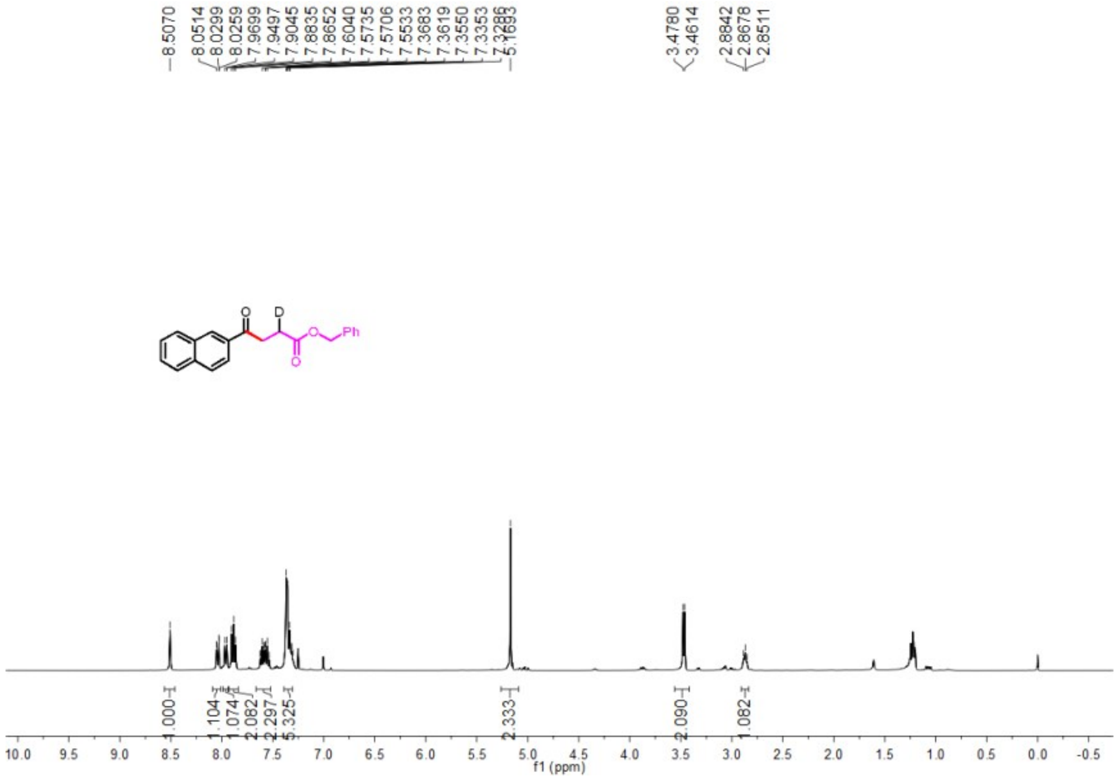

^{13}C NMR spectrum of compound **18**

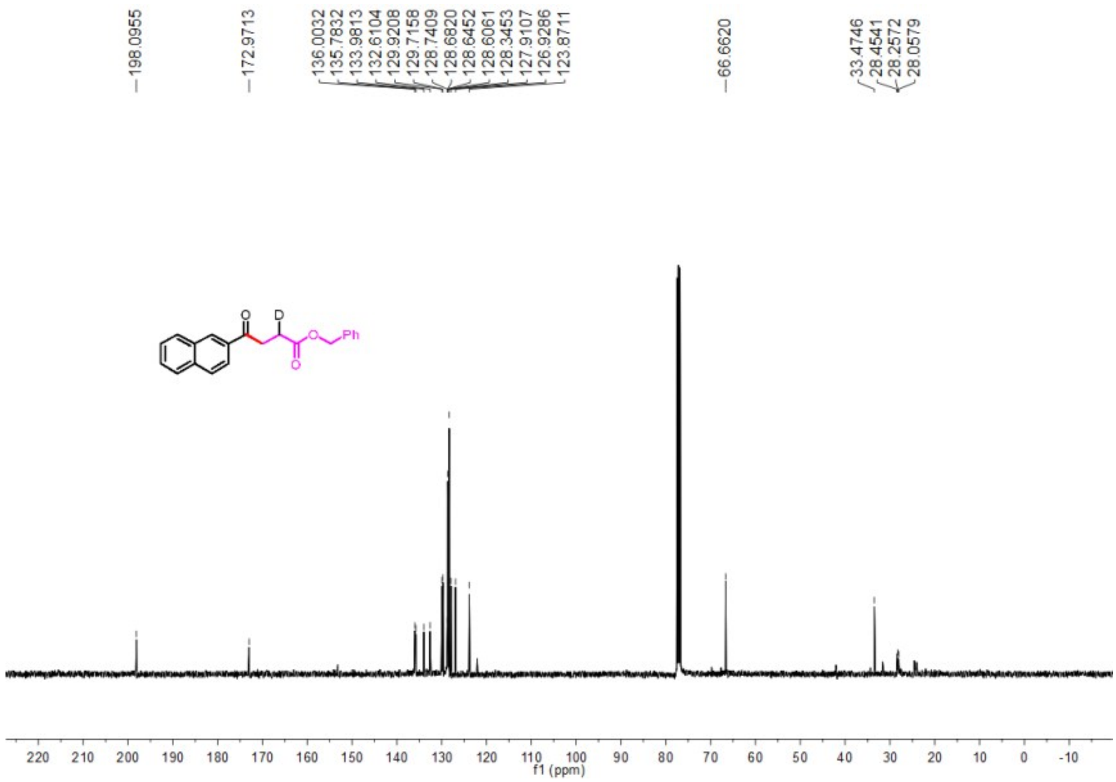

^{1}H NMR spectrum of compound **19**

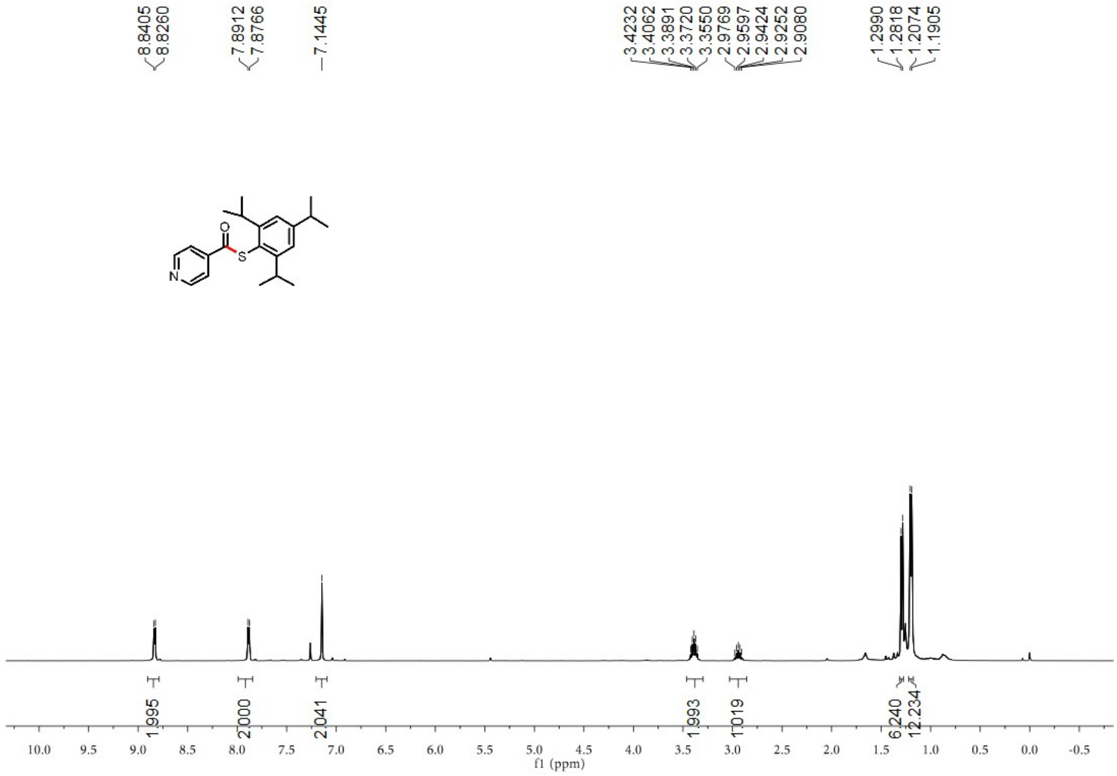

^{13}C NMR spectrum of compound **19**

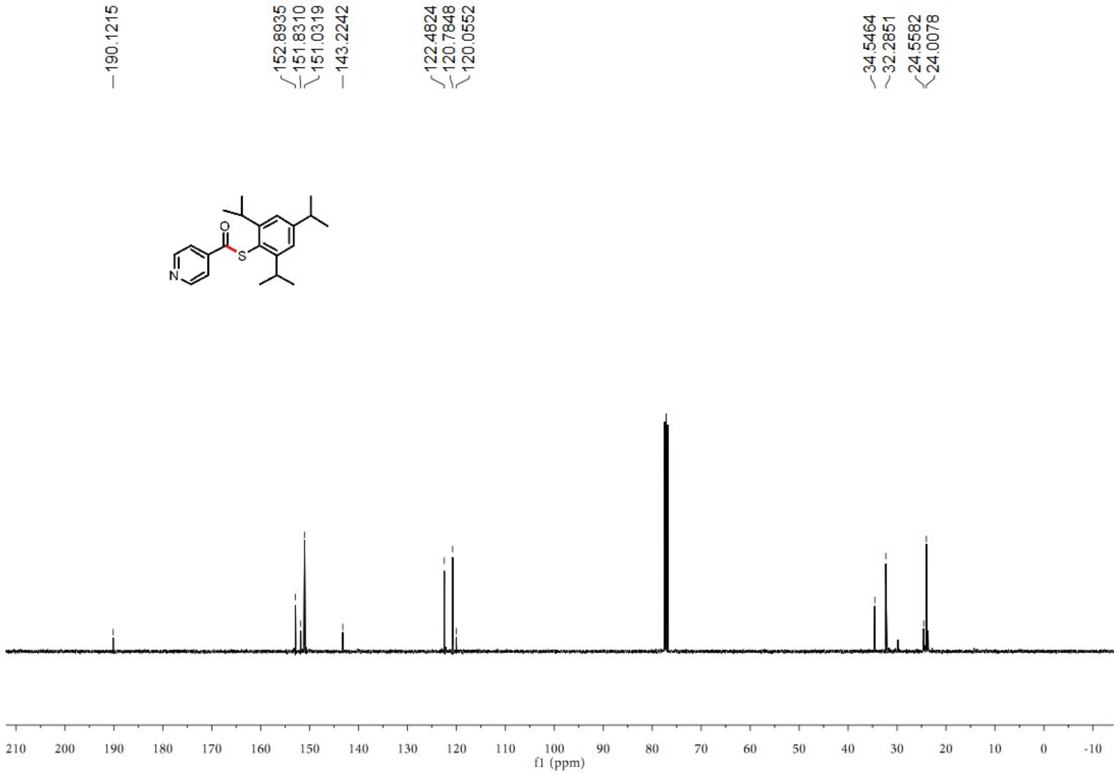

Supplement: SC-011-C9SC05132E-s001 [file SC-011-C9SC05132E-s001.pdf]
